# Highly homogeneous antibody modification through optimisation of the synthesis and conjugation of functionalised dibromopyridazinediones†
†Electronic supplementary information (ESI) available. See DOI: 10.1039/c7ob03138f


**DOI:** 10.1039/c7ob03138f

**Published:** 2018-01-29

**Authors:** Calise Bahou, Daniel A. Richards, Antoine Maruani, Elizabeth A. Love, Faiza Javaid, Stephen Caddick, James R. Baker, Vijay Chudasama

**Affiliations:** a Department of Chemistry , University College London , London , UK . Email: j.r.baker@ucl.ac.uk ; Email: v.chudasama@ucl.ac.uk; b LifeArc , Accelerator Building , Open Innovation Campus , Stevenage , UK; c Research Institute for Medicines (iMed.ULisboa) , Faculty of Pharmacy , Universidade de Lisboa , Lisbon , Portugal

## Abstract

Due to their exquisite cysteine-selectivity, excellent stability, and ability to functionally rebridge disulfide bonds, dibromopyridazinediones are emerging as an exciting new class of bioconjugation reagents, particularly in the field of antibody conjugation. Despite this, relatively little work has been performed on the optimisation of their synthesis and subsequent reaction with immunoglobulins. Herein we present a novel synthetic route towards functionalised dibromopyridazinediones, proceeding *via* an isolatable dibromopyridazinedione-NHS ester. Reaction of this activated intermediate with a variety of amines produces functional dibromopyridazinediones in good to excellent yields. The disulfide rebridging capacity of these reagents was optimised on the clinically relevant IgG1 trastuzumab, resulting in a general method which allows for the generation of site-selectively modified native trastuzumab with over 90% homogeneity (no disulfide scrambling) without the need for protein engineering or enzymatic conjugation.

## Abstract 2

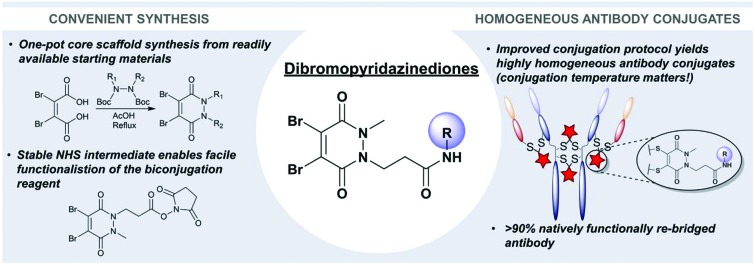
Herein we report novel protocols for the generation and application of dibromopyridazinediones, an exciting class of disulfide bridging reagents.

## Introduction

Over the last few decades biologists, biochemists, and chemical biologists have put forth a concerted effort to explore and harness the unique attributes of antibodies, particularly their exquisite targeting specificity, excellent *in vivo* stability, and ability to initiate antibody-dependent cell-mediated cytotoxicity (ADCC).^1^ These properties make antibodies excellent biomolecules for a plethora of tasks, especially as therapeutic agents. Their therapeutic utility has been bolstered by advancements in hybridoma and phage display technology, which enable the rapid generation of high-affinity monoclonal antibodies against emerging diseases.^2^,^3^ Despite the many advantages of antibodies, their chemical functionality is limited to the reactivities of the 20 natural amino acids. To fully exploit antibodies for biomedical applications it is desirable to expand their reactivity beyond the capacity of the natural amino acids. To this end, significant research effort has been devoted to developing new methods for functionalising antibody scaffolds through chemical conjugation.^4^–^7^ The chemical modification of antibodies has led to the development of a novel class of biologics; antibody–drug conjugates (ADCs).^8^

Despite their utility, many conjugation strategies for synthesising ADCs suffer from several drawbacks. The targeting of amino acids frequently provides no regioselectivity over the modification when trying to aim for drug loadings of 2–4 (commonly tolerated drug loadings in terms of pharmacokinetics), leading to heterogeneous product mixtures. This is particularly true when targeting lysine residues, which are highly abundant on the solvent accessible surface of antibodies.^8^ Despite this, lysine conjugation is employed for the generation of clinically approved ADCs Kadcyla® and Besponsa®.^9^,^10^ To overcome the issue of lysine over-abundance, cysteine-selective reagents are frequently employed. Although native antibodies contain no solvent accessible reactive cysteine residues, liberation of cysteine residues from reduction of the inter-chain disulfide bonds provides suitable targets. Unfortunately, this reduction results in a loss of the stabilising disulfide bridges and, in the instance of IgG1 antibodies, generates up to 8 reactive cysteine residues – factors which can contribute to heterogeneity when targeting typically tolerated loadings of 2–4 drugs per antibody.^8^ Conjugation to liberated cysteine residues was employed for the generation of the ADC Adcetris®.^11^ The issues of uncontrolled conjugation are particularly problematic for therapeutic applications, where suboptimal drug loading can lead to poor pharmacokinetic properties and a narrow therapeutic window.^12^ As such, research focus has shifted to developing technologies which allow for site-specific conjugation. This is commonly achieved using protein engineering to install a site-specific cysteine residue or unnatural amino acid, or through enzymatic processes.^8^ Whilst these methods work well, their accessibility is limited as the process must be optimised for each individual antibody; a time-consuming and expensive process.

**Fig. 1:**
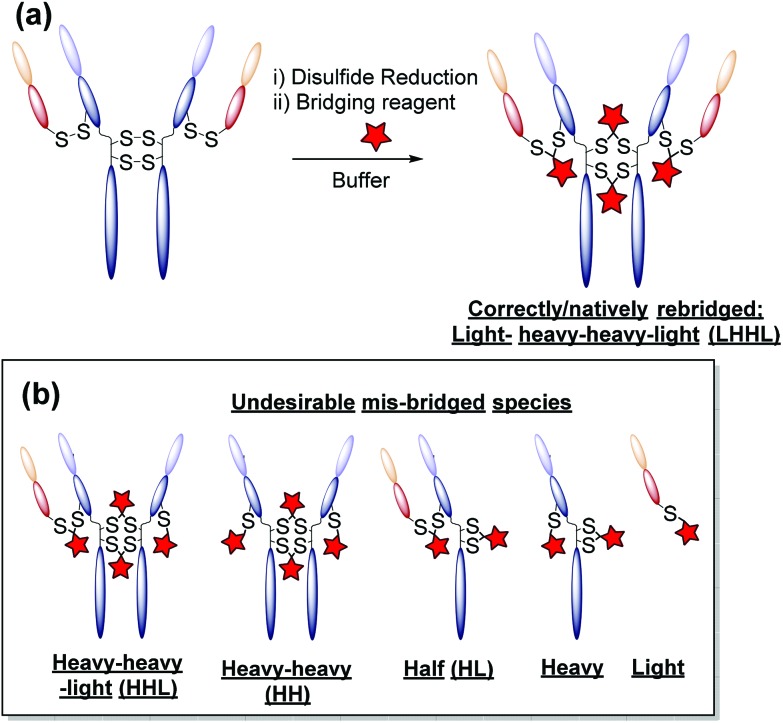
(a) An ideal disulfide bridging reaction in which interchain disulfide bonds are correctly bridged. (b) Schematic representations of the undesirable mis-bridged side-products generated with most disulfide bridging reagents.

One promising alternative approach utilises reagents that are capable of functionally rebridging the cysteine residues liberated from reduction of the disulfide bonds of *native* antibodies. This provides site-selectivity, a generally tolerated drug loading of 4 (as IgG1s contain only 4 solvent accessible disulfide bonds), and allows for retention of the covalent stabilising bridge. The most commonly employed disulfide bridging reagents include next-generation maleimides and bis-sulfones.^13^–^21^ Whilst these reagents represent important steps towards developing homogeneous antibody constructs without the need for protein engineering, improvements are still needed. For example, using reagents that show: (i) no (or at least minimal) cross-reactivity with common reducing agents; (ii) do not require a hydrolysis step to confer serum stability; and/or (iii) do not afford appreciable amounts of incorrectly functionally rebridged species such as half antibody (HL) conjugates (see Fig. 1).^13^,^15^

Most recently, dibromopyridazinediones (diBrPDs) have emerged as a class of disulfide-bridging reagents with great potential as they do not require a hydrolysis step to afford serum stability and are tolerant of/compatible with common mild reducing reagents. Modification with diBrPDs grants good levels of homogeneity (especially with bespoke reagents), long term blood plasma stability, and has no detectable effect on the binding capability of the parent antibody.^22^–^25^ To date, the diBrPD linker platform has been employed extensively, *e.g.* in the generation of ADCs,^22^,^23^ antibody conjugates,^24^,^26^ antibody-directed photosensitisers,^27^,^28^ protein–protein conjugates,^29^ and a targeted nanotherapeutic.^30^

**Scheme 1:**
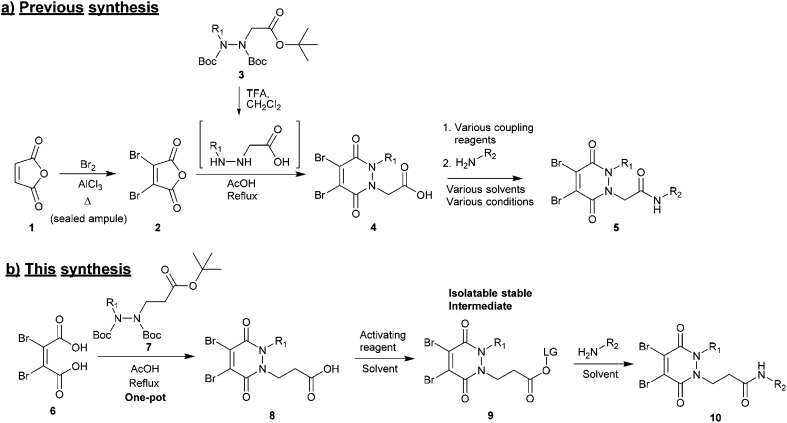
(a) Current synthesis of diBrPDs **4** from maleic anhydride **1** and hydrazines **3**, followed by amide coupling. (b) Proposed one-pot synthesis of diBrPDs **8** from dibromomaleic acid **6** and hydrazines **7**, followed by generation of an isolatable activated ester **9** to enable a general route towards functionalised diBrPDs **10**.

However, whilst diBrPDs address some of the key remaining issues for disulfide rebridging reagents, significant challenges still remain in their synthesis and application. These are discussed in turn below:

(1) *Synthesis of the core scaffold*. Previously reported synthetic routes towards core diBrPD scaffolds proceed under harsh conditions that have excluded the use of certain functional groups to be appended to the scaffold.^31^,^32^ These routes require pre-formation of dibromomaleic anhydride **2***via* bromination of maleic anhydride **1**, a dangerous reaction that requires high temperature and high pressure. We note that whilst dibromomaleic anhydride **2** may be purchased, it has very limited availability with suppliers and can be prohibitively expensive (£908 per g through Sigma Aldrich). These routes also employ trifluoroacetic acid (TFA) deprotection of the hydrazine, prior to dehydration, increasing costs and environmental impact, and decreasing overall atom efficiency (Scheme 1a).^26^

(2) *Functionalisation of the core scaffold in good and consistent yields via a general protocol*. A common method for adding functionality to functional rebridging reagents is the reaction of an amine with an activated carboxylic acid. The resultant amide bond shows excellent stability *in vivo*, and a great deal of toxic payloads, fluorescent dyes, and imaging agents are commercially available as amines. Previously, carboxylic acid functionalised diBrPDs **4** have been coupled with amines using a variety of activating reagents.^22^–^24^,^26^,^27^,^30^ Whilst this demonstrates a broad scope for forming functionalised diBrPD reagents, it is also indicative of how capricious the reaction is; for each new amine a different activating reagent was employed and specific reaction conditions developed. This precludes the use of a generalised synthetic pathway to these useful reagents (Scheme 1a).

(3) *A general protocol for homogeneous antibody conjugation without having to use bespoke reagents*. We have previously reported a pyridazinedione-based reagent with built-in reductive capacity that is capable of both reducing and functionally rebridging the inter-chain disulfide bonds of an antibody.^26^ Whilst this reagent grants antibody conjugates with unprecedented homogeneity, and a desirable drug-to-antibody ratio of 4 (DAR), the synthetic difficulty and poor long-term stability of the reagent without storing in the freezer under argon limits wide-spread adoption. Methods which allow for similar levels of homogeneity (low levels of mis-bridged species – see Fig. 1) and DAR without the associated synthetic difficulty and oxidation instability are sought after.

Herein we report a general and novel synthetic route to functionalised diBrPDs **10** proceeding *via* an isolatable activated ester **9**, incorporating a one-pot synthesis for the formation of the core scaffold. This procedure was utilised to generate a small library of functional diBrPDs (Scheme 1b). The subsequent conjugation of these reagents was then optimised on the clinically relevant antibody trastuzumab, and found to proceed with excellent and unprecedented homogeneity.

## Results and discussion

### Pyridazinedione (PD) core scaffold synthesis

**Fig. 2:**
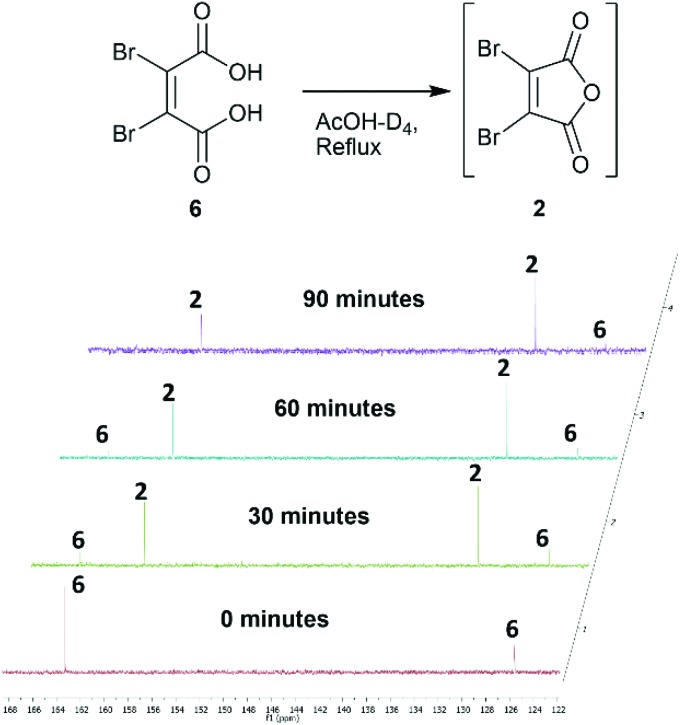
^13^C NMR spectra showing formation of dibromomaleic anhydride **2** from dibromomaleic acid **6** in boiling deuterated acetic acid (AcOH-D_4_).

Towards the development of a one-pot synthesis of the core diBrPD scaffold, initial focus was given to the *in situ* formation of dibromomaleic anhydride **2** from dibromomaleic acid **6** (£18 per g through Sigma Aldrich). Dibromomaleic acid **6** has been successfully reacted with amines for the synthesis of dibromomaleimides; a synthetic pathway similar to the generation of diBrPDs. We proposed that this reaction was proceeding *via* dibromomaleic anhydride **2**, formed through the ring-closing dehydration of dibromomaleic acid **6** under acid conditions. This theory was explored by refluxing dibromomaleic acid **6** in deuterated acetic acid (AcOH-D_4_). Following the reaction using ^13^C NMR demonstrated almost full conversion to dibromomaleic anhydride **2** within 30 minutes (Fig. 2).

**Scheme 2:**
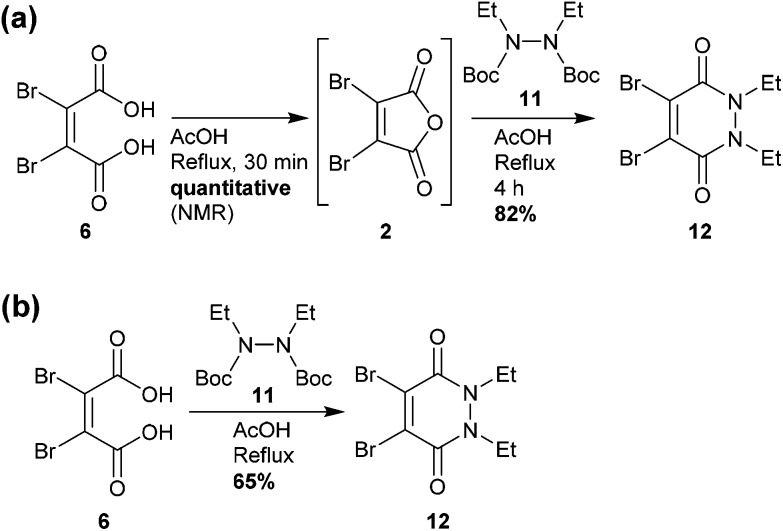
(a) Synthesis of diethyl dibromo PD **12** using commercially available dibromomaleic acid **6** (b) Synthesis of diethyl dibromo PD **12** using dibromomaleic acid **6** refluxed for 1 h prior to addition of diethyl diboc hydrazine **11**.

Similarly, analysis by thin-layer chromatography (TLC) indicated rapid deprotection of diethyl diboc hydrazine **11** within 10 minutes of boiling in acetic acid. A protocol was developed in which dibromomaleic acid **6** was refluxed in acetic acid for 30 min prior to addition of diethyl diboc hydrazine **11**, followed by a further 4 hours of reflux (Scheme 2a). This produced diBrPD **12** in an 82% yield, higher than the overall yield of 68% granted by the previously reported protocol. To explore the importance of the preformation of dibromomaleic anhydride **2**, the reaction was performed with all reagents added at the start in a one-pot synthesis. A decreased yield of 65% was observed, indicating that preformation of dibromomaleic anhydride **2** is key to achieving high yields in this instance (Scheme 2b). This new route reduces the complexity of the synthesis of the diBrPD scaffold, increases overall yield and step-economy, decreases the number of reagents required, and eliminates the dangerous and cumbersome bromination reaction of maleic anhydride through the use of relatively cheap and readily commercially available dibromomaleic acid **6**.

### Development of pyridazinedione-*N*-hydroxysuccinimide activated ester

**Scheme 3:**
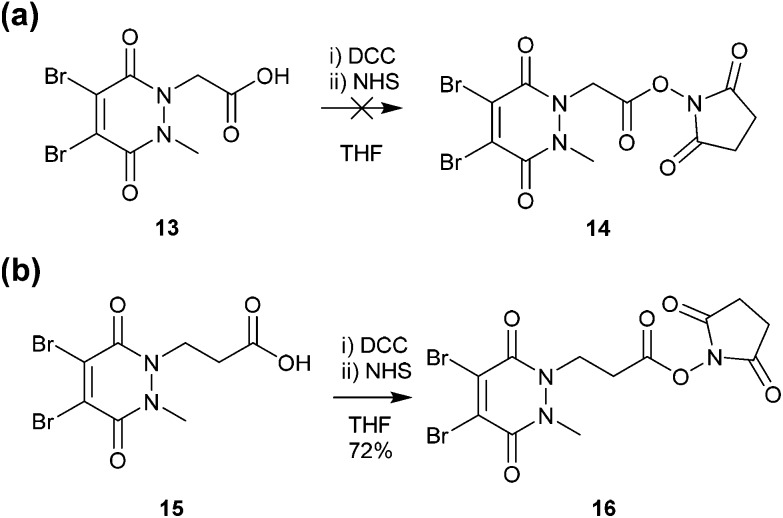
(a) Attempted synthesis of NHS-activated methyl ethanoic acid diBrPD **14** (b) Synthesis of NHS activated methyl propionic acid diBrPD **16**.

Methyl ethanoic acid diBrPD **13** has been previously employed to generate functionalised diBrPDs through amide coupling reactions with amines. However, the reaction has been shown to be capricious and amine-dependent, requiring the development of tailored conditions for each coupling reaction (see introduction). To create a more generalised route towards functionalised diBrPDs we attempted to synthesise and isolate a reactive NHS-ester intermediate of diBrPD **14**. It was hoped this intermediate could be synthesised in large quantities, stored for extended periods, and be used in a general protocol towards functionalised diBrPDs. To this end, we attempted to synthesise the NHS ester of methyl ethanoic acid diBrPD **13** by reacting it with with DCC and NHS. Whilst this resulted in complete consumption of starting material, isolation of the desired species **14** was not possible under various conditions despite multiple attempts. This may go some way to explaining why the amide coupling reactions using methyl ethanoic acid diBrPD **13** were so capricious. It was hypothesised that degradation of the intermediate was occurring through intramolecular reaction of the activated species with the carbonyl of the pyridazinedione ring (possibly *via* a 6-membered ring transition state). To appraise this, we synthesised methyl propionic acid diBrPD **15**, in which the acid is separated from the ring system by a 2-carbon alkyl chain, *i.e.* leading to what would be an undesirable 7-membered ring transition state. Pleasingly, subjecting methyl propionic acid diBrPD **15** to analogous NHS ester formation conditions led to the generation of activated diBrPD **16** in a good yield (Scheme 3). We were also pleased that we were able to synthesise methyl propionic acid diBrPD **15** using the core scaffold synthesis outlined above (see ESI Scheme S2†).

With activated diBrPD **16** in hand, we examined its propensity to form amides upon reaction with a library of diverse amines. The reactions proceeded with good to excellent yields with amines containing hydrophobic, hydrophilic, terminal alkyne and strained alkyne functional groups all being tolerated (Table 1). A secondary amine was also reactive towards diBrPD **16** producing amide **17c** in good yield; a feat not feasible for reaction with methyl ethanoic acid diBrPD **13**. Moreover, synthesis of diBrPD-amide **17f** proceeded with a high yield of 78%; this contrasts starkly with the 8% yield obtained for the same coupling reaction when using previous methyl ethanoic acid diBrPD **13** (Table 1, Reaction F).^24^

**Table 1:** Reaction of activated NHS-activated diBrPD **16** with a library of amines

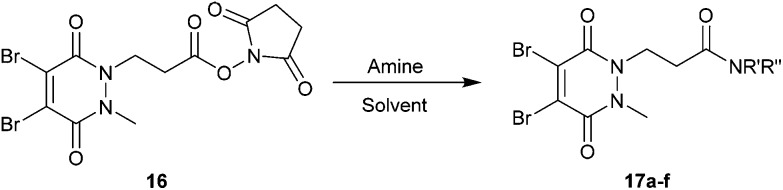				
Reaction	Solvent	Amine	Product number	Yield
A	CH_2_Cl_2_	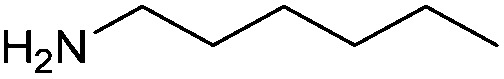	**17a**	78%
B	CH_2_Cl_2_	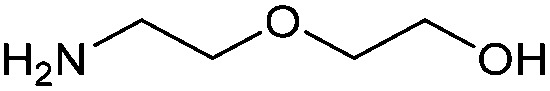	**17b**	71%
C	MeCN	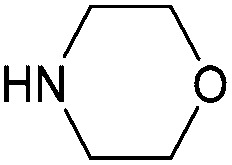	**17c**	76%
D	MeCN	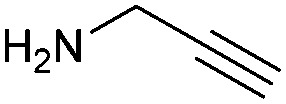	**17d**	87%
E	MeCN	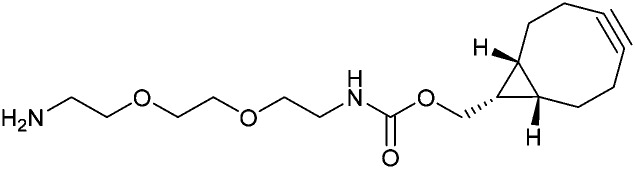	**17e**	72%
F	MeCN	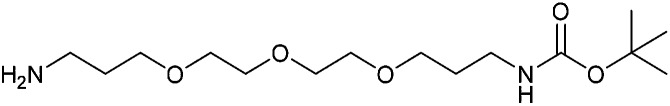	**17f**	78%

These results demonstrate the utility of activated diBrPD **16**, a reagent which enables a general route towards the functionalisation of the core diBrPD scaffold. Furthermore, this compound was found to be stable at –20 °C for at least 3 months and not prone to oxidation or intramolecular decomposition; increasing its applicability as a building block for the synthesis of functionalised diBrPD reagents (ESI Fig. S11†).

## Antibody bioconjugation

### Optimisation

The monoclonal antibody trastuzumab **18**, an FDA-approved therapeutic for HER2+ breast cancers,^33^ was chosen as a model to appraise and optimise the conjugation of functional diBrPD reagents **17a–e**. Trastuzumab **18** is also employed within the clinically approved ADC Kadcyla®,^9^ and multiple pre-clinical therapeutics,^20^,^34^,^35^ and thus was deemed an appropriate model system. As discussed above, typical disulfide bridging reagents yield some mis-bridged species in addition to the desired fully re-bridged antibody (see introduction). The only protocol that does not suffer from this issue requires intricate synthesis of a complex reagent that is prone to oxidation.^26^ We set out to develop a protocol using diBrPD reagents which could minimise these undesirable mis-bridged species without the use of complex reagents. A key objective was to hinder formation of the mis-bridged “half antibody” species (Fig. 1), in which the hinge disulfides become scrambled and the bridging reagent adds across the intra-chain cysteines rather than inter-chain. Therefore, an optimisation study with these reagents to determine the best conditions for obtaining high levels of homogeneity and a pyridazinedione-to-antibody ratio (PDAR) of 4 was performed.

We first explored the relationship between the two reactions steps, reduction and rebridging, to determine how the order of reagent addition would affect the composition of the final product. There are 3 generally accepted functional rebridging strategies: (1) a step-wise protocol in which reduction is performed first, followed by removal of the reducing agent and addition of the bridging reagent; (2) a sequential protocol in which reduction is followed by addition of the bridging reagent without intermediate purification to remove the reducing reagent; and (3) an *in situ* protocol in which the bridging reagent is added before addition of the reducing agent. Due to cross-reactivity with commonly employed reducing agents, many disulfide bridging reagents require a step-wise protocol. It has been noted previously that diBrPDs are amenable to both sequential and *in situ* protocols due to a lack of (or minimal) cross-reactivity with the commonly employed reducing agent tris(2-carboxyethyl)phosphine (TCEP) under the reaction conditions.^26^ These 3 strategies were compared for the modification of trastuzumab **18** using synthesised strained alkyne diBrPD **17e**. This reagent was chosen for this study due to its chemical complexity; we wanted to appraise the platform with a chemical linker which contained both hydrophilic and hydrophobic components, as well as reactive functionalities.

**Fig. 3:**
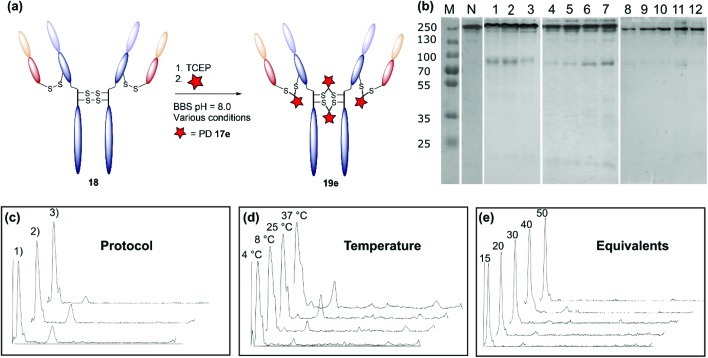
Optimisation of the disulfide bridging of trastuzumab using diBrPD **17e**. Unless otherwise specified, a concentration of 20 μM of trastuzumab was employed. Unless other specified, 10 equivalents of TCEP·HCl and 20 equivalents of PD **17e** were employed. For full details of each protocol refer to ESI.† (a) Schematic representation of the generation of a PDAR 4 trastuzumab conjugate. (b) SDS-PAGE gel: M: molecular weight protein marker in kDa; N: native trastuzumab **18**; 1: step-wise protocol; 2: sequential protocol; 3: *in situ* protocol; 4: *in situ* protocol 4 °C; 5: *in situ* protocol 8 °C; 6: *in situ* protocol 25 °C; 7: *in situ* protocol 37 °C; 8: *in situ* protocol 15 equivalents of diBrPD **17e**; 9: *in situ* protocol 20 equivalents of PD **17e**; 10: *in situ* protocol 30 equivalents of PD **17e**; 11: *in situ* protocol 40 equivalents of diBrPD **17e**; 12: *in situ* protocol 50 equivalents of diBrPD **17e**. (c) Densitometry traces showing the effect of protocol on bridging efficiency: (1) step-wise; (2) sequential; (3) *in situ*. (d) Densitometry traces showing the effect of temperature (4 °C, 8 °C, 25 °C, and 37 °C) on bridging efficiency. (e) Densitometry traces showing the effect of equivalents of diBrPD **17e** (15, 20, 30, 40, and 50 equivalents) on bridging efficiency.

The step-wise protocol reduction was achieved by incubation of trastuzumab **18** with TCEP, followed by purification *via* ultrafiltration and subsequent addition of diBrPD **17e**. A similar approach was employed for the sequential protocol but with no purification before addition of diBrPD **17e**. Finally, the *in situ* protocol involved addition of diBrPD **17e** prior to addition of TCEP. In each case, 10 equivalents of reducing agent (*i.e.* 2.5 eq. per disulfide) and 20 equivalents of diBrPD **17e** (*i.e.* 4 eq. per disulfide) were employed (Fig. 3c). From the results, it is clear to see that whilst all 3 protocols granted a desired PDAR of 4, both the step-wise and sequential protocols yielded significantly poorer functional rebridging; SDS-PAGE and densitometry analysis show greater levels of half antibody species when compared to the *in situ* protocol. We hypothesise that this result is due to the relative kinetics of the two reactions steps (reduction and rebridging); reduction of the disulfide bond using TCEP may be slower than the subsequent reaction of the liberated cystine residues with diBrPD **17e**. The presence of a high concentration of diBrPD **17e***in situ* allows the rebridging step to occur almost immediately after reduction whilst the antibody domains are still templated by various intermolecular forces. Conversely, both step-wise and sequential protocols allow enough time for some loss of the antibody template, leading to significant mis-bridging of the liberated cysteine residues upon addition of the bridging reagent. This result is in accordance with the previously reported two-in-one pyridazinedione reagent.^26^

We next studied the effect of temperature on the bridging efficiency. Based on the previous results, an *in situ* protocol was utilised, with 10 equivalents of TCEP and 20 equivalents of diBrPD **17e**. Incubation over 16 hours at 4 °C, 8 °C, 25 °C and 37 °C highlighted a significant temperature dependence on the overall composition of the final construct (Fig. 3d): in each case, a PDAR of 4 was observed, though increasing levels of heterogeneity were observed as the temperature was increased. This could be due to the higher temperature increasing the rate at which the antibody loses its template upon reduction, or by increasing the rate of TCEP reduction itself. Regardless, these results clearly show that lower temperatures are beneficial to increasing the homogeneity of the final constructs. To the best of our knowledge the effect of temperature on the reactions of disulfide bridging reagents has not been reported. We anticipate that this discovery will be transferable to other reagents and thus prove useful throughout the field in a general sense.

Finally, we looked at the effect of equivalents of diBrPD **17e** on bridging efficiency. Employing an *in situ* protocol at 4 °C, we reacted 15, 30, 40, and 50 equivalents of diBrPD **17e** with trastuzumab **18**. In each case the amount of organic solvent (DMSO) included to solubilise the linker remained constant. These results show that increasing the equivalents of diBrPD **17e** does not grant greater levels of homogeneity; in fact, increasing the amount of diBrPD **17e** beyond 30 equivalents granted lower PDAR values (Table 2). During these reactions a visible precipitate was observed, attributed to precipitation of the linker, which may account for the observation. Whilst the SDS-PAGE gels of these experiments show high homogeneity, the corresponding low PDAR values indicate this could be down to re-oxidation of the disulfide bridges rather than functional rebridging. Gratifyingly, these results demonstrate that as low as 15 equivalents of diBrPD **17e** can be employed without sacrificing bridging efficiency and the homogeneity of the final product.

**Table 2:** Optimisation of the disulfide rebridging of trastuzumab **18** with diBrPD **17e**

	Protocol			Temperature/°C				Equivalents of diBrPD x				
	1	2	3	4	8	25	37	15	20	30	40	50
% Half antibody^*a*^	23	20	8	3.5	4.8	14.1	18.5	6.0	4.8	4.0	0^*c*^	1.7^*c*^
PDAR^*b*^	4.1	4.1	3.9	4.2	3.8	3.9	4.2	3.9	3.9	3.9	3.6	3.3

^*a*^Calculated from densitometry traces in Fig. 4 using Image J. See ESI for details.

^*b*^PDAR was calculated using UV-vis spectrophotometry. See ESI Fig. S19–S21.

^*c*^Low PDAR values indicate these values may not be representative. See discussion above.

### Validation of an optimised protocol

**Fig. 4:**
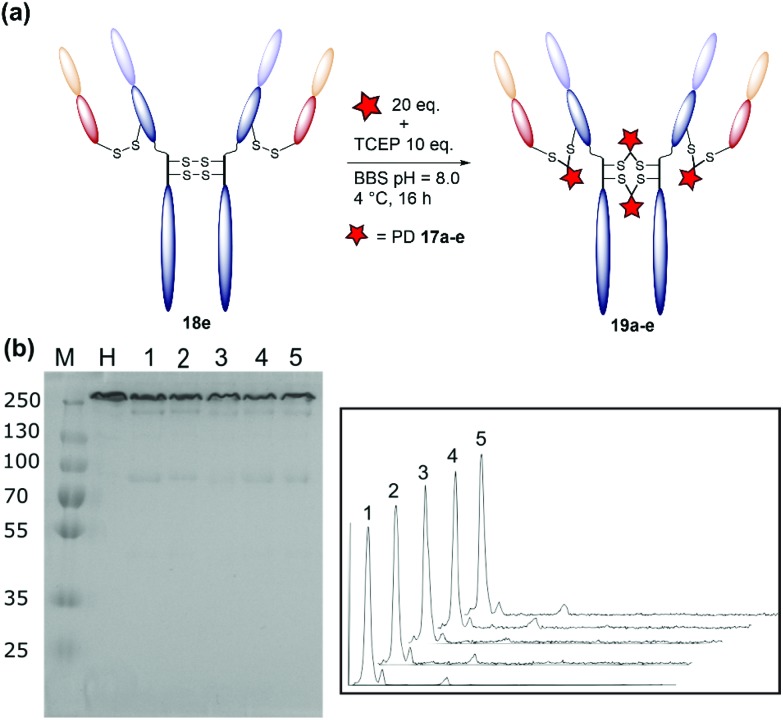
(a) Schematic representation of the rebridging of trastuzumab **18** with diBrPDs **17a–e** under optimised conditions. (b) SDS-PAGE gel: M: molecular weight protein marker in kDa; H: native trastuzumab **18**; 1: bridging with diBrPD **17a**; 2: bridging with diBrPD **17b**; 3: bridging with diBrPD **17c**; 4: bridging with diBrPD **17d**; 5: bridging with diBrPD **17e**. (c) Densitometry traces of SDS-PAGE lanes 1–5.

The result of the optimisation study detailed above were combined to develop an optimised conjugation protocol of diBrPD **17e** with trastuzumab **18**. Trastuzumab **18** was mixed with 20 equivalents of diBrPD **17e** and allowed to cool to 4 °C for 2 hours. After this time, 10 equivalents of TCEP were added and the reaction incubated at 4 °C for 16 hours. Pleasingly, this led to a construct with a PDAR of 4 and a high level of homogeneity, with densitometry analysis indicating only 5% of the total antibody concentration existed as the mis-bridged half antibody. To demonstrate scope, this protocol was applied to diBrPDs **17a–d**. To our delight, in each case equally high levels of homogeneity were observed with conjugates **19a–e** along with optimal PDARs (Fig. 4, Table 3). ELISA of conjugates **19a–e** demonstrated no loss of antigen binding when compared to native trastuzumab **18** (Fig. S24†), providing evidence that modification with diBrPDs **17a–e** does not affect antigen binding. This study confirms that the protocol is compatible with linkers of varying hydrophobicity and functionalities, an important factor for the development of ADCs (Fig. 4, Table 3).

**Table 3:** Optimised reaction of diBrPD **17a–e** with trastuzumab **18**

	diBrPD **17a–e**				
	**17a**	**17b**	**17c**	**17d**	**17e**
% Half antibody^*a*^	3.3	3.8	1	6.2	4.7
PDAR^*b*^	4.2	4.1	4.0	4.0	4.0

^*a*^Calculated from densitometry traces in Fig. 4 using Image J. See ESI for details.

^*b*^PDAR was calculated using UV-vis spectrophotometry. See ESI Fig. S22.

## Conclusion

To conclude, we have developed several novel protocols to improve both the synthesis and subsequent application of functionalised dibromopyridazinediones, a promising class of disulfide functional rebridging reagents. During this work we have addressed two pressing challenges in their synthesis; the formation of the core scaffold, and subsequent amine-coupling reaction of this scaffold to enable chemical functionalisation. A one-pot synthesis of the core scaffold from readily available starting materials provides a safer and more efficient alternative to reported routes, whereas a novel isolable NHS-ester intermediate enables a more general protocol for the derivatisation of this scaffold with a variety of amines. The capacity of these reagents to functionally rebridge disulfide bonds was optimised on the humanised IgG trastuzumab, and a protocol was developed which grants high levels of homogeneity (>90%) and a pyridazinedione-to-antibody ratio of 4. Furthermore, the novel findings on the temperature of bioconjugation being critical to outcome could be a significant general find to the field of bioconjugation as a whole. Moreover, this procedure comes at minimal expense and complexity when compared to previously published routes. Therefore, it is expected that the development of these protocols will vastly increase the accessibility of this exciting class of reagents, and facilitate their use within the broader scientific community.

## Conflicts of interest

There are no immediate conflicts to declare, but we make clear that VC, SC and JRB are Directors of the spin-out ThioLogics.

## Supplementary Material

Supplementary information
