# Supplementary material for: Highly homogeneous antibody modification through optimisation of the synthesis and conjugation of functionalised dibromopyridazinediones
Source: Org Biomol Chem. 2018 Jan 29;16(8):1359–66. doi: 10.1039/c7ob03138f (PMC6058253; doi:10.1039/c7ob03138f)
Supplement: Supplementary file 1 [file OB-016-C7OB03138F-s001.pdf]

Electronic Supplementary Material (ESI) for Organic & Biomolecular Chemistry.
This journal is © The Royal Society of Chemistry 2018

General Experimental:

All chemical reagents were purchased from Sigma Aldrich, Alfa Aesar and Acros. Compounds and solvents were used as received. Petrol refers to petroleum ether (b.p. 40–60 °C). All reactions were carried out under positive pressure of argon, unless stated otherwise, and were monitored using thin layer chromatography (TLC) on pre-coated silica gel plates (254 µm). Flash column chromatography was carried out with pre-loaded GraceResolv™ flash cartridges on a Biotage® Isolera Spektra One flash chromatography system. ^{1}H NMR spectra were obtained at 300 MHz, 400 MHz, 500 MHz or 600 MHz. ^{13}C NMR spectra were obtained at 125 MHz or 150 MHz. All results were obtained using Bruker NMR instruments, the models are as follows: Avance III 600, DRX500, Avance III 400, Avance 300. All samples were run at the default number of scans and at 21 °C. Chemical shifts (δ) for ^{1}H NMR and ^{13}C NMR are quoted relative to residual signals of the solvent on a parts per million (ppm) scale. Where amide rotamers are the case, and when possible, only the major rotamer has been assigned for chemical shifts, and areas underneath all rotameric peaks have been considered for integration calculations. Coupling constants (*J* values) are reported in Hertz (Hz) and are reported as $J_{H\text{-}H}$ couplings. Infrared spectra were obtained on a Perkin Elmer Spectrum 100 FTIR spectrometer operating in ATR mode. Mass spectra were obtained, for synthetic products, from the UCL mass spectroscopy service on either a Thermo Finnigan MAT900Xp (EI and CI) or Waters LCT Premier XE (ES) mass spectrometer. Melting points were measured with Gallenkamp apparatus and are uncorrected.

UV-Vis spectroscopy

UV-Vis spectroscopy was used to determine protein concentrations and pyridazinedione to antibody ratios (PDAR), using a Varian Cary 100 Bio UV-Visible spectrophotometer operating at 21 °C. Sample buffer was used as blank for baseline correction with extinction coefficients; ε_{280} = 215,000 M^{-1} cm^{-1} for trastuzumab **18** ε_{335} = 9,100 M^{-1} cm^{-1} for pyridazinedione scaffolds. A correction factor at 280 nm of 0.25 (at A_{335}) was employed for pyridazinedione scaffolds. PDAR values were calculated as previously described.[1]

SDS-PAGE & densitometry

Non-reducing glycine-SDS-PAGE at 10% acrylamide running were performed following standard lab procedures. A 4% stacking gel was used and a broad-range MW marker (10–250 kDa, Prestained PageRuler Plus Protein Standards, ThermoScientific) was co-run to estimate protein weights. Samples (10 µL at 7 µM) were mixed with loading buffer (2 µL, composition for 5 × SDS: 1 g SDS, 3 mL glycerol, 6 mL 0.5 M Tris buffer pH = 6.8, 2 mg bromophenol blue in 10 mL), heated at 75 °C for 5 minutes, and centrifuged at 16,000 RPM for 5 minutes. Samples were subsequently loaded into the wells in a volume of 5 µL. All gels were run at constant 10 mA for 15 minutes, then constant 15 mA until complete. Gels were stained using a modified Coomasie stain (25 g ammonium sulfate, 250 mg Coomasie G-250, 8.8 mL 85% ortho-phosphoric acid, 50 mL ethanol, made up to a total of 250 mL with d.d. H_2O) at room temperature for 16 h. Destained gels were imaged using a SynGene GelGenius system, with the software provided by the manufacturer. Lens aperture was set at 0.40 ms with no filter. Images were saved under default brightness, contrast, and gamma settings. Densitometry was performed using imageJ. Background subtraction was achieved using the built-in plugin with a rolling ball radius of 30, sliding paraboloid, and smoothing. Brightness and contrast settings were auto-adjusted within the software.

Enzyme-linked immunosorbent assay (ELISA)

A Nunc MaxiSorp 384-well ELISA plate was coated with HER2 diluted to a final concentration of 1 µg/mL in PBS and incubated overnight at 4 ˚C. After emptying the wells, the plate was blocked for 1 h at room temperature with 2% BSA in PBST (0.1% tween – 20). The plate was then washed 3 times with PBST, and the serially diluted test samples (22.3, 7.44, 2.48, 0.827, 0.276, 0.092, 0.031, 0.010, 0.003 nM) of tastuzumab and ADCs were added in 0.2% BSA, PBST. The plate was incubated for 1 h at room temperature, washed 3 times with PBST, and anti-human IgG, Fab-specific-HRP antibody (Sigma Aldrich, 1:5000 in 0.2% BSA, PBST) was added. After 1 h at room temperature, the plate was washed again and 20 µL enhanced K-Blue® substrate (TMB) (Neogen) was added to each well. Once colour was observed, reaction was stopped by adding 10 µL Red Stop solution. Absorbance was immediately measured at 650 nm. Controls were included in every ELISA, in which PBS had been added to some of the wells instead of HER2 or instead of antibody sample. Each sample was tested in triplicate, and errors are shown as the standard deviation of the average.

Supplementary Schemes

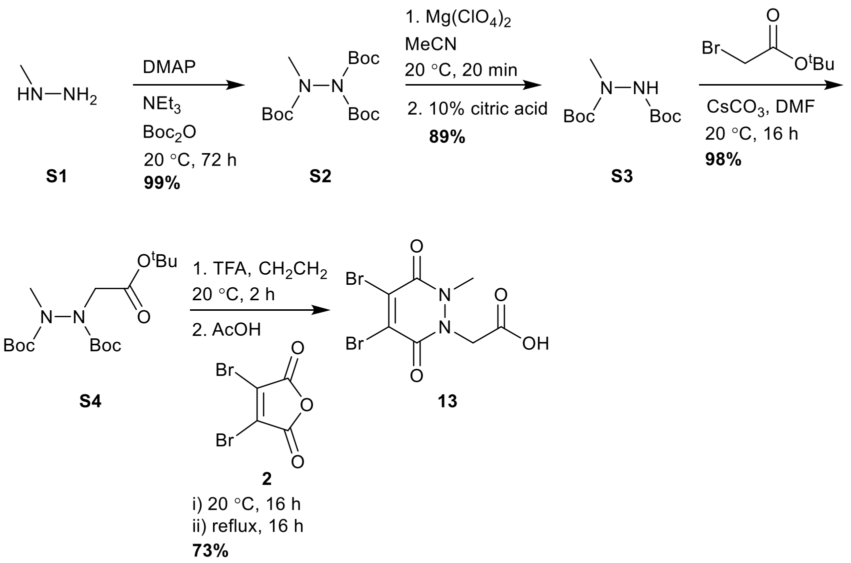

Scheme S1 Synthesis 2-(4,5-dibromo-2-methyl-3,6-dioxo-3,6-dihydropyridazin-1(2*H*)-yl)acetic acid **13**.[2]

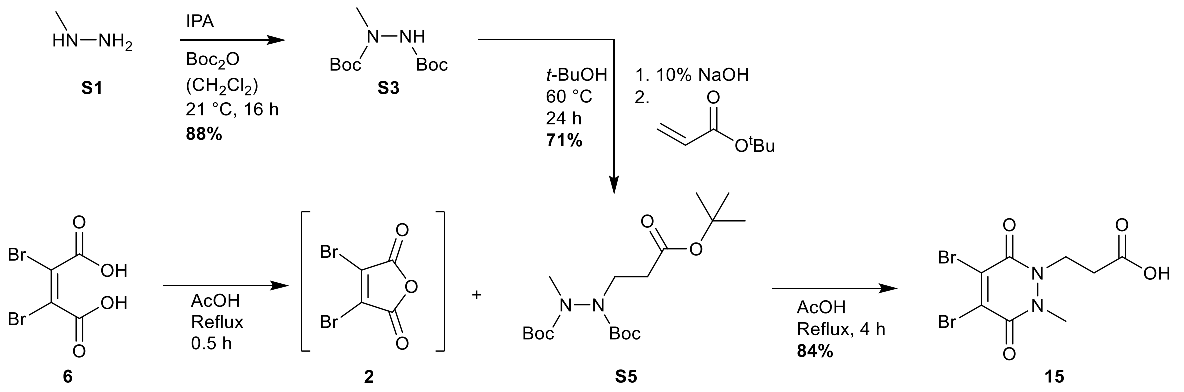

Scheme S2 Synthesis of 3-(4,5-dibromo-2-methyl-3,6-dioxo-3,6-dihydropyridazin-1(2*H*)-yl) propanoic acid **15**.

Di-*tert*-butyl 1,2-diethylhydrazine-1,2-dicarboxylate (11)[1]

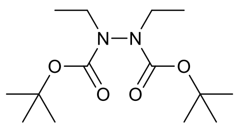

11

To a solution of di-*tert*-butyl hydrazine-1,2-dicarboxylate (1.16 g, 5.00 mmol) in DMF (20 mL) were added caesium carbonate (3.26 g, 10.0 mmol) and bromoethane (0.70 mL, 14.7 mmol). The heterogeneous mixture was stirred at 21 °C for 24 h. After this time, DMF was removed *in vacuo* with toluene co-evaporation (3 × 50 mL as an azeotrope). The crude reaction mixture was then dissolved in diethyl ether (100 mL), and then washed with water (3 × 30 mL) and saturated aq. LiCl solution (2 × 30 mL). The organic layer was dried ($MgSO_4$) and concentrated *in vacuo* to yield di-*tert*-butyl 1,2-diethylhydrazine-1,2-dicarboxylate **11** (1.21 g, 4.21 mmol, 81%) as a colourless oil: ^1H NMR (300 MHz, $CDCl_3$, rotamers) δ 3.47–3.40 (m, 4H), 1.47–1.42 (m, 18H), 1.15 (t, *J* = 7.2 Hz, 6H); ^{13}C NMR (125 MHz, $CDCl_3$, rotamers) δ 155.2 (C), 80.7 (C), 44.4 (CH_2), 28.4 (CH_3), 13.0 (CH_3); IR (thin film) 2976, 2934, 1702 cm^{-1}.

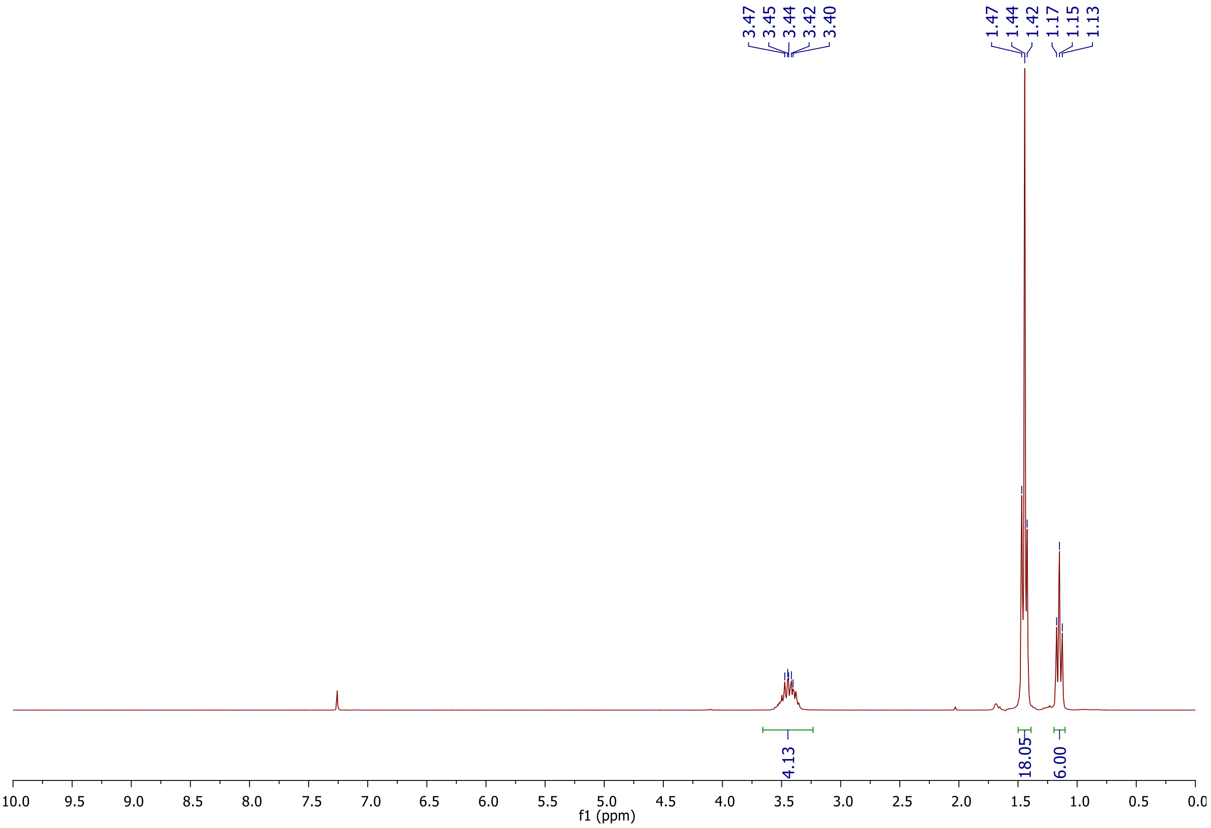

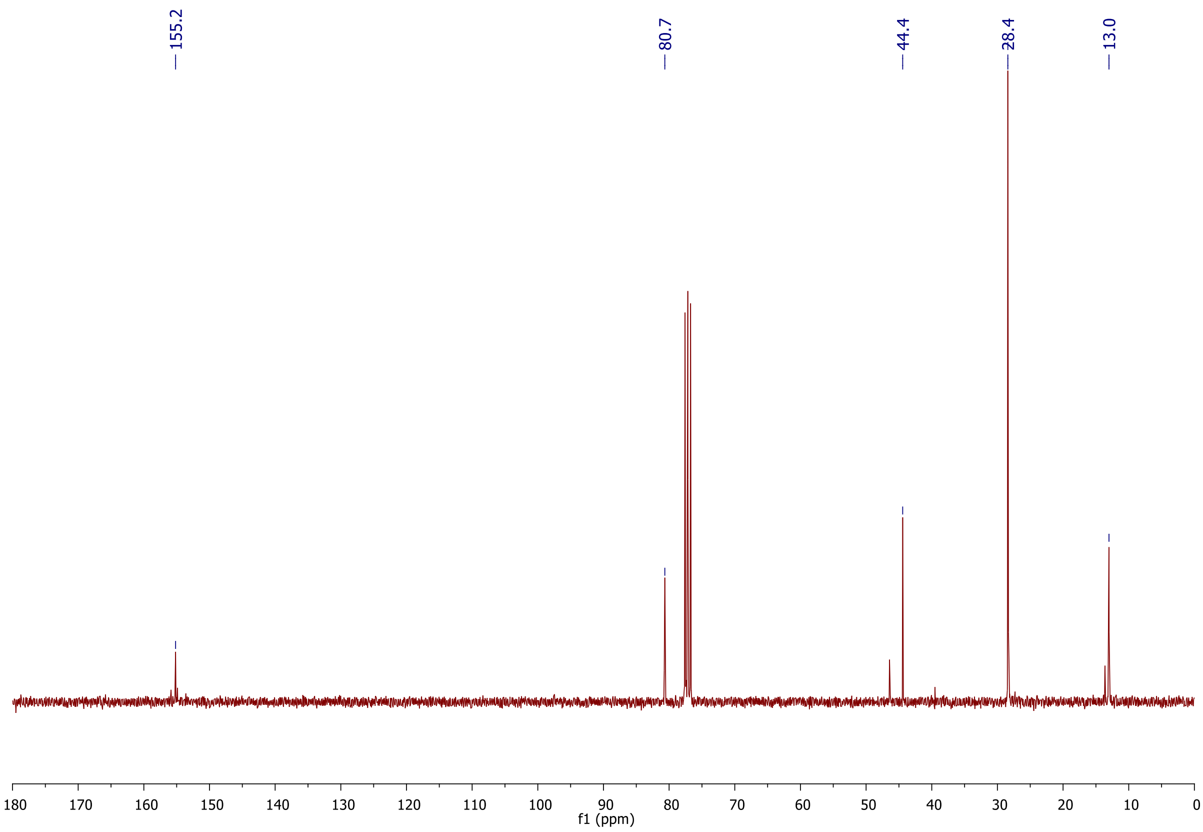

Figure S1 ^{1}H and ^{13}C NMR data for di-*tert*-butyl 1,2-diethylhydrazine-1,2-dicarboxylate **11**.

4,5-Dibromo-1,2-diethyl-1,2-dihydropyridazine-3,6-dione (12)[1]

Preformation of dibromo maleic anhydride in situ:

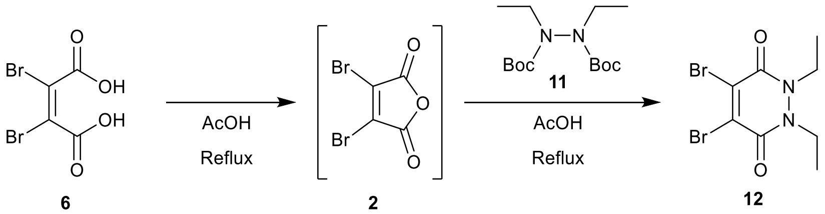

Dibromomaleic acid **6** (274 mg, 1.00 mmol) was dissolved in AcOH (10 mL) and heated under reflux for 30 min. After this time, di-*tert*-butyl 1,2-diethylhydrazine-1,2-dicarboxylate **11** (347 mg, 1.20 mmol) was added and the resultant mixture was heated under reflux for a further 4 h. After this time, the reaction mixture was concentrated *in vacuo* with toluene co-evaporation (3 × 20 mL, as an azeotrope). Purification of the crude residue by flash column chromatography (30% to 70% EtOAc/petrol) yielded 4,5-dibromo-1,2-diethyl-1,2-dihydropyridazine-3,6-dione **12** (267 mg, 0.819 mmol, 82%) as a yellow solid: m.p. 110–115 °C; ^{1}H NMR (500 MHz, $CDCl_3$) δ 4.17 (q, *J* = 7.1 Hz, 4H), 1.29 (t, *J* = 7.1 Hz, 6H); ^{13}C NMR (125 MHz, $CDCl_3$) δ 153.2 (C), 136.1 (C), 42.4 (CH_2), 13.1 (CH_3); IR (solid) 2979, 2937, 2873, 1629, 1574 cm^{-1}.

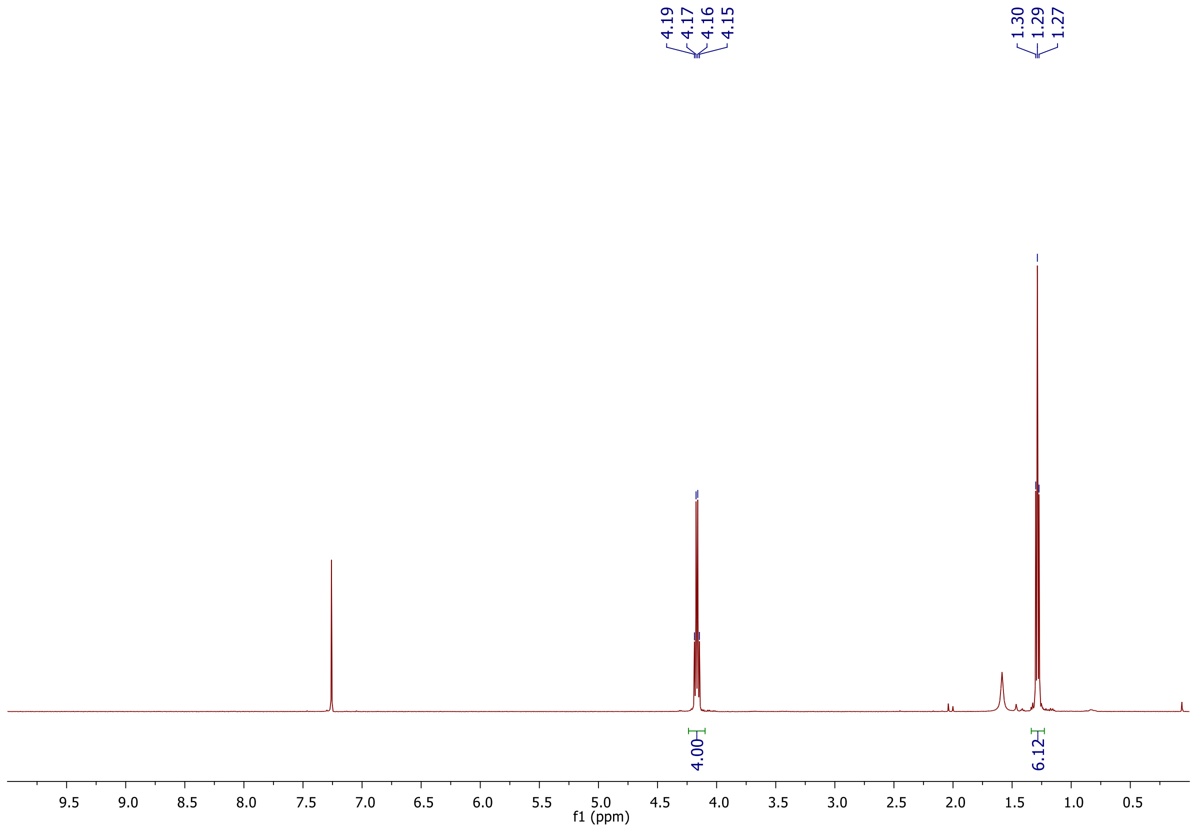

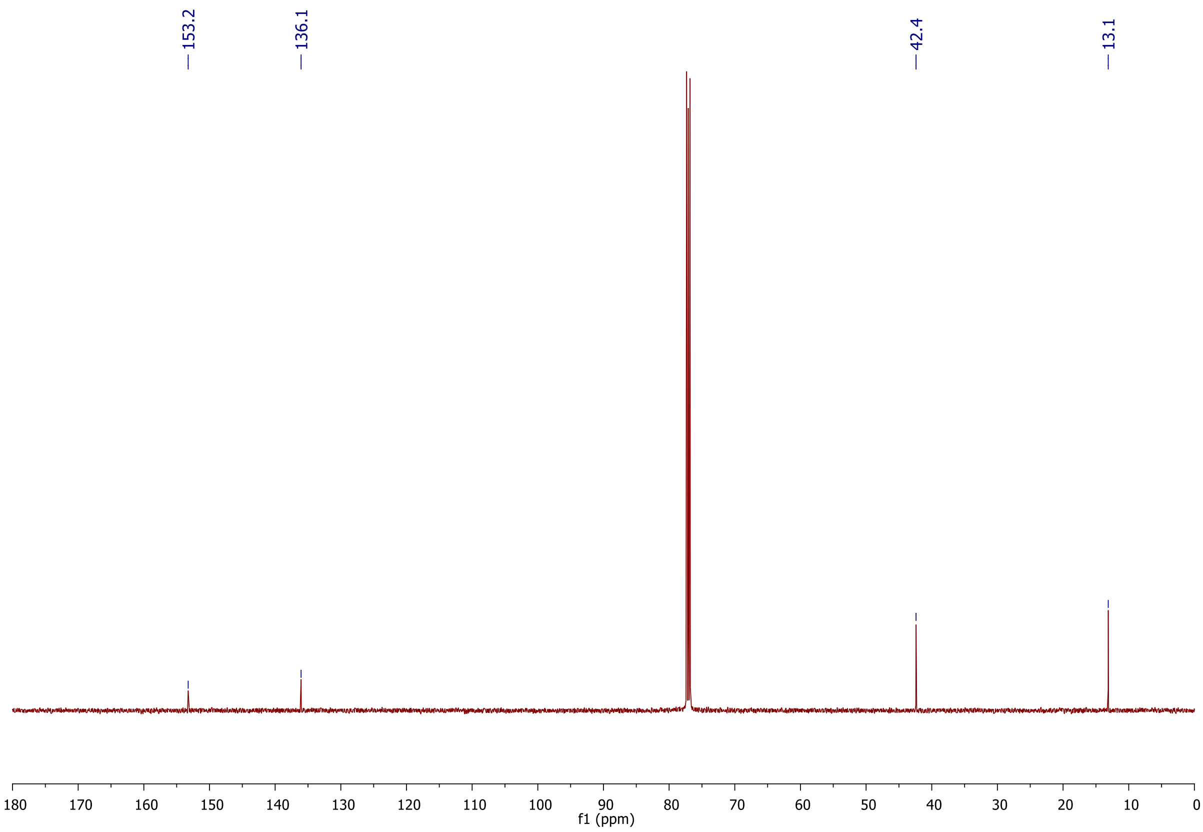

Figure S2 ^{1}H and ^{13}C NMR data for 4,5-dibromo-1,2-diethyl-1,2-dihydropyridazine-3,6-dione **12**.

No preformation of anhydride in situ:

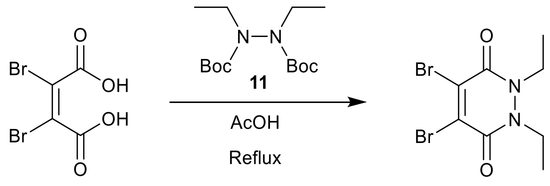

6 **12**

To a solution of dibromomaleic acid **6** (274 mg, 1.00 mmol) in AcOH (10 mL) was added di-*tert*-butyl 1,2-diethylhydrazine-1,2-dicarboxylate **11** (347 mg, 1.20 mmol) and the reaction mixture heated under reflux for 4 h. After this time, the reaction mixture was concentrated *in vacuo* with toluene co-evaporation (3 × 20 mL, as an azeotrope). Purification of the crude residue by flash column chromatography (30% to 70% EtOAc/petrol) yielded 4,5-dibromo-1,2-diethyl-1,2-dihydropyridazine-3,6-dione **12** (210 mg, 0.65 mmol, 65%) as a yellow solid: m.p. 110–115 °C; ^{1}H NMR (500 MHz, $CDCl_3$) δ 4.17 (q, *J* = 7.1 Hz, 4H), 1.29 (t, *J* = 7.1 Hz, 6H); ^{13}C NMR (125 MHz, $CDCl_3$) δ 153.2 (C), 136.1 (C), 42.4 (CH_2), 13.1 (CH_3); IR (solid) 2979, 2937, 2873, 1629, 1574 cm^{-1}.

From dibromo maleic anhydride:

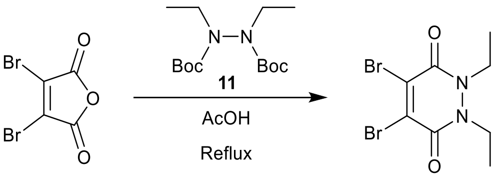

2 **12**

To a solution of dibromomaleic anhydride **2** (258 mg, 1.00 mmol) in AcOH (10 mL), was added di-*tert*-butyl 1,2-diethylhydrazine-1,2-dicarboxylate **11** (347 mg, 1.20 mmol) and the reaction mixture heated under reflux for 4 h. After this time, the reaction mixture was concentrated *in vacuo* with toluene co-evaporation (3 × 20 mL, as an azeotrope). Purification of the crude residue by flash column chromatography (30% to 70% EtOAc/petrol) yielded 4,5-dibromo-1,2-diethyl-1,2-dihydropyridazine-3,6-dione **12** (250 mg, 0.77 mmol, 77%) as a yellow solid: m.p. 110–115 °C; ^{1}H NMR (500 MHz, $CDCl_3$) δ 4.17 (q, *J* = 7.1 Hz, 4H), 1.29 (t, *J* = 7.1 Hz, 6H); ^{13}C NMR (125 MHz, $CDCl_3$) δ 153.2 (C), 136.1 (C), 42.4 (CH_2), 13.1 (CH_3); IR (solid) 2979, 2937, 2873, 1629, 1574 cm^{-1}.

Dibromomaleic anhydride (2) (by NMR in AcOH-D_4)

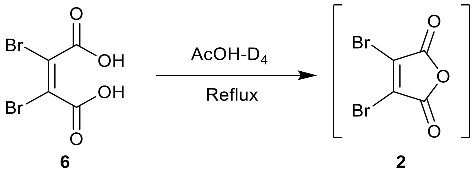

Dibromomaleic acid **6** (55 mg, 0.20 mmol) was dissolved in deuterated AcOH (2.4 mL) and the reaction heated under reflux for 90 min. The reaction mixture (0.5 mL) was cooled and analysed at 30 min intervals. Formation of dibromomaleic anhydride **2** was tracked by ^{13}C NMR. ^{13}C NMR (150 MHz, AcOH-d_4) δ 159.0 (C), 131.0 (C).

30 min:

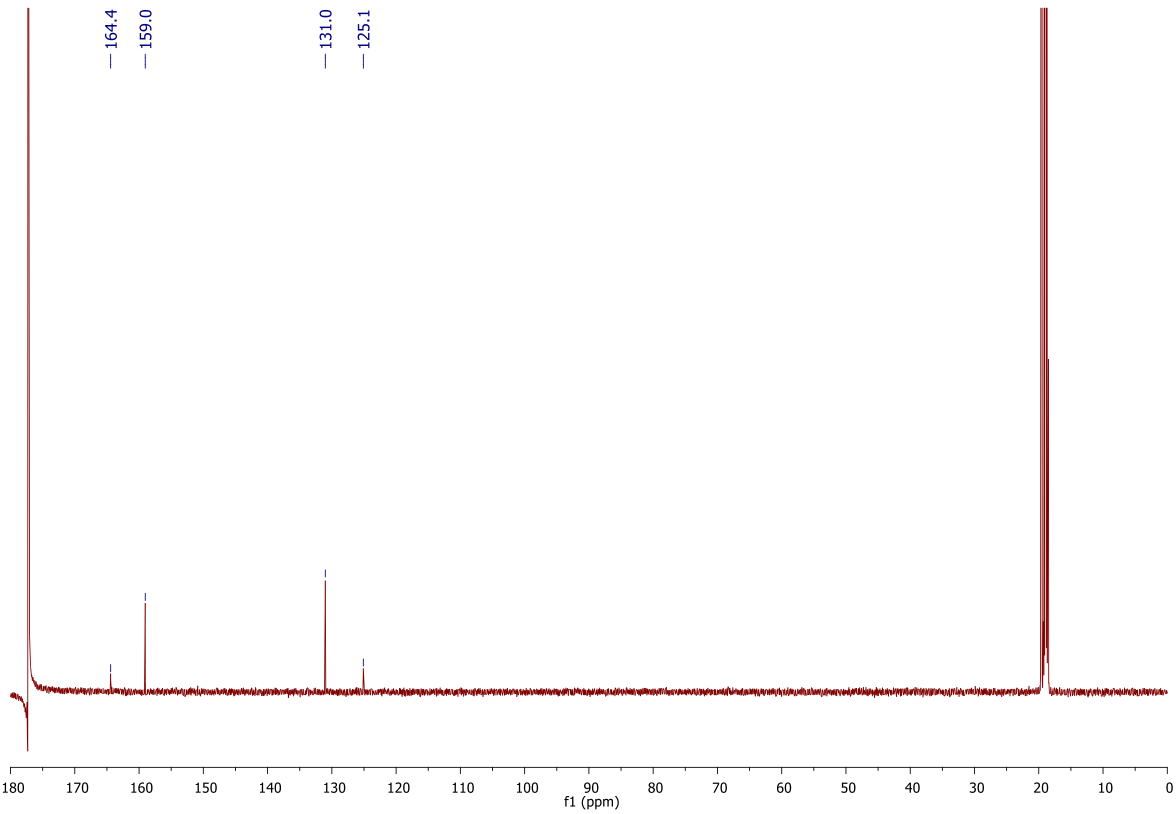

60 min:

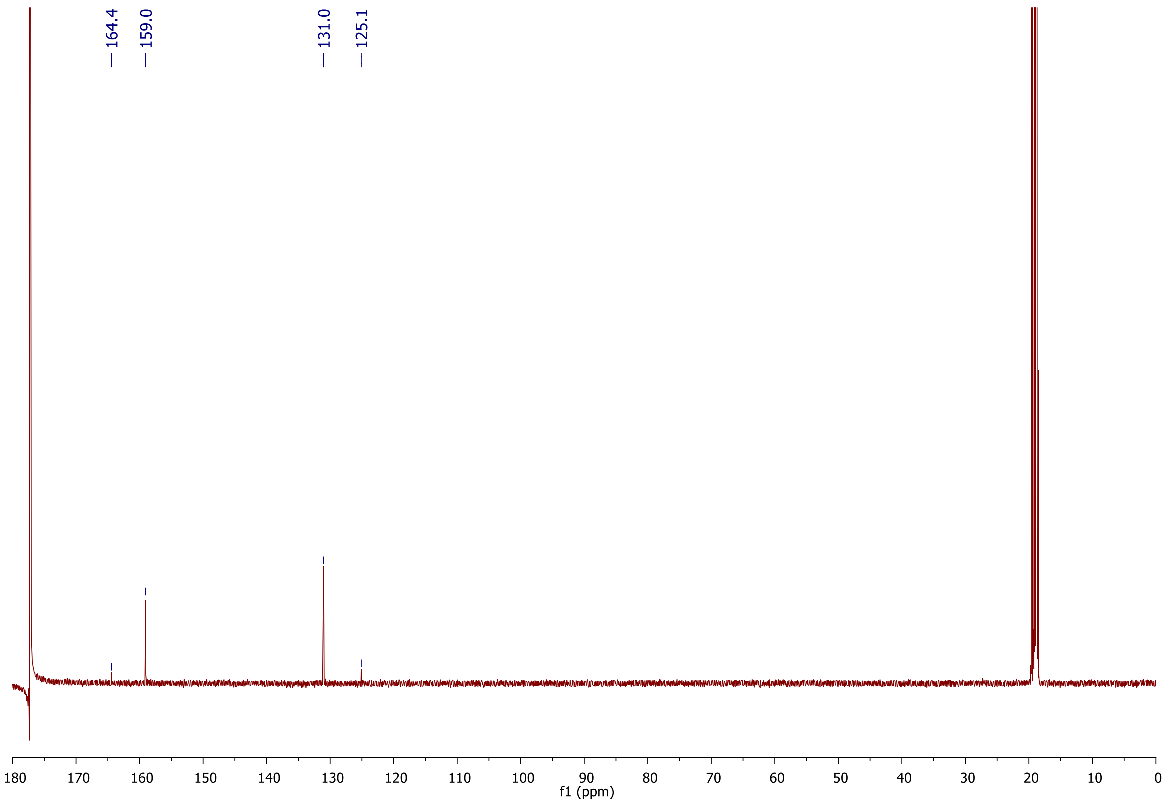

90 min:

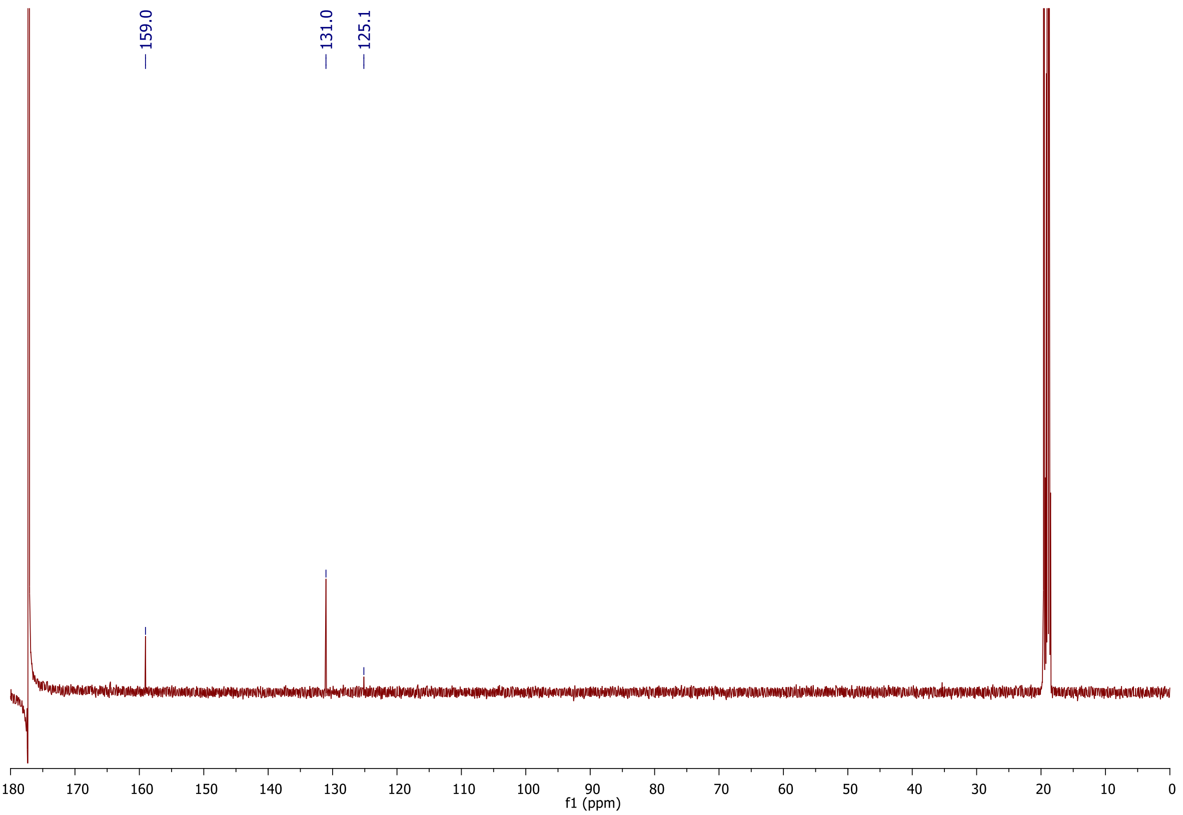

Figure S3 ^{13}C NMR data showing the formation of dibromomaleic anhydride **2**.

Tri-*tert*-butyl 2-methylhydrazine-1,1,2-tricarboxylate (S2)[2]

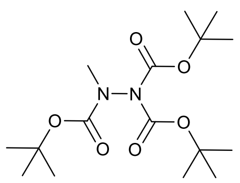

S2

To a solution of methylhydrazine **S1** (1.00 g, 1.14 mL, 21.7 mmol), NEt_3 (4.34 g, 6.04 mL, 43.4 mmol) and DMAP (260 mg, 2.17 mmol) in CH_2Cl_2 (75 mL) was added Boc_2O (18.9 g, 86.6 mmol), and the reaction mixture stirred at 20 °C for 72 h. After this time, the reaction mixture was diluted with H_2O (80 mL), extracted with EtOAc (3 × 60 mL), and the combined organic layers dried ($MgSO_4$) and concentrated *in vacuo*. The crude residue was purified by flash column chromatography (20% EtOAc/petrol) to afford tri-*tert*-butyl 2-methylhydrazine-1,1,2-tricarboxylate **S2** (7.43 g, 21.5 mmol, 99%) as a yellowish oil: ^{1}H NMR (600 MHz, $CDCl_3$) (major rotamer) δ 3.05 (s, 3H), 1.51–1.43 (m, 27H); ^{13}C NMR (150 MHz, $CDCl_3$) (major rotamer) δ 154.0 (C), 150.1 (C) 83.4 (C), 81.4 (C), 35.7 (CH_3), 28.3 (CH_3), 28.1 (CH_3); LRMS (ES+) 369 (100, $[M+Na]^+$).

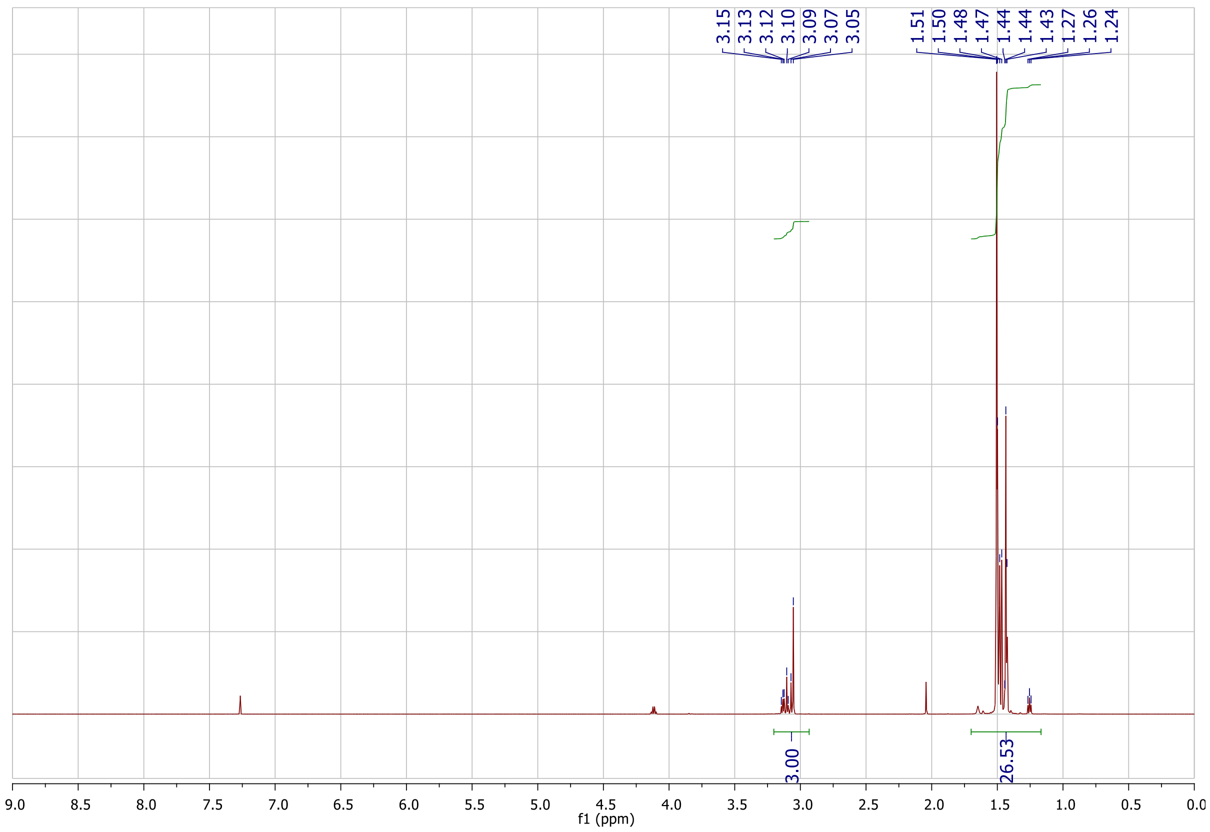

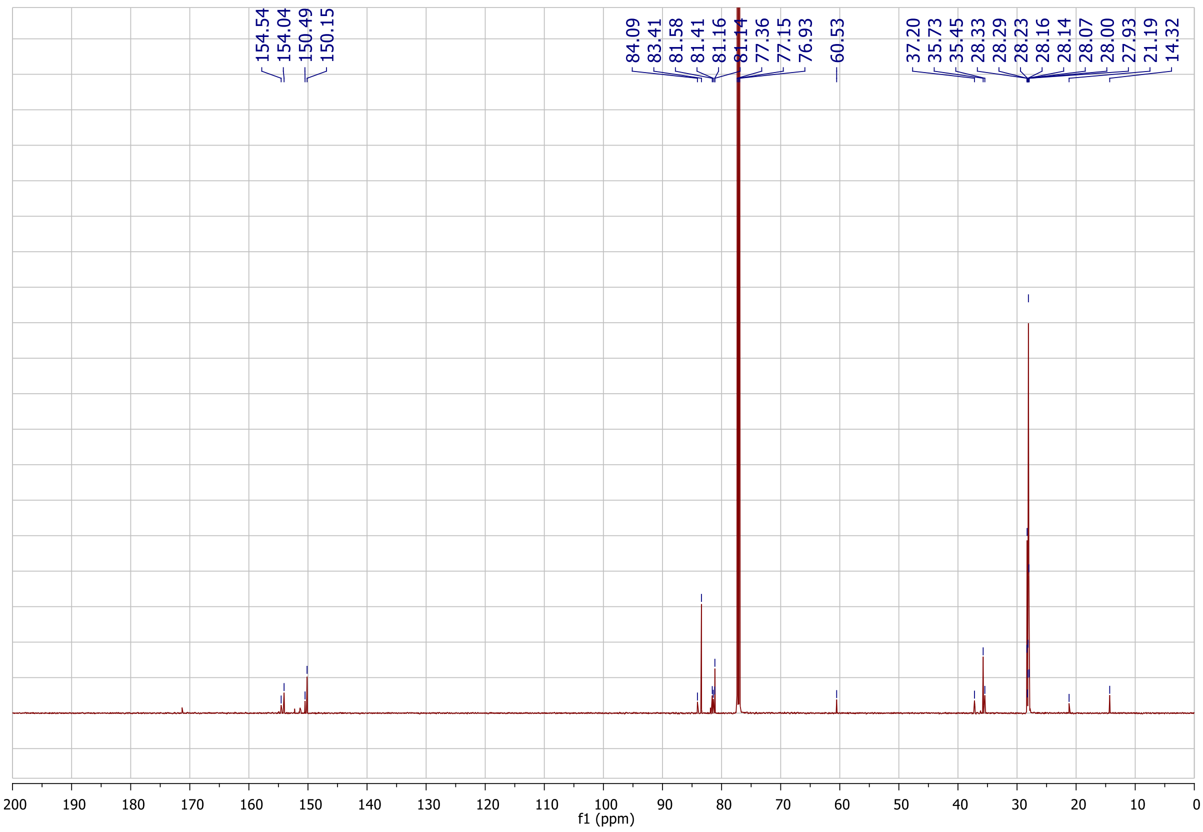

Figure **S4**. ^{1}H and ^{13}C NMR data for tri-*tert*-butyl 2-methylhydrazine-1,1,2-tricarboxylate **S2**.

Di-*tert*-butyl 1-methylhydrazine-1,2-dicarboxylate (S3)[2]

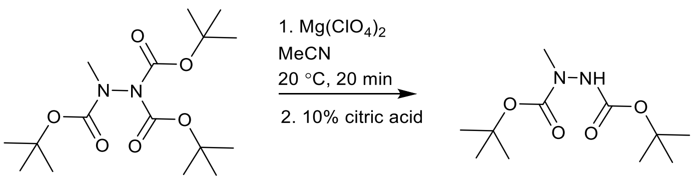

To a solution of tri-*tert*-butyl 2-methylhydrazine-1,1,2-tricarboxylate **S2** (2.0 g, 5.8 mmol) in dry MeCN (15 mL) was added $Mg(ClO_4)_2$ (0.270 g, 1.21 mmol), and the reaction mixture stirred at 20 °C for 1 h. After this time, the reaction mixture was diluted with 10% aq. citric acid (20 mL) and Et_2O (15 mL), extracted with Et_2O (3 × 20 mL), and the combined organic layers were dried ($MgSO_4$) and concentrated *in vacuo*. The crude residue was purified by flash column chromatography (15% EtOAc/petrol) to afford di-*tert*-butyl 1-methylhydrazine-1,2-dicarboxylate **S3** (1.30 g, 5.2 mmol, 89%) as a white solid: m.p. 53–56 °C; ^{1}H NMR (600 MHz, $CDCl_3$) (major rotamer) δ 6.40 (br. s, 1H), 3.11 (s, 3H), 1.48–1.45 (m, 18H); ^{13}C NMR (150 MHz, $CDCl_3$) (major rotamer) δ 155.9 (C), 155.3 (C) 81.3 (C), 81.1 (C), 37.6 (CH_3), 28.3 (CH_3), 28.1 (CH_3); IR (solid) 3316, 2978, 2932, 1701 cm^{-1}; LRMS (ES+) 269 (100, $[M+Na]^+$); HRMS (ES+) calcd. for $C_{11}H_{22}N_2O_4Na$ $[M+Na]^+$ 269.1477, observed 269.1476.

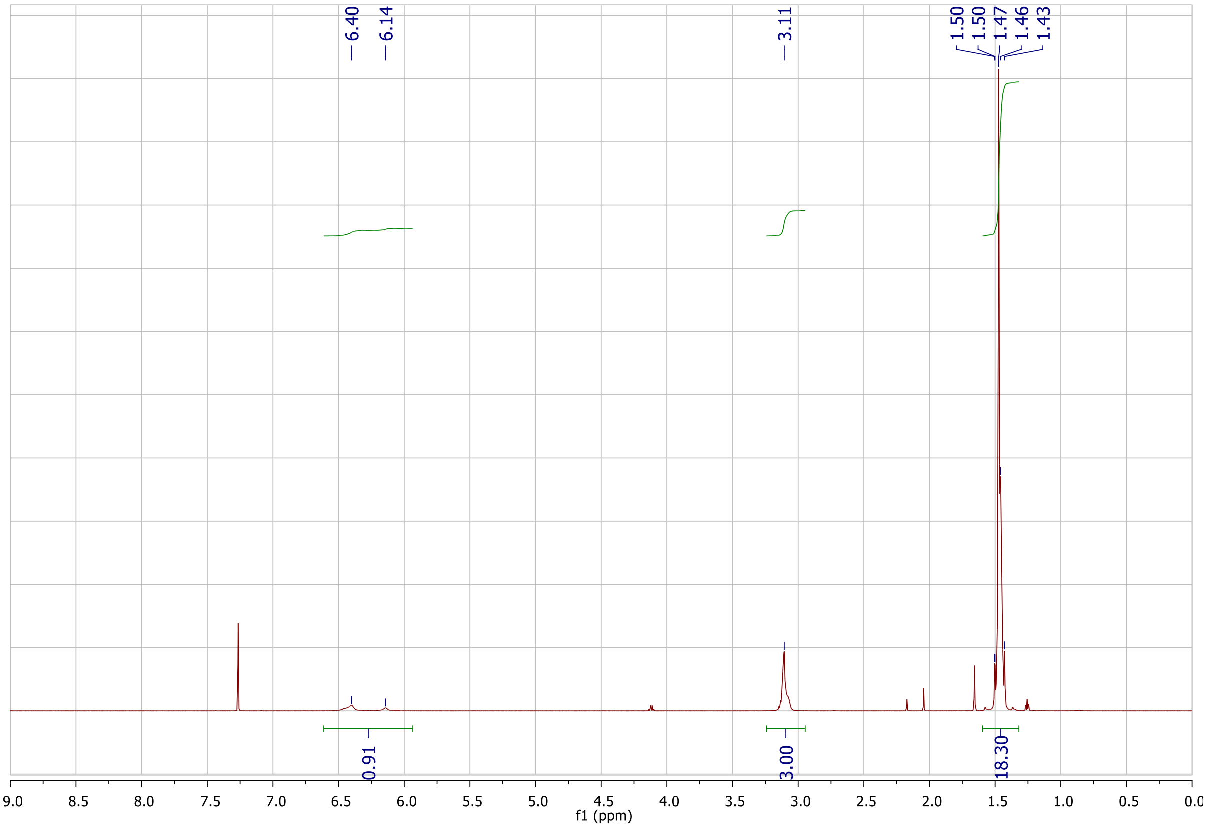

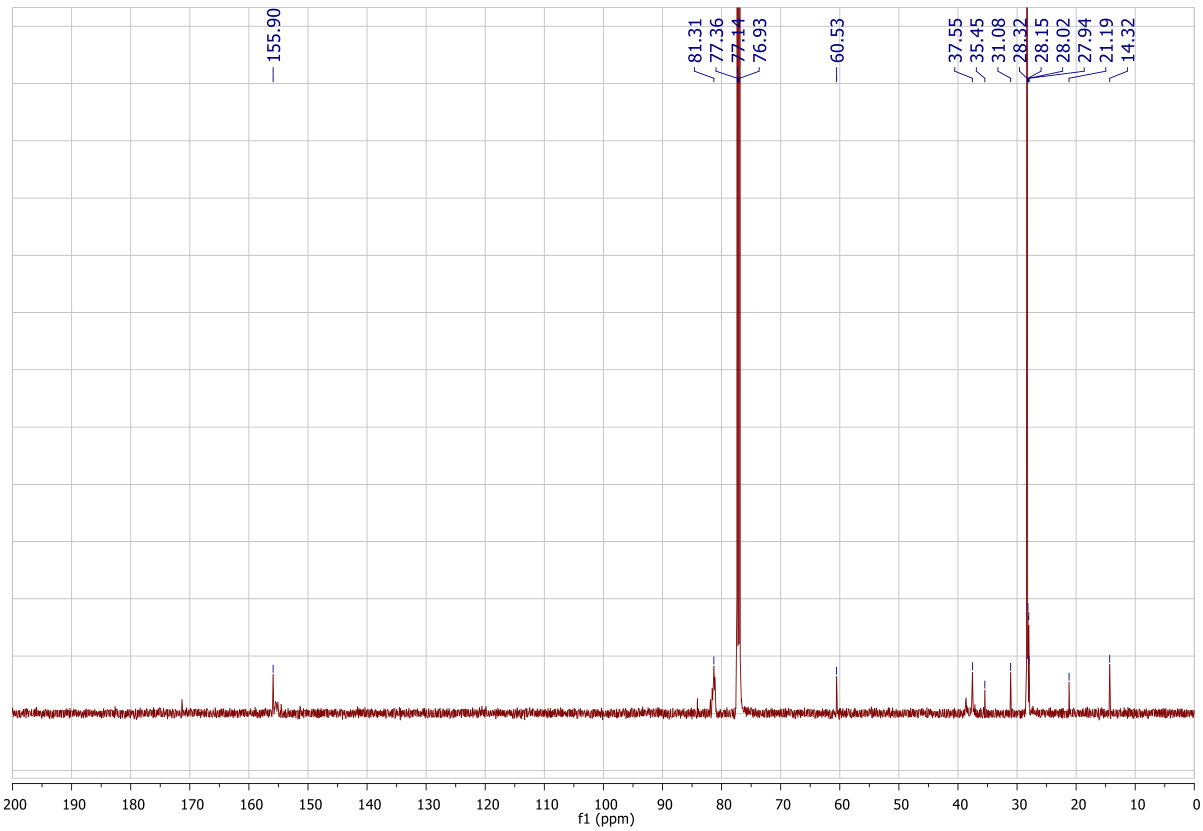

Figure **S5**. ^{1}H and ^{13}C NMR data for di-*tert*-butyl 1-methylhydrazine-1,2-dicarboxylate **S3**.

Di-*tert*-butyl 1-(2-(*tert*-butoxy)-2-oxoethyl)-2-methylhydrazine-1,2-dicarboxylate (S4)[2]

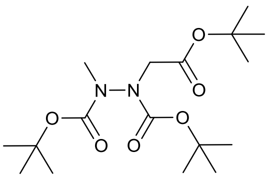

S4

To a solution of di-*tert*-butyl 1-methylhydrazine-1,2-dicarboxylate **S3** (0.940 g, 3.80 mmol) in DMF (20 mL) was added caesium carbonate (1.86 g, 5.70 mmol) and then *tert*-butyl bromoacetate (1.10 g, 0.840 mL, 5.70 mmol), and the reaction mixture stirred at 20 °C for 16 h. After this time, the reaction mixture was diluted with H_2O (50 mL), extracted with Et_2O (4 × 50 mL), the combined organic layers washed with sat. aq. LiCl (2 × 30 mL), dried ($MgSO_4$), and concentrated *in vacuo*. Purification by flash column chromatography (10% Et_2O/petrol) yielded di-*tert*-butyl 1-(2-(*tert*-butoxy)-2-oxoethyl)-2-methylhydrazine-1,2-dicarboxylate **S4** (1.30 g, 3.70 mmol, 98%) as a colourless oil: ^1H NMR (600 MHz, $CDCl_3$) δ 4.73–4.04 (m, 2H), 3.68–3.10 (m, 3H), 1.54–1.39 (m, 27H); ^{13}C NMR (150 MHz, $CDCl_3$) (major rotamer) δ 169.2 (C), 155.2 (C), 81.9 (C), 81.6 (C), 81.1 (C), 52.7 (CH_2), 36.8 (CH_3), 28.4 (CH_3), 28.3 (CH_3), 28.2 (CH_3); IR (thin film) 2978, 1748 cm^{-1}; LRMS (ES+) 361 (100, $[M+H]^+$); HRMS (ES+) calcd for $C_{17}H_{33}O_6N_2$ $[M+H]^+$ 361.2339, observed 361.2333.

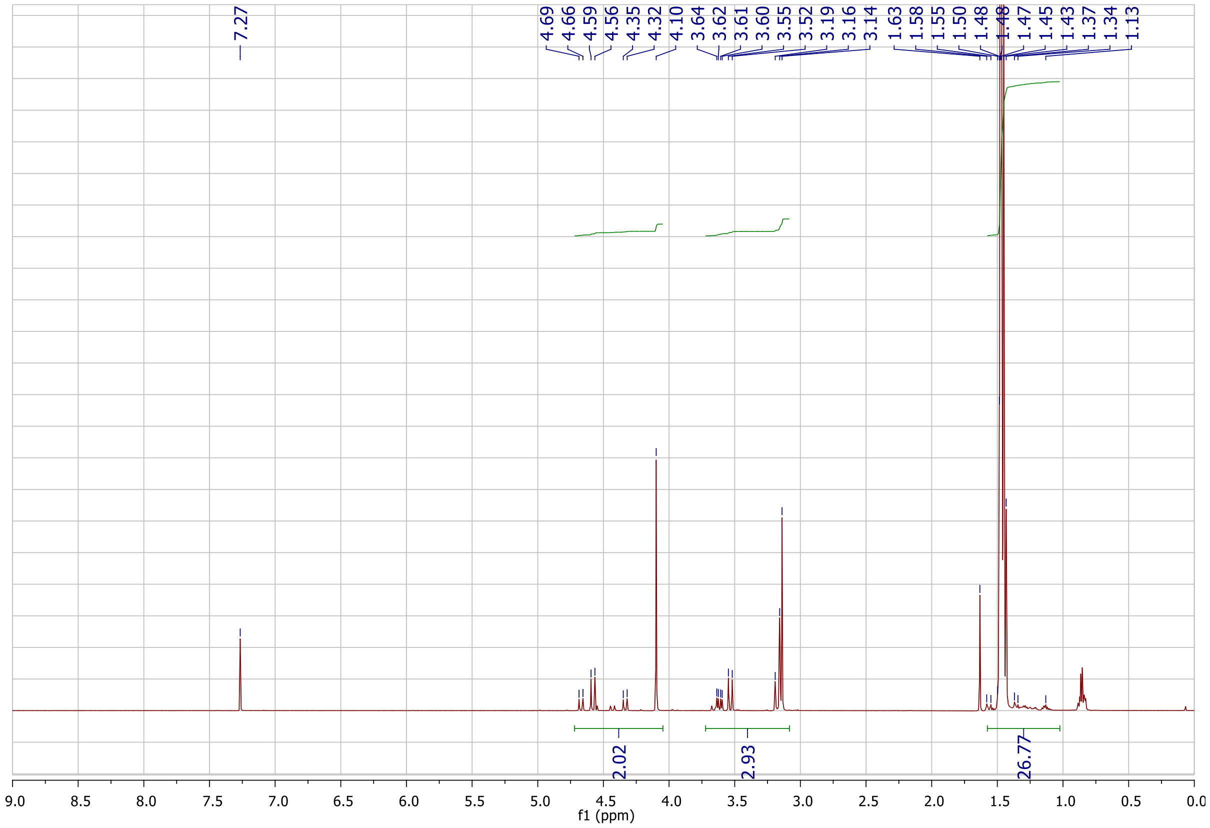

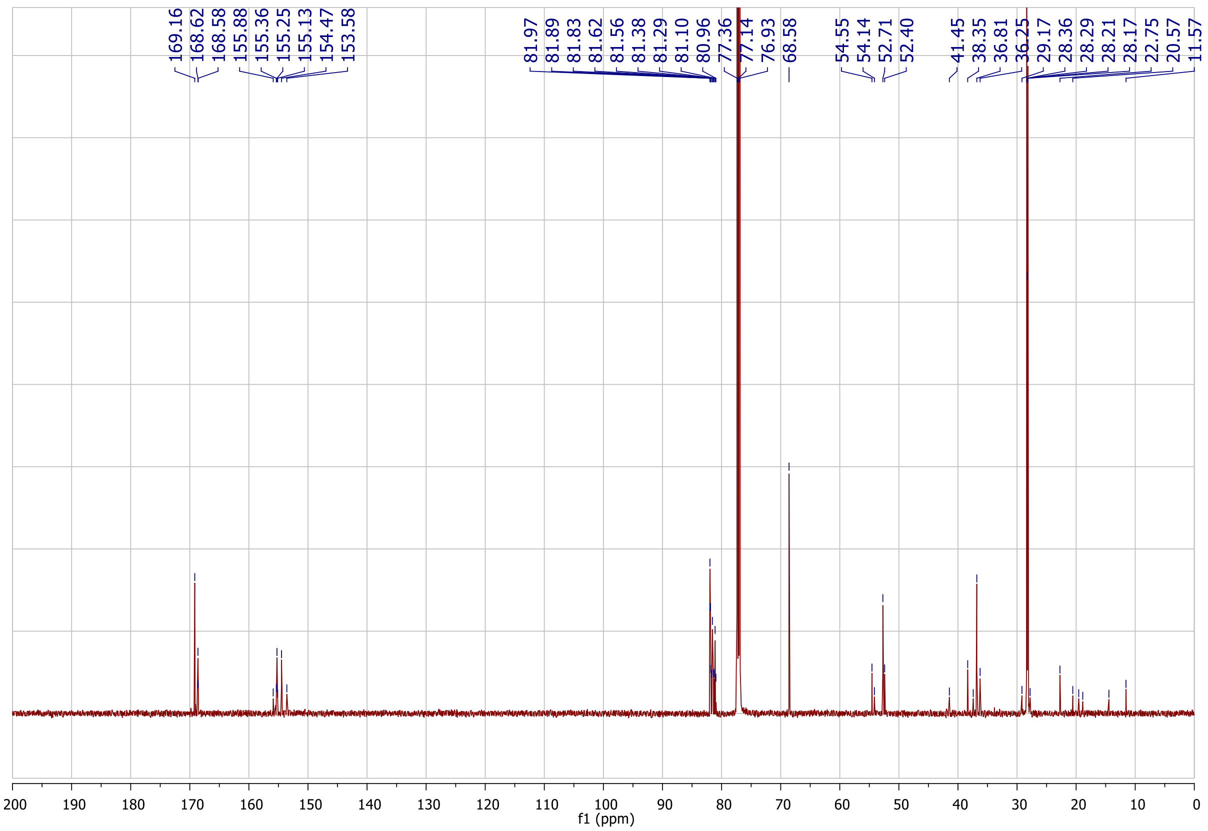

Figure **S6**. ^{1}H and ^{13}C NMR data for di-*tert*-butyl 1-(2-(*tert*-butoxy)-2-oxoethyl)-2-methylhydrazine-1,2-dicarboxylate **S4**.

2-(4,5-Dibromo-2-methyl-3,6-dioxo-3,6-dihydropyridazin-1(2*H*)-yl)acetic acid (13)[2]

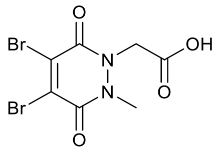

13

To a solution of di-*tert*-butyl 1-(2-(*tert*-butoxy)-2-oxoethyl)-2-methylhydrazine-1,2-dicarboxylate **S4** (1.00 g, 2.80 mmol) in CH_2Cl_2 (10 mL) was added TFA (10 mL), and the reaction mixture stirred at 20 °C for 2 h. After this time, all volatile materials were removed *in vacuo*. The crude residue was added to a solution of 2,3-dibromomaleic anhydride (0.750 g, 2.80 mmol) in glacial AcOH (40 mL), and the reaction mixture stirred at 20 °C for 16 h before raising the temperature to 130 °C for 16 h. After this time, the reaction mixture was concentrated *in vacuo*, and purification of the crude residue by flash column chromatography (3% MeOH/CH_2Cl_2 with 1% AcOH) yielded 2-(4,5-dibromo-2-methyl-3,6-dioxo-3,6-dihydropyridazin-1(2*H*)-yl)acetic acid **13** (0.650 g, 1.90 mmol, 73%) as a white solid: m.p. 210–214 °C; ^{1}H NMR (600 MHz, MeOD) δ 4.96 (s, 2H), 3.62 (s, 3H); ^{13}C NMR (150 MHz, MeOD) δ 170.2 (C), 154.8 (C), 154.0 (C), 137.4 (C), 135.7 (C), 49.5 (CH_2), 35.0 (CH_3); IR (solid) 3023, 2969, 1731, 1662 cm^{-1}; LRMS (ES−) 341 (50, $[M^{81}Br^{81}Br\text{-}H]^-$), 339 (100, $[M^{81}Br^{79}Br\text{-}H]^-$), 337 (50, $[M^{79}Br^{79}Br\text{-}H]^-$); HRMS (ES−) calcd for $C_7H_5N_2O_4Br_2$ $[M^{79}Br^{79}Br\text{-}H]^-$ 336.8538, observed 336.8540.

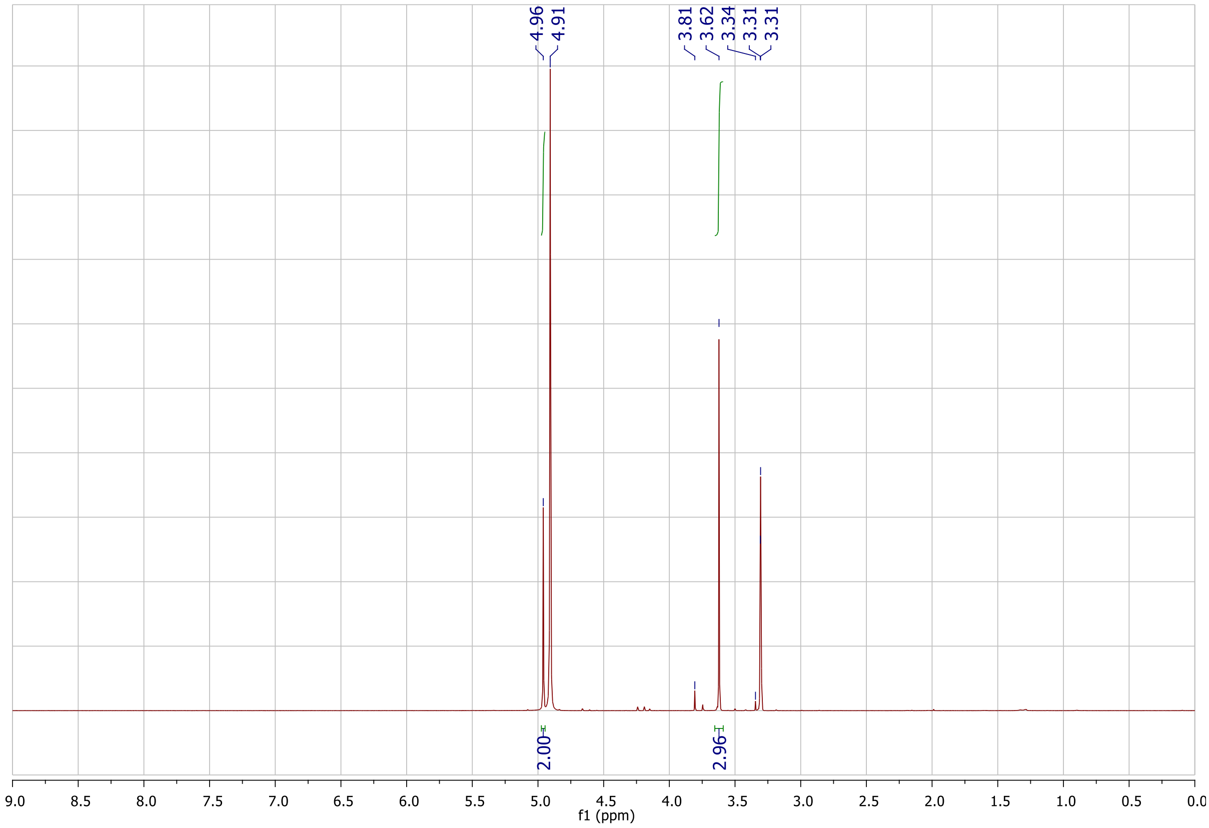

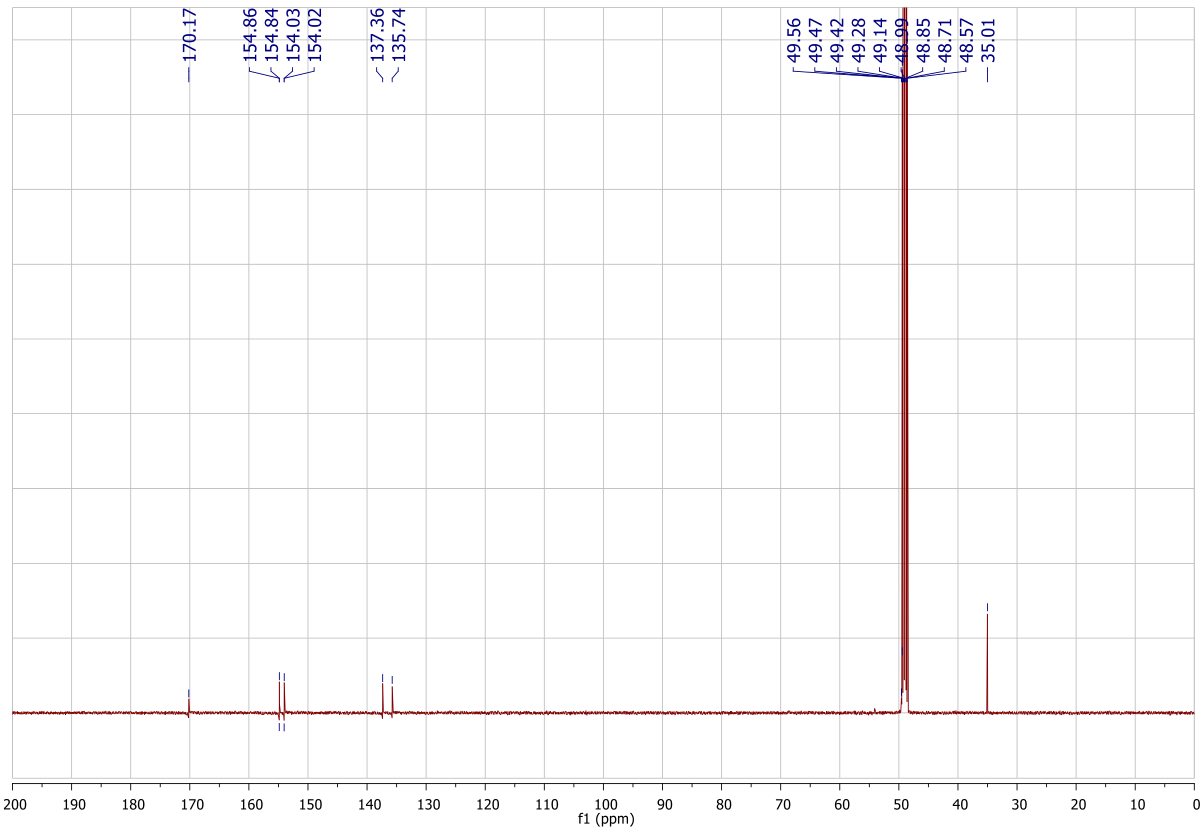

Figure **S7**. ^{1}H and ^{13}C NMR data for 2-(4,5-dibromo-2-methyl-3,6-dioxo-3,6-dihydropyridazin-1(2*H*)-yl)acetic acid **13**.

Di-*tert*-butyl-1-methylhydrazine-1,2-dicarboxylate (S3)[2]

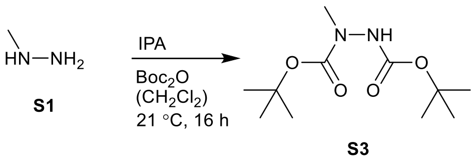

To a solution of methyl hydrazine **S1** (1.14 mL, 21.7 mmol) in *i*-PrOH (16 mL), was added a solution of di-*tert*-butyl dicarbonate (11.4 g, 52.1 mmol, pre-dissolved in CH_2Cl_2 (12 mL)) drop-wise over 30 min. The reaction was then stirred at 21 °C for 16 h. After this time, the solvents were then removed *in vacuo* and the crude residue purified by flash column chromatography (0% to 15% EtOAc/petrol) to afford di-*tert*-butyl-1-methylhydrazine-1,2-dicarboxylate **S3** (4.67 g, 19.1 mmol, 88%) as a white solid: m.p. 58–62 °C (*lit m.p.* 54–56 °C)[1]; ^1H NMR (600 MHz, $CDCl_3$, rotamers) δ 6.41–6.16 (m, 1H) 3.11 (s, 3H), 1.47–1.46 (m, 18H); ^{13}C NMR (150 MHz, $CDCl_3$, rotamers) δ 155.9 (C), 81.3 (C), 37.5 (CH_3), 28.3 (CH_3); IR (solid) 3315, 2981, 1702 cm^{-1}.

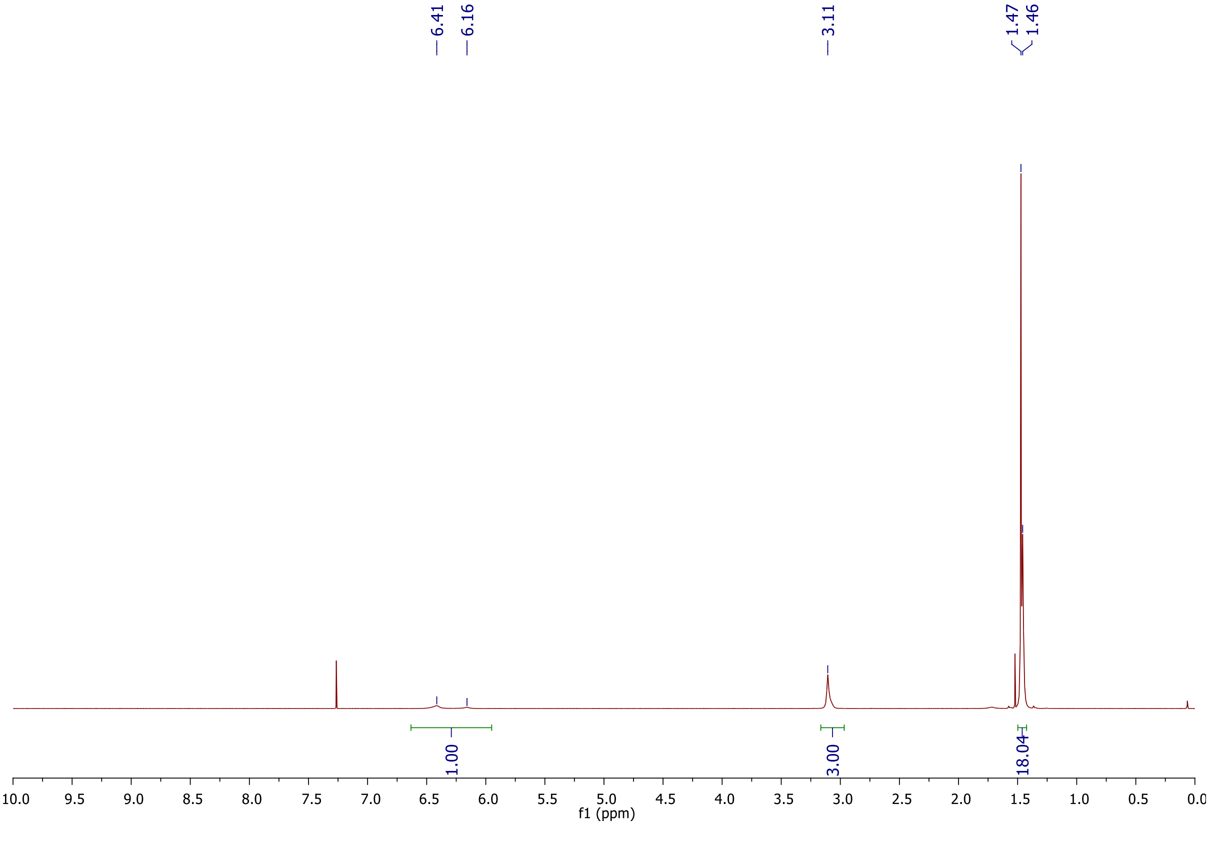

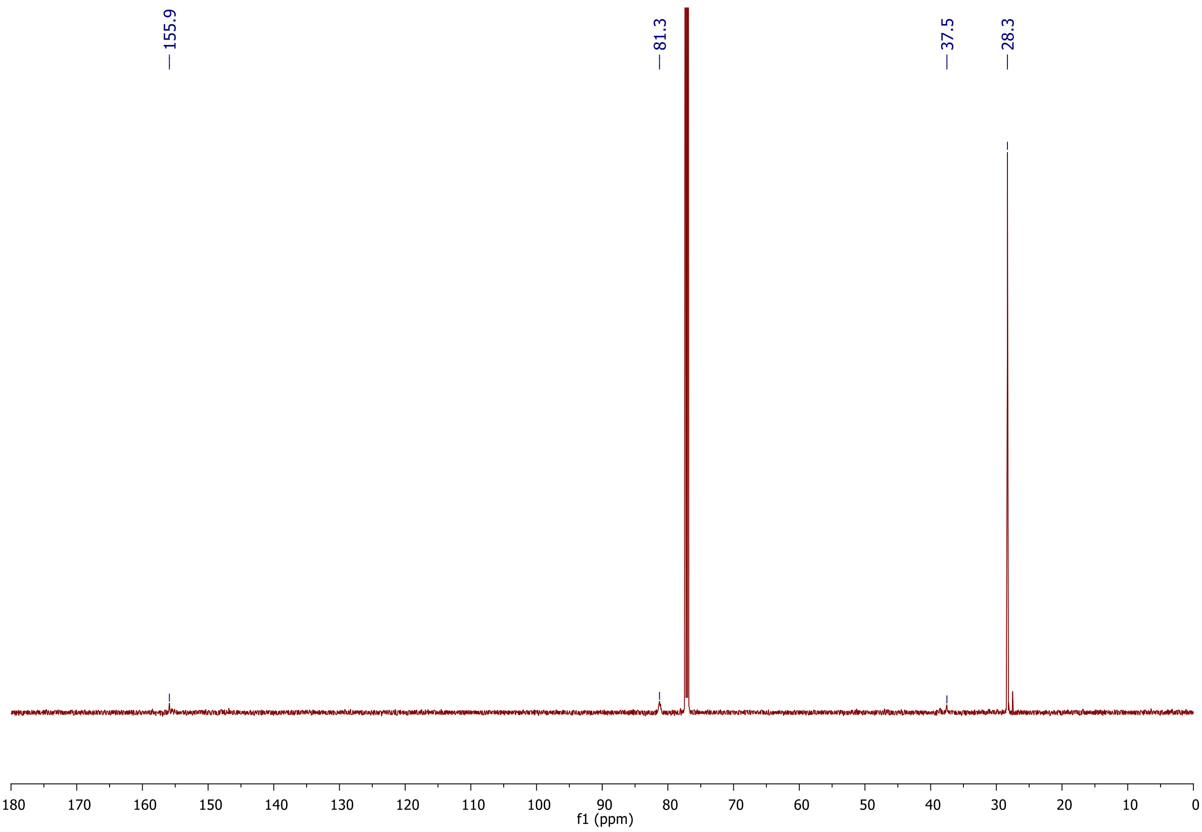

Figure S8 ^{1}H and ^{13}C NMR data for di-*tert*-butyl-1-methylhydrazine-1,2-dicarboxylate **S3**.

Di-*tert*-butyl-1-(3-(*tert*-butoxy)-3-oxopropyl)-2-methylhydrazine-1,2-dicarboxylate (S6)

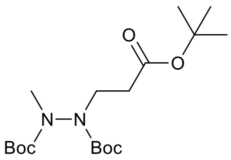

S6

To a solution of di-*tert*-butyl 1-methylhydrazine-1,2-dicarboxylate **S3** (3.00 g, 12.2 mmol) in *t*-BuOH (15 mL) was added 10% NaOH (0.5 mL) and the reaction mixture stirred at 21 °C for 10 min. After this, *tert*-butyl acrylate (5.31 mL, 36.6 mmol) was added to the solution and the reaction mixture was heated at 60 °C for 24 h. Following this, the solvent was removed *in vacuo* and the crude residue was dissolved in EtOAc (150 mL) and washed with water (3 × 50 mL). The organic layer was then dried ($MgSO_4$) and concentrated *in vacuo.* Purification of the crude residue by flash column chromatography (0% to 20% EtOAc/petrol) to afford di-*tert*-butyl-1-(3-(*tert*-butoxy)-3-oxopropyl)-2-methylhydrazine-1,2-dicarboxylate **S6** (3.33 g, 8.91 mmol, 73%) as a clear oil: ^{1}H NMR (600 MHz, $CDCl_3$, rotamers) δ 3.85–3.52 (m, 2H), 3.06–2.99 (m, 3H), 2.51 (t, *J* = 7.2 Hz, 2H), 1.48–1.43 (m, 27H); ^{13}C NMR (150 MHz, $CDCl_3$, rotamers) δ 171.0 (C), 155.4 (C), 154.4 (C), 81.0 (C), 44.6 (CH_3), 36.6 (CH_2), 34.1 (CH_2), 28.3 (CH_3); IR (thin film) 2976, 2933, 1709 cm^{-1}; LRMS (ESI) 375 (100, $[M+H]^+$), 319 (30, $[M-C_4H_9+2H]^+$ HRMS (ESI) calcd for $C_{18}H_{35}N_2O_6$ $[M+H]^+$ 376.2524; observed 376.2516.

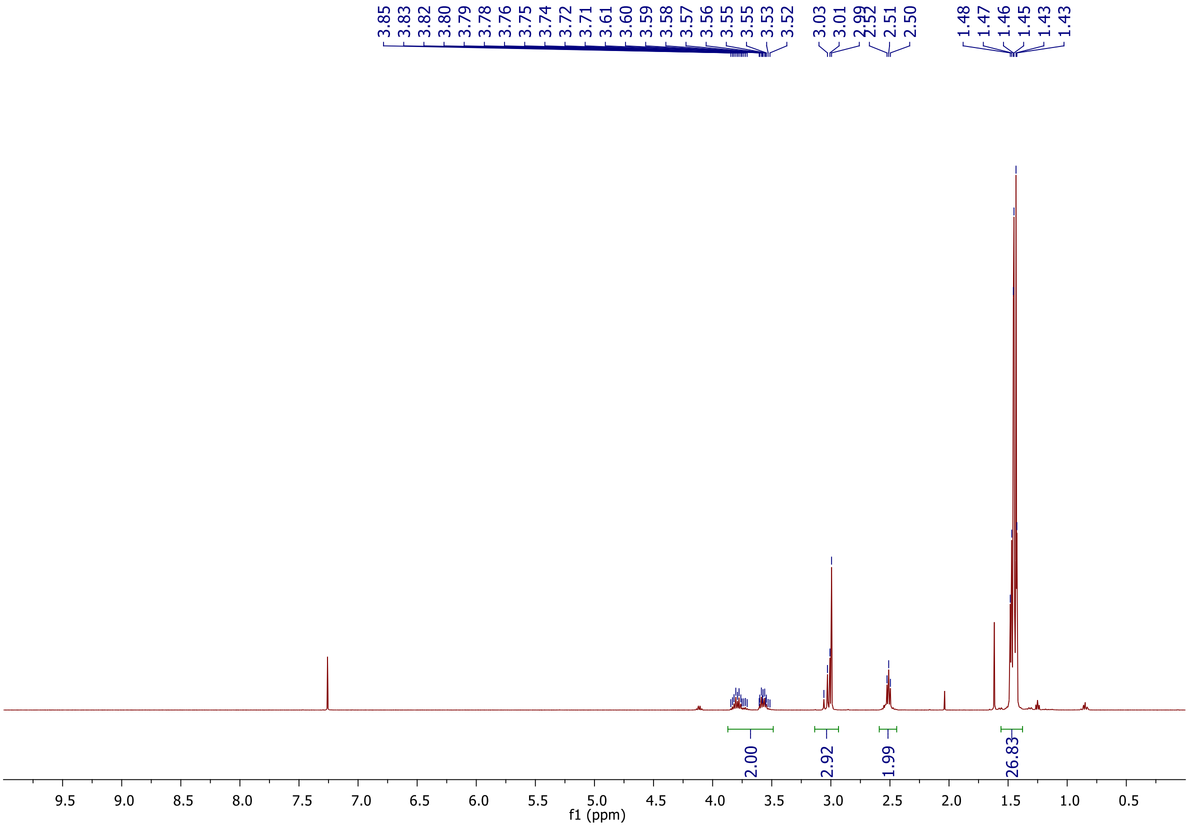

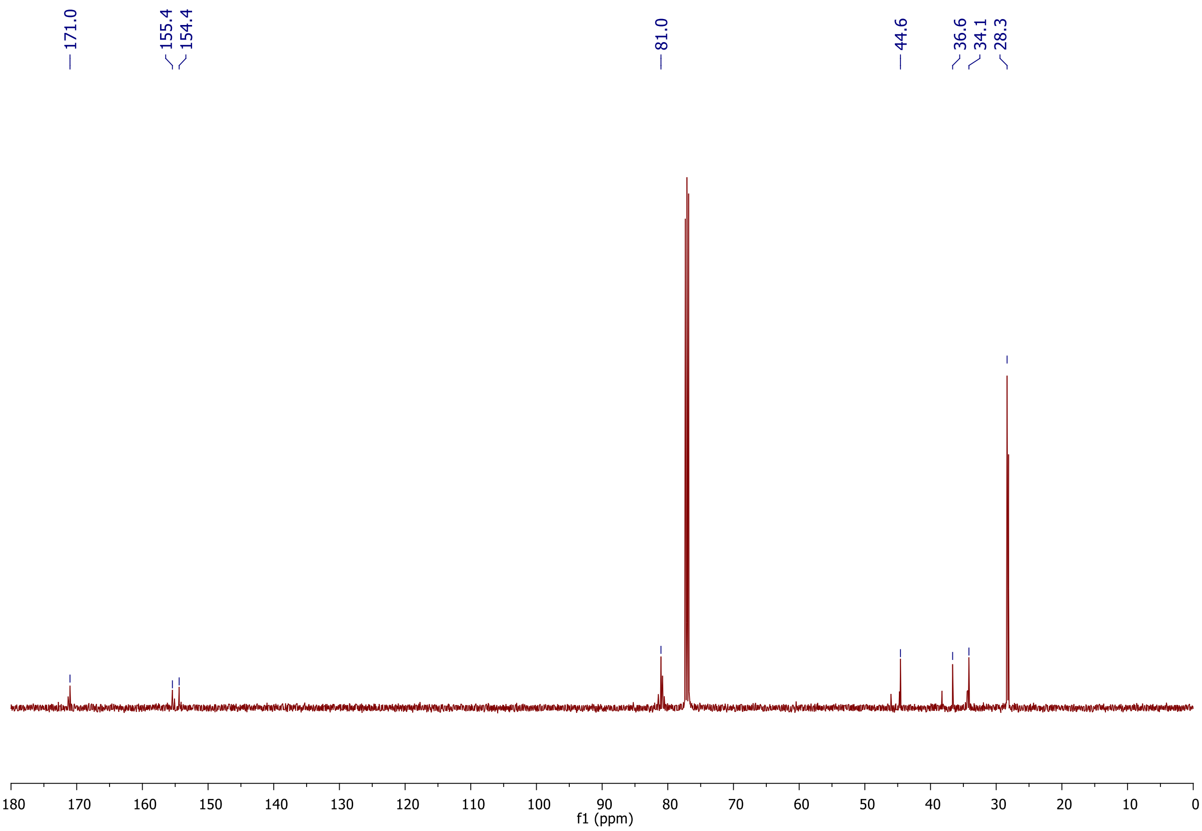

Figure S9 ^{1}H and ^{13}C NMR data for di-*tert*-butyl-1-(3-(*tert*-butoxy)-3-oxopropyl)-2-methylhydrazine-1,2-dicarboxylate **S6**.

3-(4,5-Dibromo-2-methyl-3,6-dioxo-3,6-dihydropyridazin-1(2*H*)-yl) propanoic acid (15)

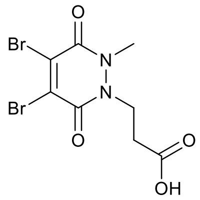

15

Dibromomaleic acid **6** (880 mg, 3.06 mmol) was dissolved in AcOH (25 mL) and heated under reflux for 30 min. To this solution, was added di-*tert*-butyl-1-(3-(*tert*-butoxy)-3-oxopropyl)-2-methylhydrazine-1,2-dicarboxylate **S6** (1.00 g, 2.67 mmol) and the reaction heated under reflux for a further 4 h. After this time, the reaction mixture was concentrated *in vacuo* with toluene co-evaporation (3 × 30 mL, as an azeotrope) and the crude residue purified by flash column chromatography (50% to 100% EtOAc/petrol (1% AcOH)) to afford 3-(4,5-dibromo-2-methyl-3,6-dioxo-3,6-dihydropyridazin-1(2*H*)-yl) propanoic acid **15** (801 mg, 2.25 mmol, 84%) as a yellow solid: m.p. 140–144 °C; ^{1}H NMR (600 MHz, DMSO-d$_6$) δ 4.28 (t, *J* = 7.3 Hz, 2H), 3.56 (s, 3H), 2.63 (t, *J* = 7.3 Hz, 2H); ^{13}C NMR (150 MHz, DMSO-d$_6$) δ 171.9 (C), 152.7 (C), 152.4 (C), 135.3 (C), 135.0 (C), 43.1 (CH_3), 34.7 (CH_2), 31.7 (CH_2); IR (solid) 3044, 1725, 1606, 1570 cm^{-1} LRMS (ESI). 359 (50, [M^{81}Br^{81}Br+H]$^+$) 357 (100, [M^{79}Br^{81}Br+H]$^+$), 355 (50, [M^{79}Br^{79}Br+H]$^+$). HRMS (ESI) calcd for C_8H_9-$Br_2N_2O_4$ [M^{79}Br^{81}Br+H]$^+$ 358.8883; observed 358.8882.

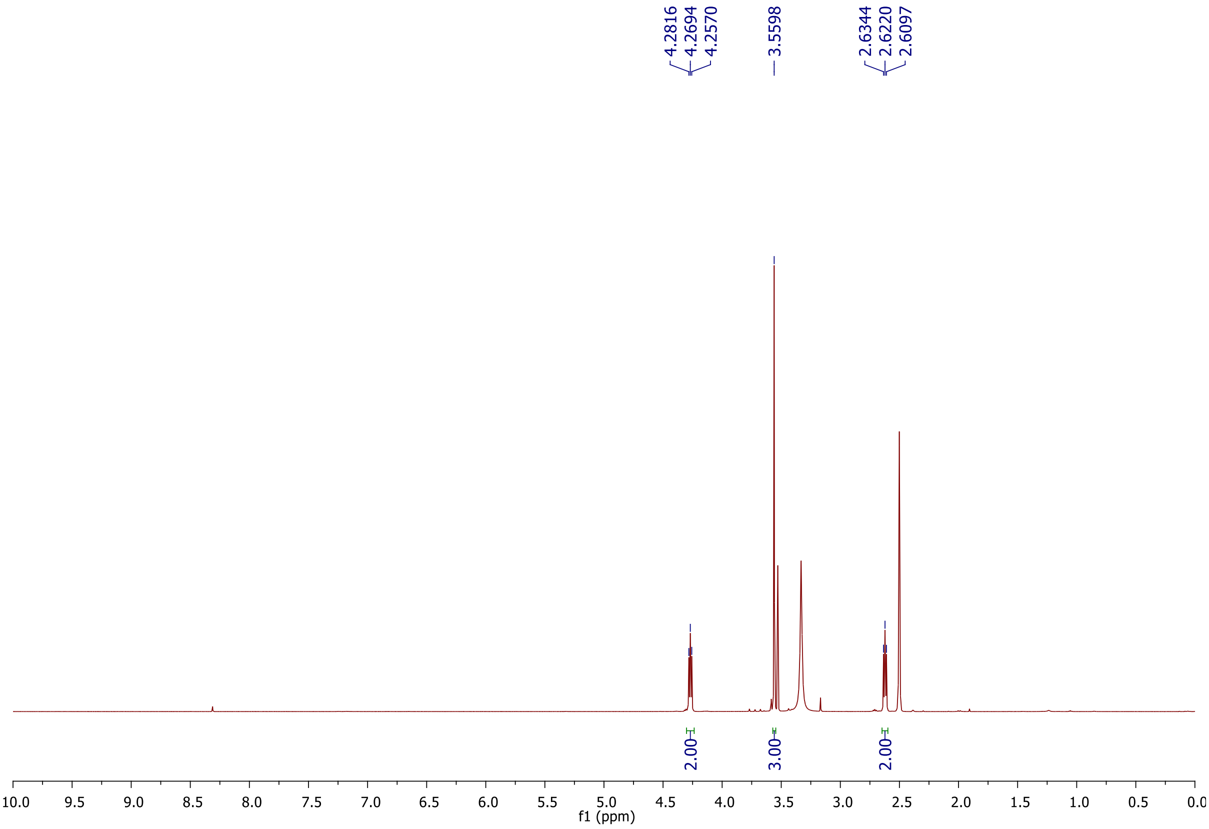

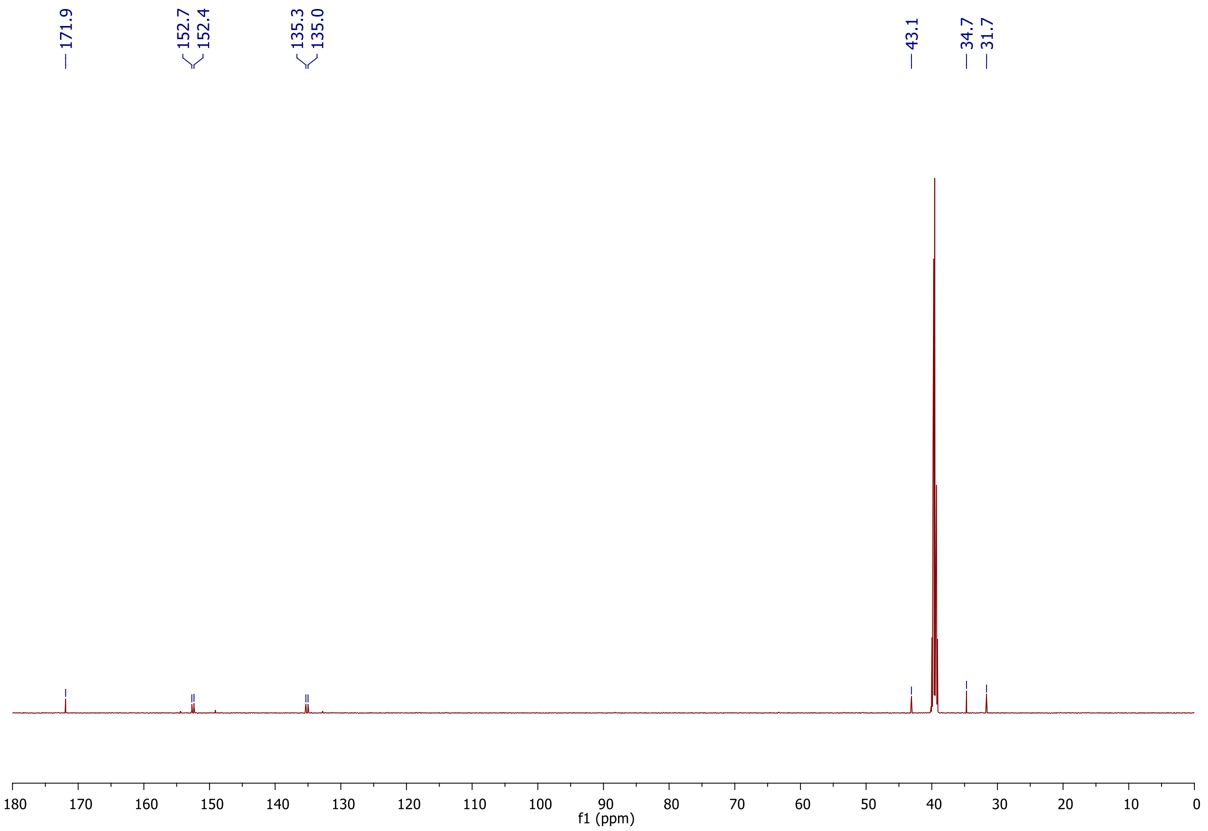

Figure S10 ^{1}H and ^{13}C NMR data for 3-(4,5-dibromo-2-methyl-3,6-dioxo-3,6-dihydropyridazin-1(2*H*)-yl) propanoic acid **15**.

2,5-Dioxopyrrolidin-1-yl 3-(4,5-dibromo-2-methyl-3,6-dioxo-3,6-dihydropyridazin-1(2*H*)-yl) propanoate (16)

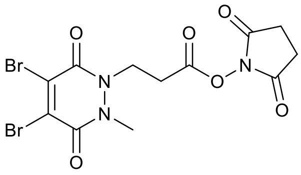

16

To a solution of 3-(4,5-dibromo-2-methyl-3,6-dioxo-3,6-dihydropyridazin-1(2*H*)-yl) propanoic acid **15** (250 mg, 0.702mmol) in THF (10 mL) cooled to 0 °C, was added *N,N'*-dicyclohexylcarbodiimide (160 mg, 0.774mmol). The homogenous solution was then stirred at 0 °C for 30 min. After this time, was added *N*-hydroxysuccinimide (89.0 mg, 0.78 mmol) and the reaction stirred at 21 °C for a further 16 h. The newly formed heterogeneous mixture was then filtered and the filtrate concentrated *in vacuo*. Purification of the crude residue by flash column chromatography (20% to 100% EtOAc/petrol) afforded 2,5-dioxopyrrolidin-1-yl 3-(4,5-dibromo-2-methyl-3,6-dioxo-3,6-dihydropyridazin-1(2*H*)-yl) propanoate **16** (230 mg, 0.507 mmol, 72%) as a yellow solid: m.p. 100–104 °C; ^{1}H NMR (600 MHz, $CDCl_3$) δ 4.48 (t, *J* = 6.9 Hz, 2H), 3.68 (s, 3H), 3.11 (t, *J* = 6.9 Hz, 2H), 2.85 (s, 4H); ^{13}C NMR (150 MHz, $CDCl_3$) δ 168.7 (C), 166.0 (C), 153.3 (C), 153.1 (C), 136.9 (C), 135.3 (C), 43.0 (CH_2), 35.3 (CH_3), 29.1 (CH_2), 25.7 (CH_2); IR (solid) 2992, 1814, 1782, 1735, 1634, 1576 cm^{-1}.

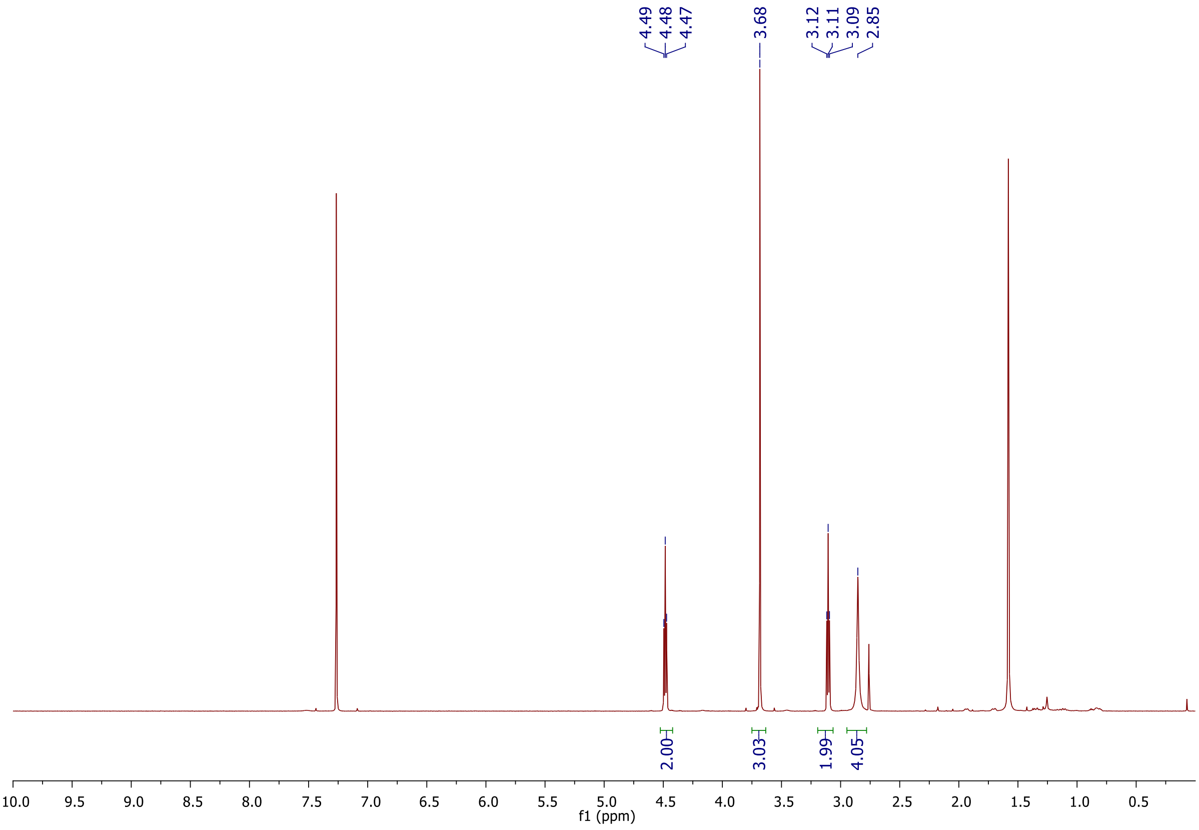

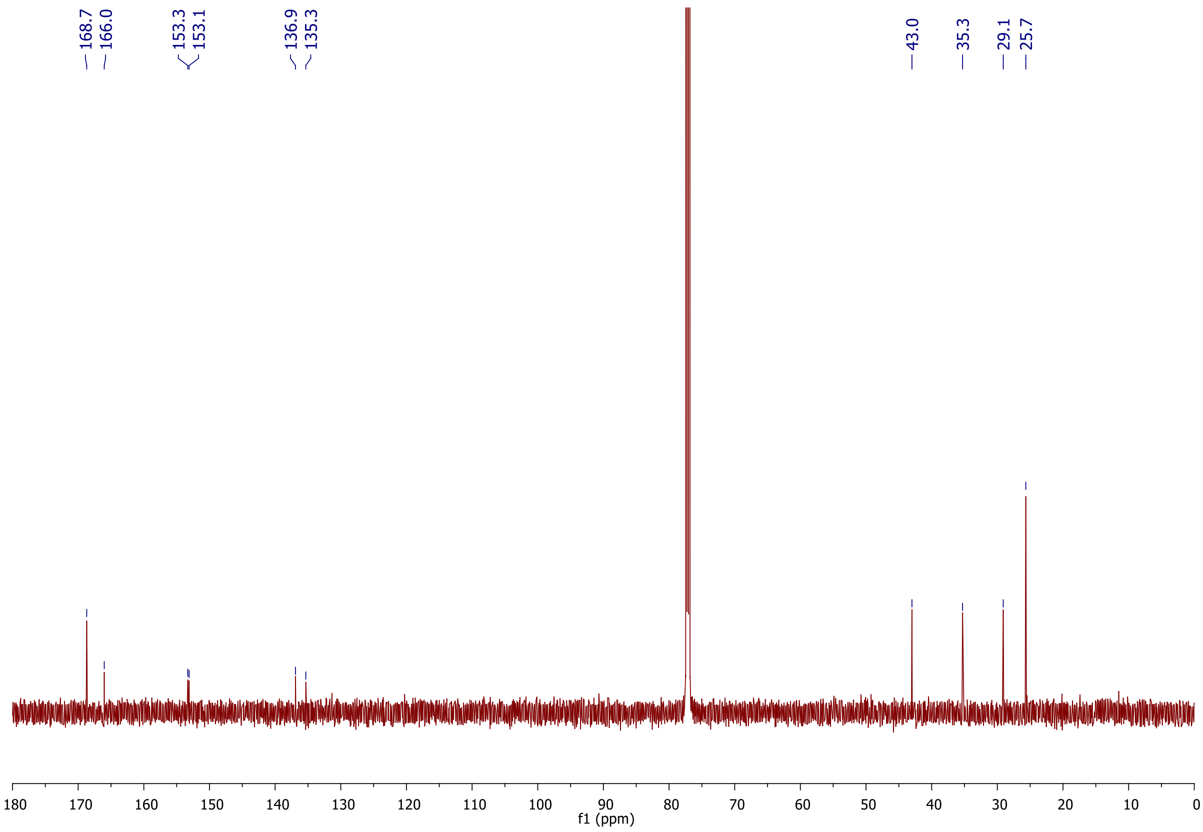

Figure S11 ^{1}H and ^{13}C NMR data for 2,5-dioxopyrrolidin-1-yl 3-(4,5-dibromo-2-methyl-3,6-dioxo-3,6-dihydropyridazin-1(2*H*)-yl) propanoate **16**.

3 months:

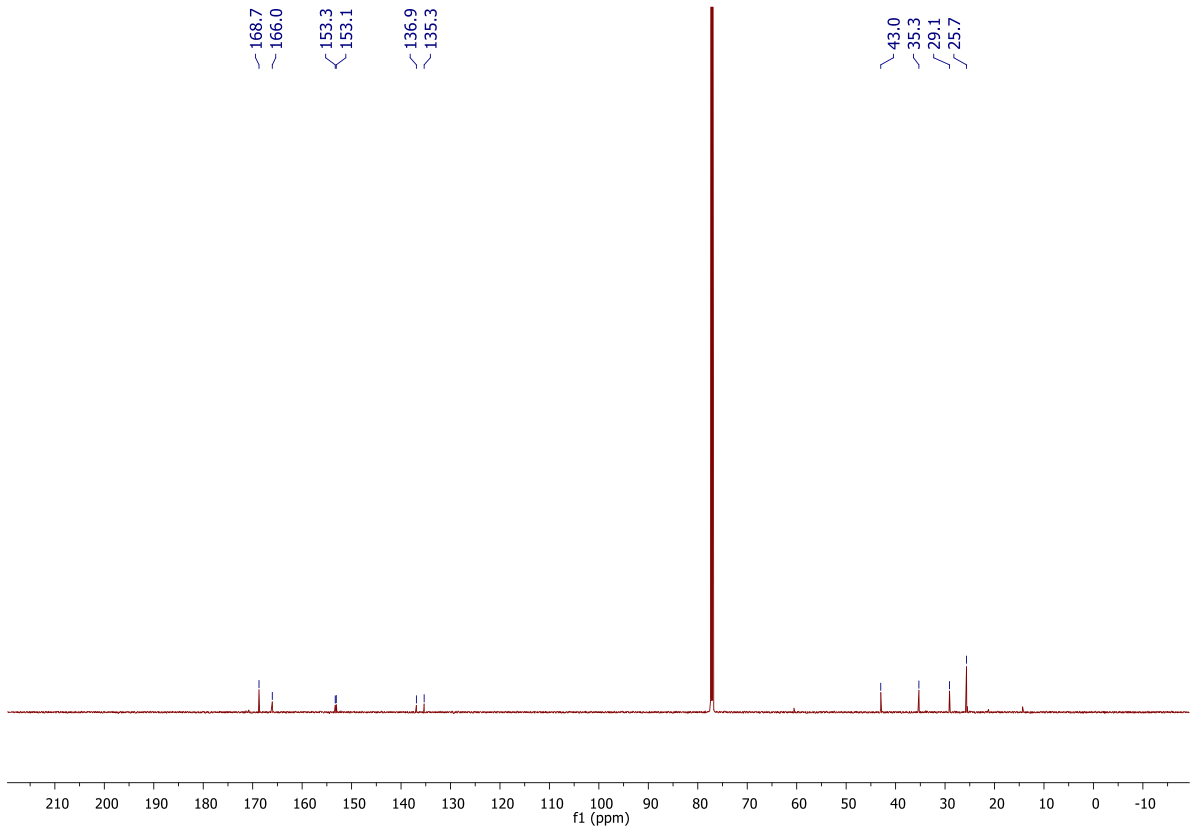

Figure S12 ^{13}C NMR data for 2,5-dioxopyrrolidin-1-yl 3-(4,5-dibromo-2-methyl-3,6-dioxo-3,6-dihydropyridazin-1(2*H*)-yl) propanoate **16** 3 months after synthesis when stored at -20 °C.

***tert*-Butyl (3-(2-(2-(3-aminopropoxy)ethoxy)ethoxy)propyl)carbamate (S6)**[3]

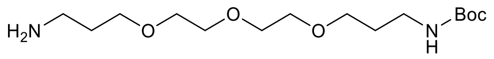

S6

To a solution of 3,3'-((oxybis(ethane-2,1-diyl))bis(oxy))bis(propan-1-amine) (8.10 g, 37.1 mmol) in 1,4-dioxane (60 mL) was added dropwise di-*tert*-butyl dicarbonate (1.00 g, 4.60 mmol, pre-dissolved in 1,4-dioxane (25 mL)) over 2 h, ensuring that the temperature did not exceed 21 °C. After this time, the reaction mixture was stirred at 21 °C for a further 30 mins. Following this, the reaction mixture was concentrated *in vacuo*, the crude residue dissolved in water (50 mL), and the organics extracted into EtOAc (5 × 30 mL). The organics were combined, dried ($MgSO_4$) and concentrated *in vacuo* to give *tert*-butyl (3-(2-(2-(3-aminopropoxy)ethoxy)ethoxy)propyl)carbamate **S6** (0.970 g, 3.03 mmol, 75%) as a yellow oil: ^{1}H NMR (600 MHz, $CDCl_3$) δ 5.16 (s, 1H), 3.62–3.50 (m, 12H), 3.20–3.18 (m, 2H), 2.76 (t, *J* = 6.7 Hz, 2H), 1.72–1.68 (m, 4H), 1.40 (s, 9H); ^{13}C NMR 150 MHz, CDCl3) δ 156.2 (C), 78.9 (C), 70.7 (CH_2), 70.7 (CH_2), 70.3 (CH_2), 70.3 (CH_2), 69.7 (CH_2), 69.6 (CH_2), 39.7 (CH_2), 38.6 (CH_2), 33.4 (CH_2), 29.7 (CH_2), 28.6 (CH_3); IR (thin film) 3358, 2927, 2867, 1693, 1518 cm^{-1}.

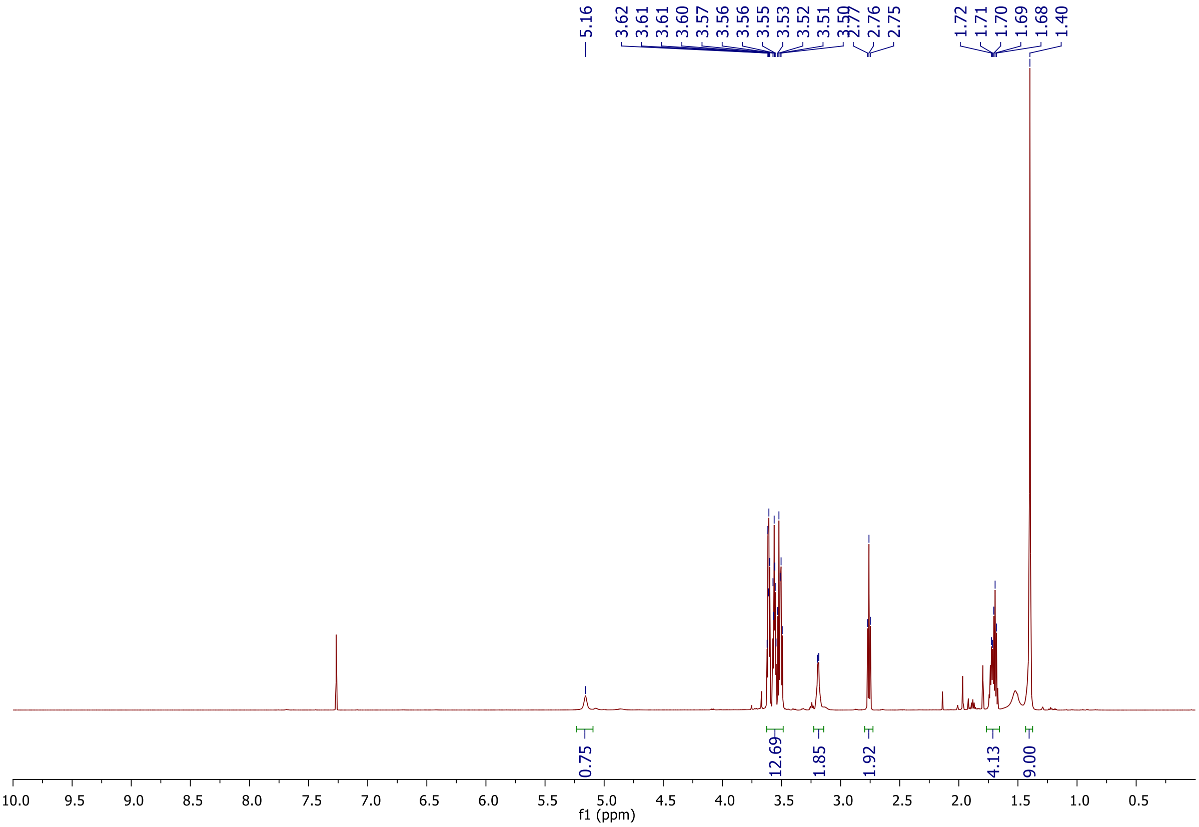

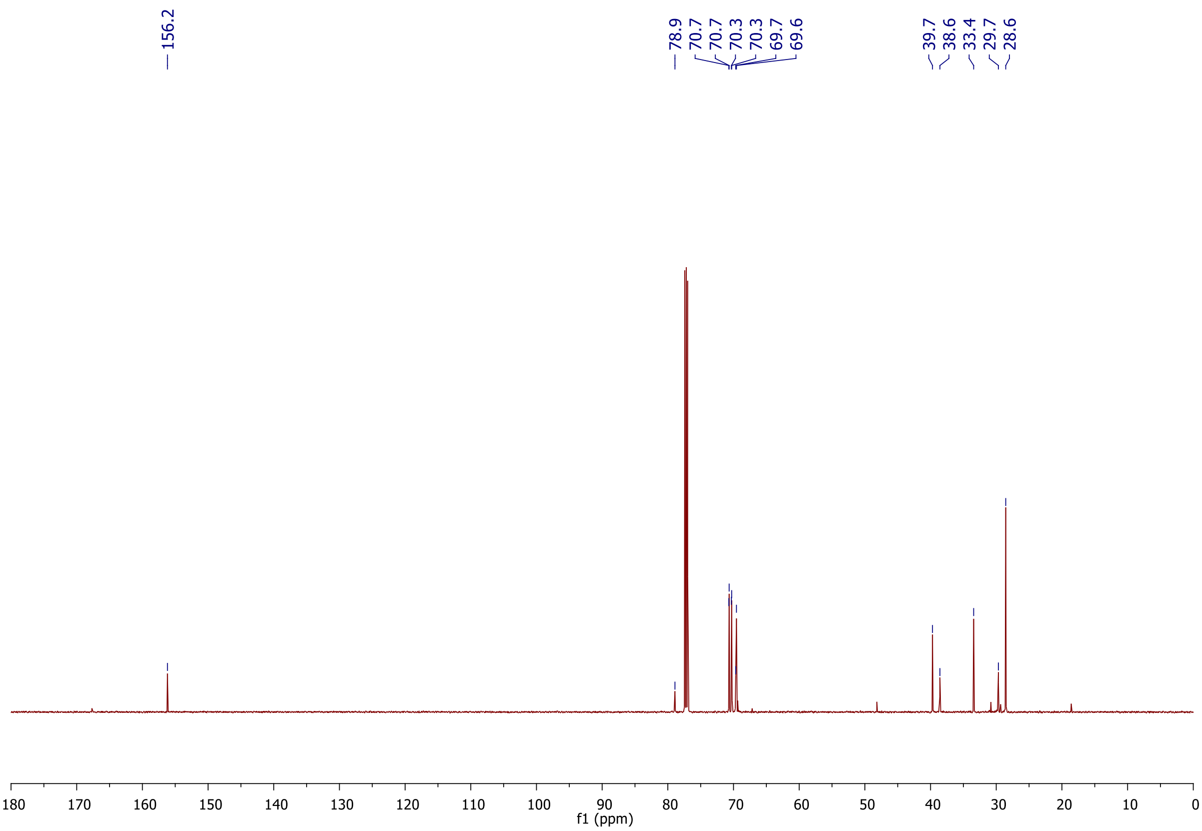

Figure S13 ^{1}H and ^{13}C NMR data for *tert*-butyl (3-(2-(2-(3-aminopropoxy)ethoxy)ethoxy)propyl)carbamate **S6**.

General PD-Amide coupling protocol:

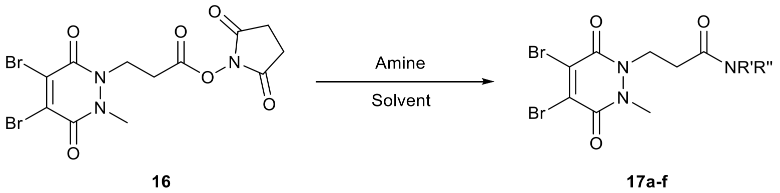

To a solution of 2,5-dioxopyrrolidin-1-yl 3-(4,5-dibromo-2-methyl-3,6-dioxo-3,6-dihydropyridazin-1(2*H*)-yl) propanoate **16** (100 mg, 0.221 mmol, pre-dissolved in solvent (10 mL)), was added amine (0.24 mmol) and the reaction mixture was stirred at 21 °C for 16 h. After this time, the reaction was concentrated *in vacuo* and the crude residue dissolved in $CHCl_3$ (50 mL), and washed with water (2 × 30 mL) and saturated aq. K_2CO_3 (30 mL). The organic layer was then dried ($MgSO_4$) and concentrated *in vacuo.* Purification of the crude residue by flash column chromatography (0% to 10% MeOH/EtOAc) afforded amide products **17a-f** as described in the sections below. Detail of the solvent used in each synthesis is indicated below.

3-(4,5-Dibromo-2-methyl-3,6-dioxo-3,6-dihydropyridazin-1(2*H*)-yl)-*N*-hexylpropanamide (17a)

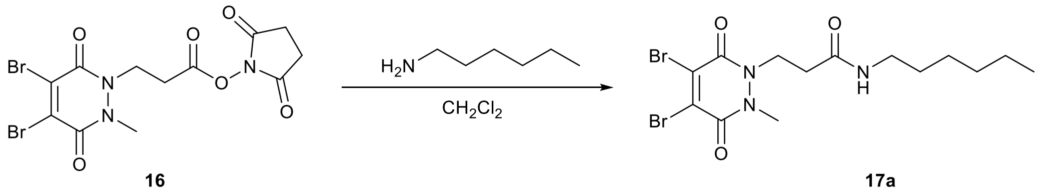

General amide coupling protocol (solvent used: CH_2Cl_2) afforded 3-(4,5-dibromo-2-methyl-3,6-dioxo-3,6-dihydropyridazin-1(2*H*)-yl)-*N*-hexylpropanamide **17a** (76.0 mg, 0.170 mmol, 79%) as a viscous oil: ^{1}H NMR (600 MHz, $CDCl_3$) δ 5.93 (s, 1H), 4.43 (t, *J* = 7.0 Hz, 2H), 3.74 (s, 3H), 3.21 (m, 2H), 2.61 (t, *J* = 7.0 Hz, 2H), 1.47–1.45 (m, 2H), 1.30–1.25 (m, 6H), 0.87 (t, *J* = 6.7 Hz, 3H); ^{13}C NMR (150 MHz, $CDCl_3$) δ 168.9 (C), 153.0 (C), 153.0 (C), 136.5 (C), 135.3 (C), 44.8 (CH_2), 39.9 (CH_2), 35.1 (CH_3), 34.30 (CH_2), 31.5 (CH_2), 29.5 (CH_2), 26.7 (CH_2), 22.7 (CH_2), 14.2 (CH_3); IR (thin film) 3317, 2924, 2854, 1736, 1627, 1572, 1545 cm^{-1}; LRMS (ESI) 442 (50, [M^{81}Br^{81}Br+H]$^+$), 440 (100, [M^{79}Br^{81}Br+H]$^+$) 438 (50, [M^{79}Br^{79}Br+H]$^+$); HRMS (ESI) calcd for $C_{14}H_{22}Br_2N_3O_3$ [M^{79}Br^{81}Br+H]$^+$ 440.0002; observed 439.9998.

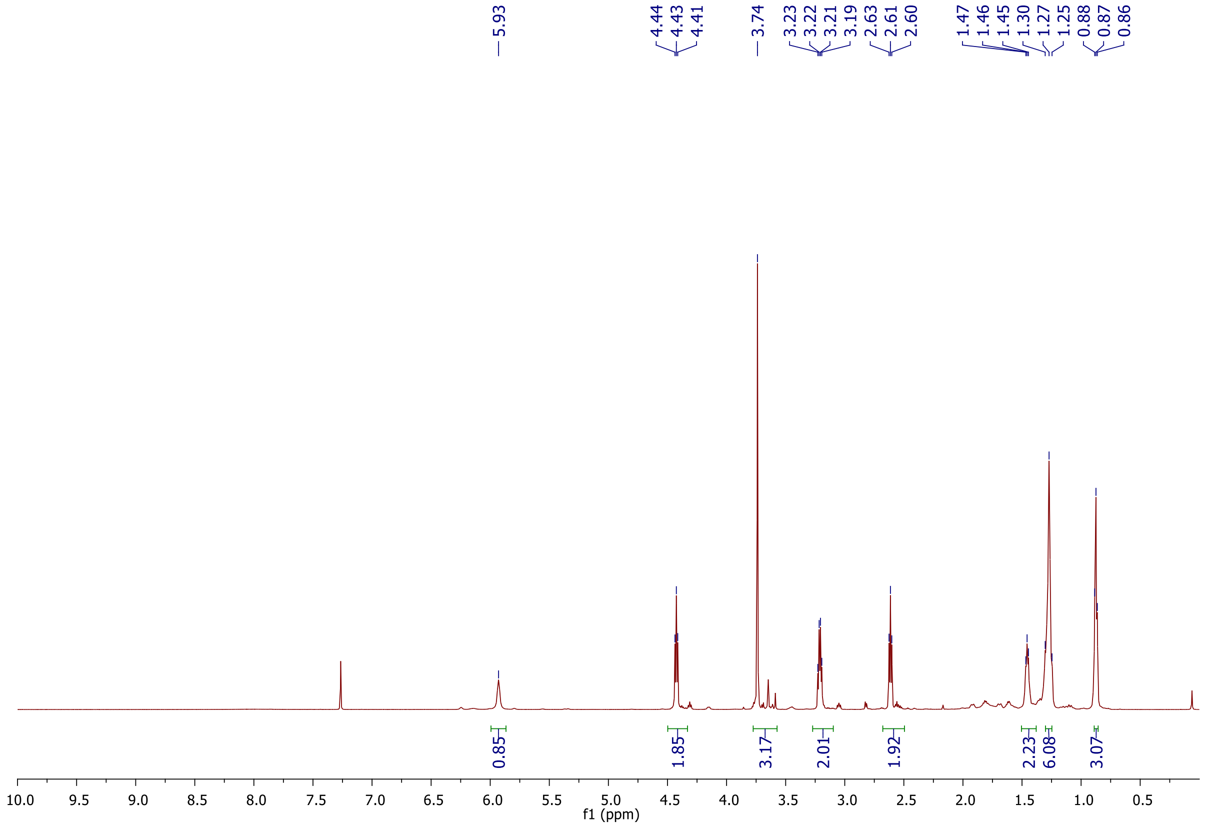

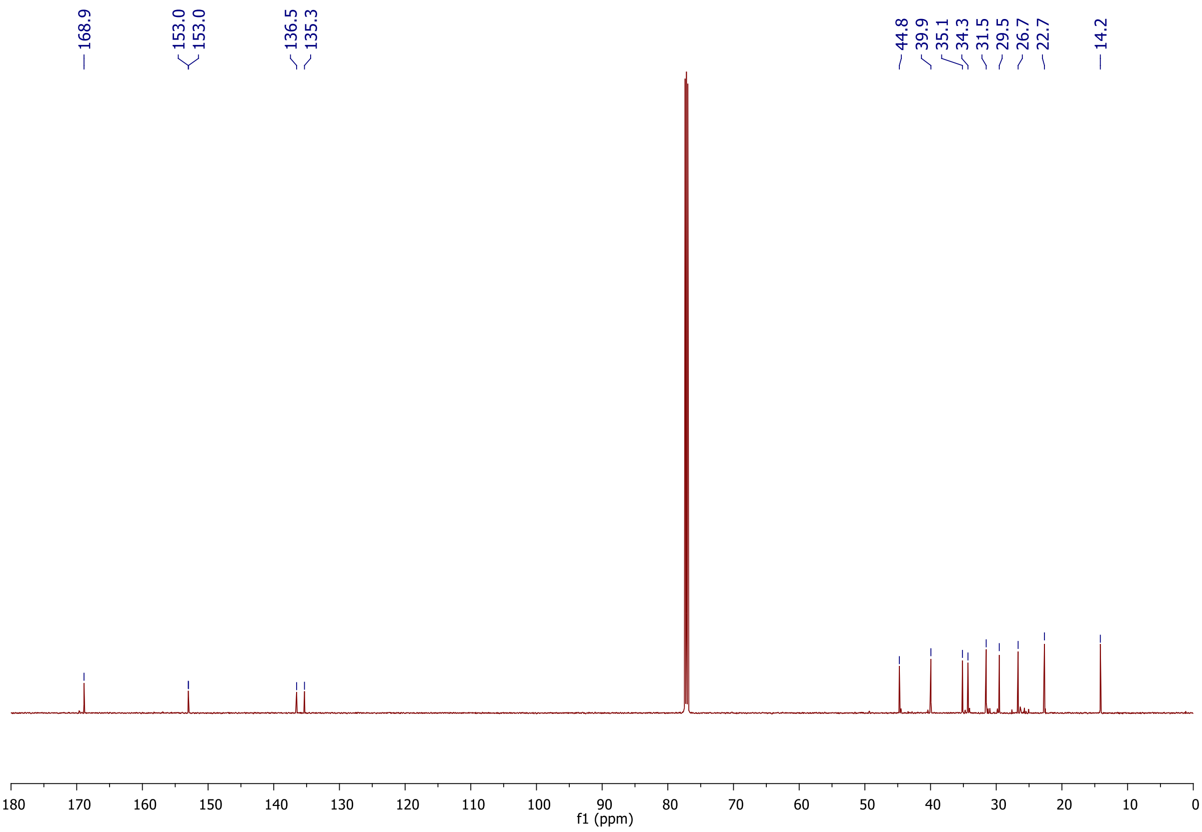

Figure S14 ^{1}H and ^{13}C NMR data for 3-(4,5-dibromo-2-methyl-3,6-dioxo-3,6-dihydropyridazin-1(2*H*)-yl)-*N*-hexylpropanamide **17a**.

3-(4,5-Dibromo-2-methyl-3,6-dioxo-3,6-dihydropyridazin-1(2*H*)-yl)-*N*-(2-(2-hydroxyethoxy)ethyl)propanamide (17b)

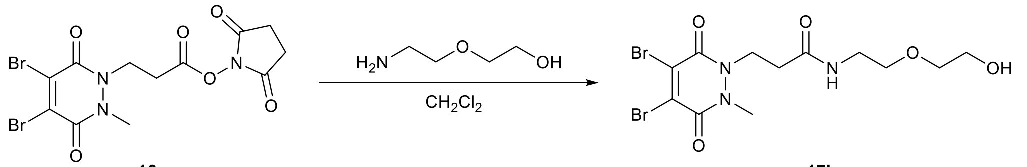

General amide coupling protocol (solvent used: CH_2Cl_2) afforded 3-(4,5-dibromo-2-methyl-3,6-dioxo-3,6-dihydropyridazin-1(2*H*)-yl)-*N*-(2-(2- hydroxyethoxy) ethyl)propanamide **17b** (77.0 mg, 0.150 mmol, 79%) as a light yellow oil: ^{1}H NMR (600 MHz, $CDCl_3$) δ 6.73–6.70 (m, 1H), 4.44 (t, *J* = 7.0 Hz, 2H), 3.75 (m, 2H), 3.73 (s, 3H), 3.58 (m, 2H), 3.55 (t, 5.2 Hz, 2H), 3.44 (m, 2H), 2.63 (t, *J* = 7.0Hz, 2H); ^{13}C NMR (150 MHz, $CDCl_3$) δ 169.3 (C), 153.1 (C), 136.6 (C), 135.4 (C), 72.4 (CH_2), 69.6 (CH_2), 61.9 (CH_2), 44.7 (CH_2), 39.6 (CH_2), 35.2 (CH_3), 34.4 (CH_2); IR (thin film) 3323, 2922, 2854, 1733, 1628, 1571 cm^{-1}; LRMS (ESI) 465 (60, [M^{79}Br^{81}Br+Na]$^+$), 446 (50, [M^{81}Br^{81}Br+H]$^+$), 444 (100, [M^{79}Br^{81}Br+H]$^+$), 442 (50, [M^{79}Br^{79}Br+H]$^+$); HRMS (ESI) calcd for $C_{12}H_{18}Br_2N_3O_3$ [M^{79}Br^{81}Br+H]$^+$ 443.9584; observed 443.9588.

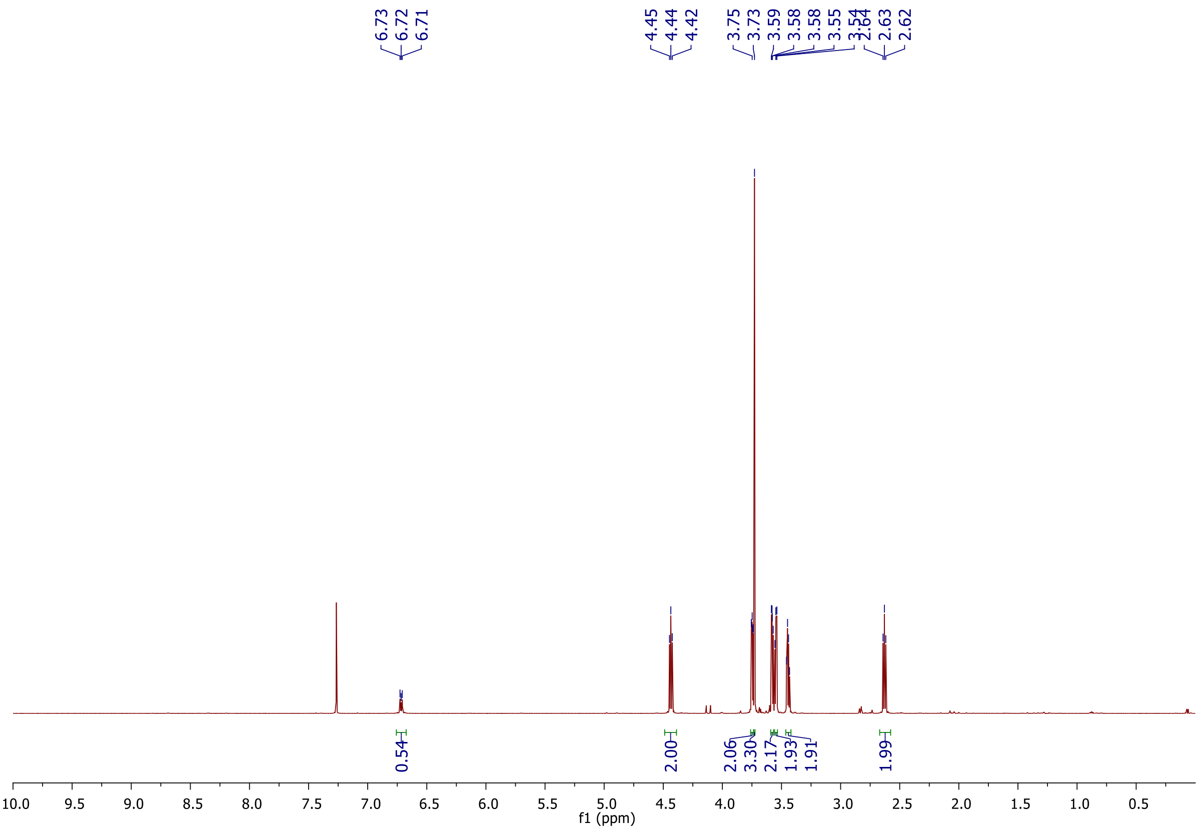

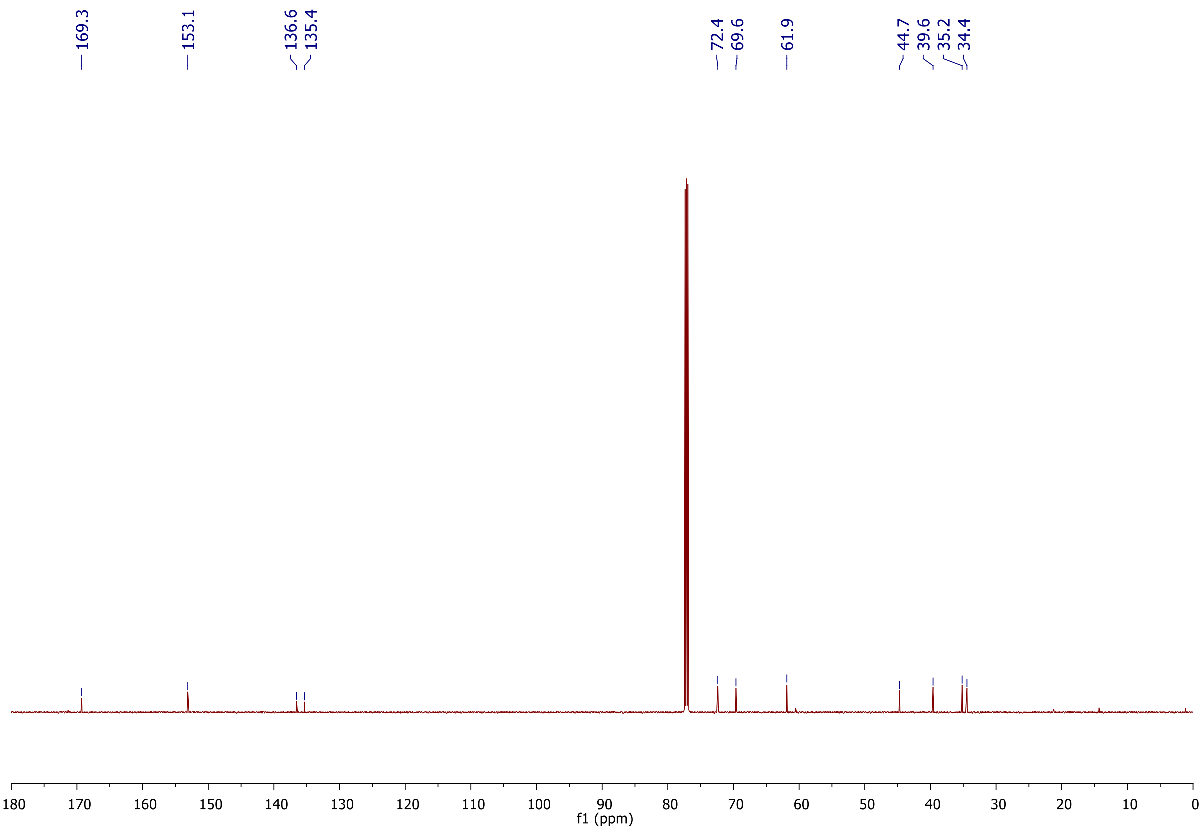

Figure S15 ^{1}H and ^{13}C NMR data for 3-(4,5-dibromo-2-methyl-3,6-dioxo-3,6-dihydropyridazin-1(2*H*)-yl)-*N*-(2-(2-hydroxyethoxy)ethyl)propenamide **17b**.

4,5-Dibromo-1-methyl-2-(3-morpholino-3-oxopropyl)-1,2-dihydropyridazine-3,6-dione (17c)

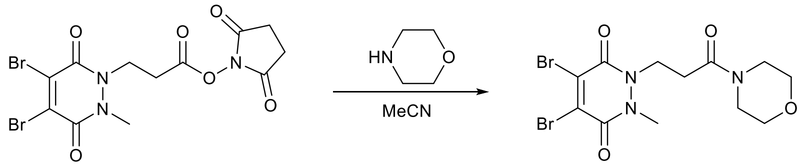

General amide coupling protocol (solvent used: MeCN) afforded 4,5-dibromo-1-methyl-2-(3-morpholino-3-oxopropyl)-1,2-dihydropyridazine-3,6-dione **17c** (71.0 mg, 0.170 mmol, 76%) as a yellow oil: ^{1}H NMR (600 MHz, $CDCl_3$, rotamers) δ 4.44 (t, *J* = 7.4 Hz, 2H), 3.74 (s, 3H), 3.69–3.65 (m, 4H), 3.63–3.57 (m, 2H), 3.45 (m, 2H), 2.75 (t, *J* = 7.4 Hz, 2H); ^{13}C NMR (150 MHz, $CDCl_3$, rotamers) δ 169.0 (C), 167.8 (C), 153.1 (C), 153.0 (C), 136.4 (C), 135.5 (C), 66.8 (CH_2), 66.6 (CH_2), 46.0 (CH_2), 44.4 (CH_2), 42.1 (CH_2), 35.1 (CH_3), 30.6 (CH_2), 25.4 (CH_2); IR (thin film) 2921, 2856, 1733, 1628, 1574 cm^{-1}; LRMS (ESI) 428 (50, [M^{81}Br^{81}Br+H]$^{+}$), 426 (100, [M^{79}Br^{81}Br+H]$^{+}$), 424 (50, [M^{79}Br^{79}Br+H]$^{+}$); HRMS (ESI) calcd for $C_{12}H_{16}Br_2N_3O_4$ [M^{79}Br^{81}Br+H]$^{+}$ 425.9482; observed 425.9474.

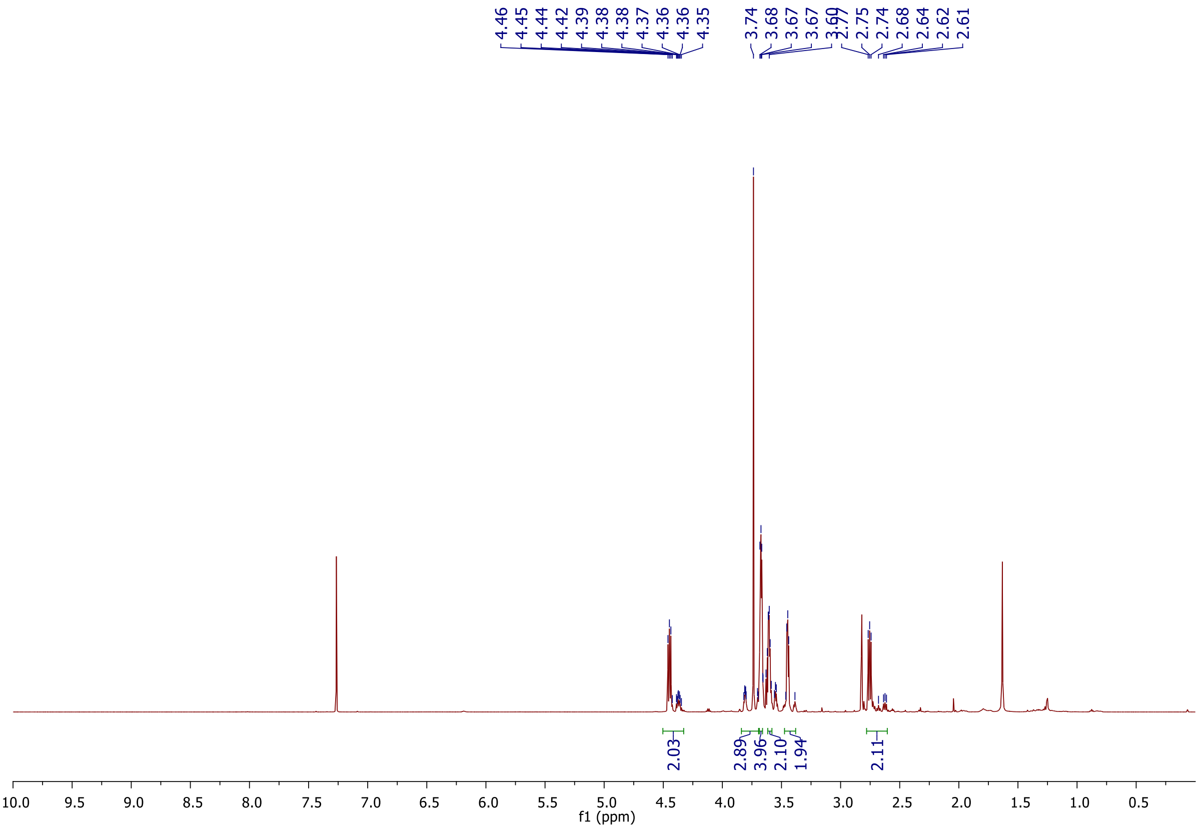

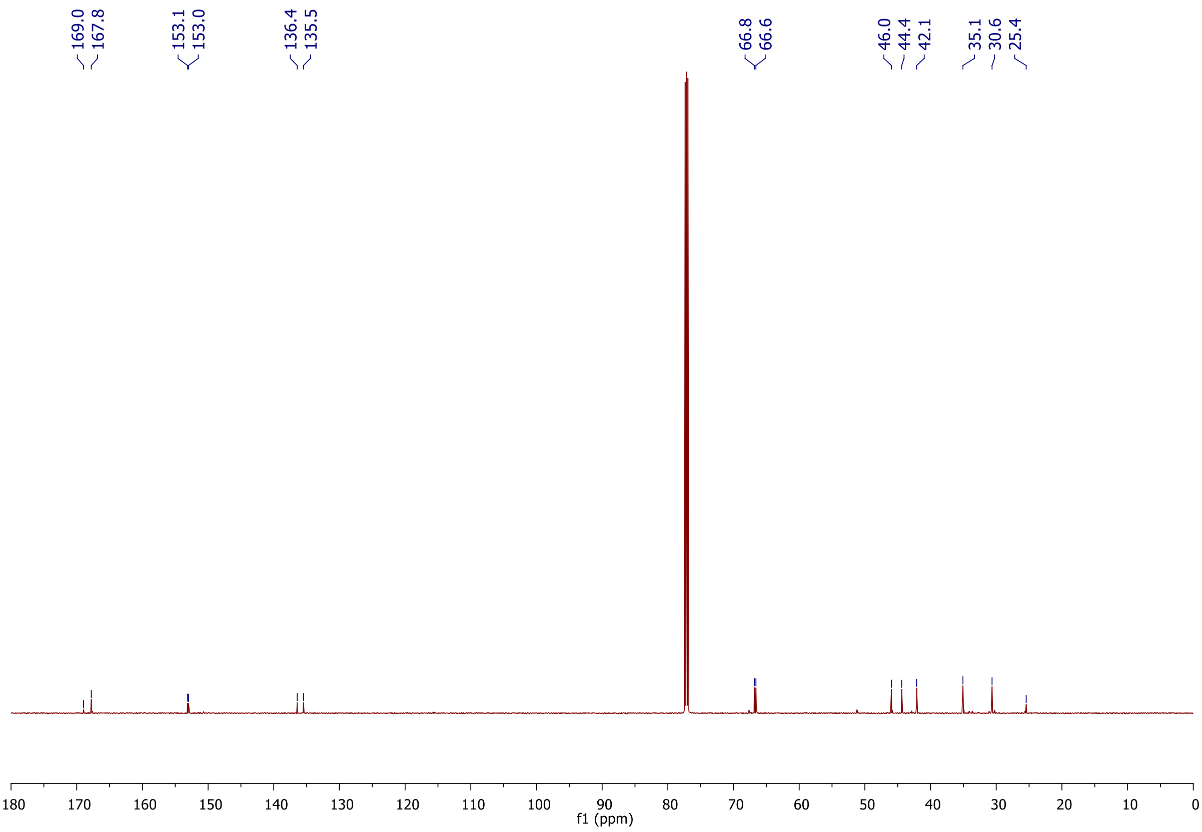

Figure S16 ^{1}H and ^{13}C NMR data for 4,5-dibromo-1-methyl-2-(3-morpholino-3-oxopropyl)-1,2-dihydropyridazine-3,6-dione **17c**.

3-(4,5-Dibromo-2-methyl-3,6-dioxo-3,6-dihydropyridazin-1(2*H*)-yl)-*N*-(prop-2-yn-1-yl)propanamide (17d)

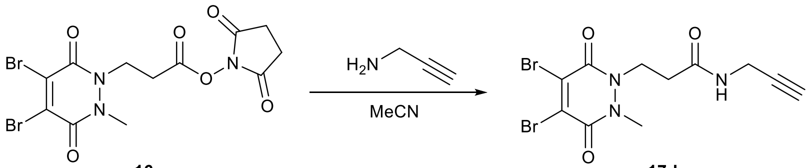

16 17d

General amide coupling protocol (solvent used: MeCN) afforded 3-(4,5-dibromo-2-methyl-3,6-dioxo-3,6-dihydropyridazin-1(2*H*)-yl)-*N*-(prop-2-yn-1-yl)propenamide **17d** (75.0 mg, 0.190 mmol, 87%) as an orange oil: ^{1}H NMR (600 MHz, DMSO-d_6) δ 8.46 (t, *J* = 5.2 Hz, 1H), 4.26 (t, *J* = 7.2 Hz, 2H), 3.82 (m, 2H), 3.55 (s, 3H), 3.09 (t, *J* = 2.3 Hz, 1H), 2.48 (t, *J* = 7.2 Hz, 2H); ^{13}C NMR (150 MHz, DMSO-d_6) δ 169.0 (C), 152.6 (C), 152.3 (C), 135.3 (C), 135.0 (C), 80.8 (C), 73.2 (CH), 43.7 (CH_2), 34.7 (CH_3), 32.8 (CH_2), 27.9 (CH_2); IR (thin film) 3294, 2932, 2855, 1734, 1632, 1571, 1542 cm^{-1}; LRMS (ESI) 411 (50, [M^{79}Br^{81}Br+NH$_4$]$^+$), 396 (50, [M^{81}Br^{81}Br+H]$^+$), 394 (100, [M^{79}Br^{81}Br+H]$^+$), 392 (50, [M^{79}Br^{79}Br+H]$^+$); HRMS (ESI) calcd for $C_{11}H_{12}Br_2N_3O_3$ [M^{79}Br^{81}Br+H]$^+$ 393.9219; observed 393.9218.

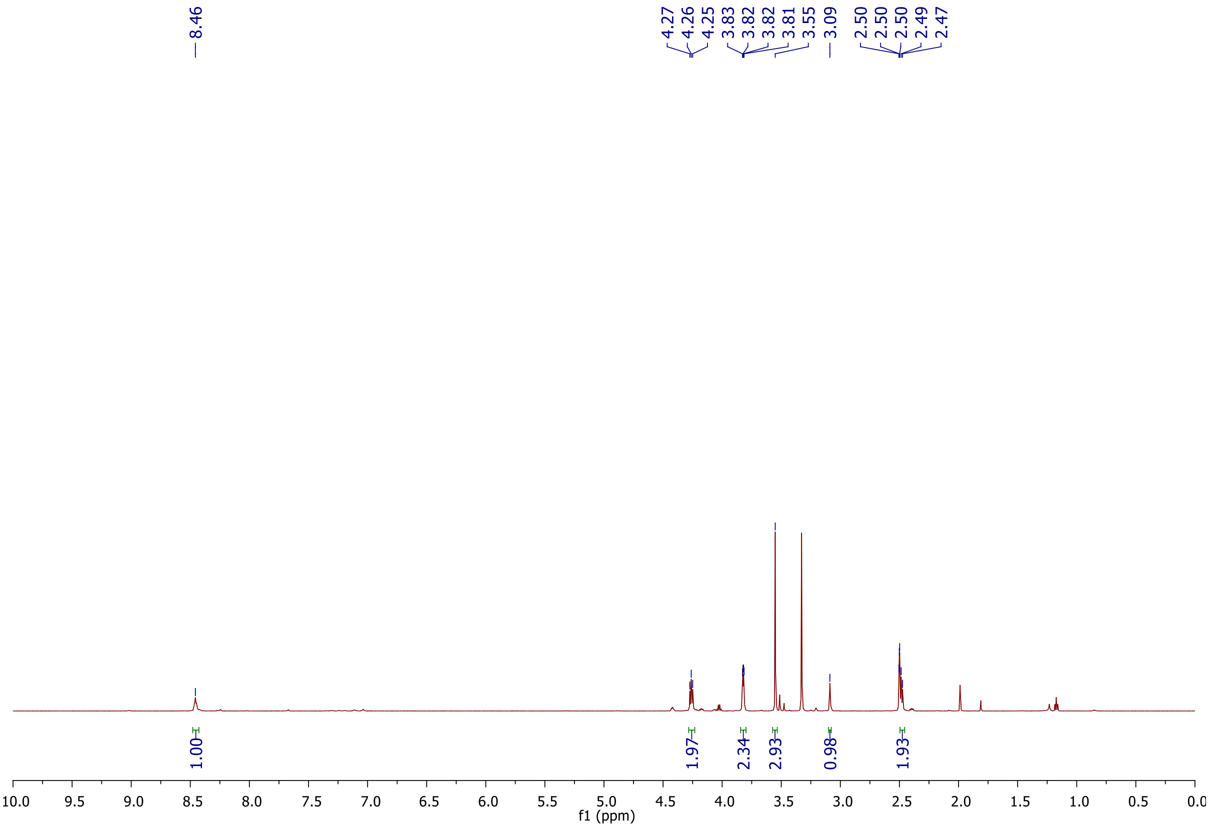

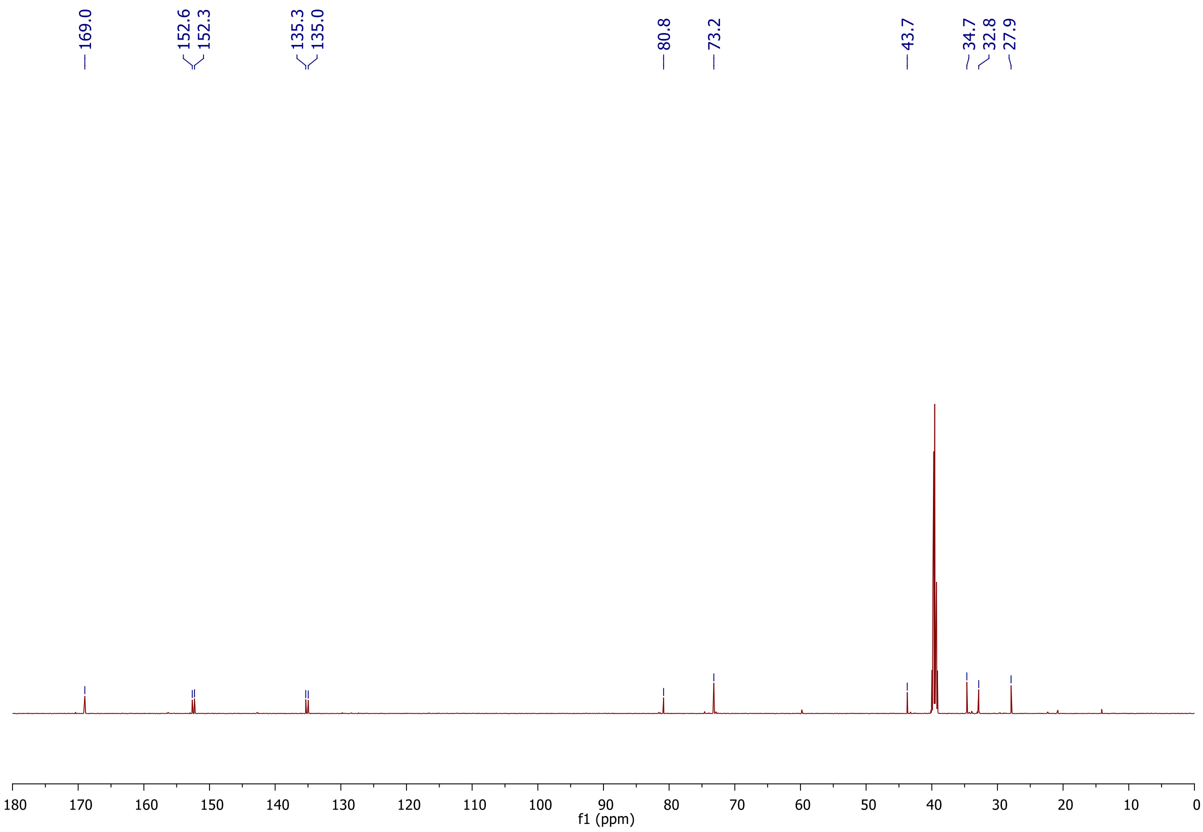

Figure S17 ^{1}H and ^{13}C NMR data for 3-(4,5-dibromo-2-methyl-3,6-dioxo-3,6-dihydropyridazin-1(2*H*)-yl)-*N*-(prop-2-yn-1-yl) propenamide **17d**.

((1*R*,8*S*,9*s*)-Bicyclo[6.1.0]non-4-yn-9-yl)methyl (2-(2-(2-(3-(4,5-dibromo-2-methyl-3,6-dioxo-3,6-dihydropyridazin-1(2*H*)-yl)propanamido)ethoxy)ethoxy)ethyl) carbamate (17e)

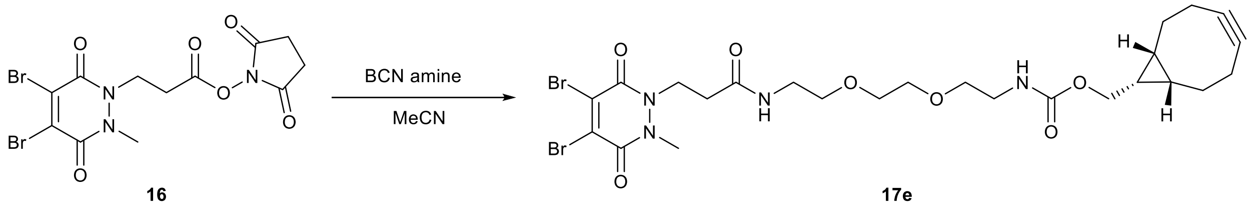

General amide coupling protocol (solvent used: MeCN) afforded ((1*R*,8*S*,9*S*)-Bicyclo[6.1.0] non-4-yn-9-yl)methyl (2-(2-(2-(3-(4,5-dibromo-2-methyl-3,6-dioxo-3,6-dihydropyridazin-1(2*H*)-yl)propanamido)ethoxy)ethoxy)ethyl) carbamate **17e** (105 mg, 0.160 mmol, 72%) as a yellow oil: ^{1}H NMR (600 MHz, $CDCl_3$, rotamers) δ 7.84 (s, 0.5H), 6.34 (s, 0.5H), 5.82 (s, 0.5H), 5.29 (s, 0.5H), 4.44 (t, *J* = 6.6 Hz, 2H), 4.14–4.12 (m, 4H), 3.73–3.71 (m, 3H), 3.60–3.38 (m, 12H), 2.62 (t, *J* = 6.6 Hz, 2H), 2.27 (m, 6H), 1.61–1.57 (m, 2H), 1.39–1.24 (m, 2H), 0.96–0.94 (m, 2H); ^{13}C NMR (150 MHz, $CDCl_3$, rotamers) δ 169.1 (C), 156.9 (C), 153.1 (C), 153.0 (C), 136.4 (C), 135.5 (C), 98.9 (C), 70.4 (CH_2), 70.3 (CH_2), 69.7 (CH_2), 63.0 (CH_2), 44.6 (CH_2), 40.8 (CH_2), 39.5 (CH_2), 35.1 (CH_3), 34.1 (CH_2), 29.3 (CH_2), 29.2 (CH_2), 21.6 (CH_2), 20.2 (CH_2), 17.9 (CH), 14.3 (CH); IR (thin film) 3329, 2920, 2858, 1708, 1630, 1572, 1534 cm^{-1}; LRMS (ESI), 687 (50, $[M^{81}Br^{81}Br+Na]^+$) 685 (100, $[M^{79}Br^{81}Br+Na]^+$), 683 (50, $[M^{79}Br^{79}Br+Na]^+$), 663 (60, $[M^{79}Br^{81}Br+H]^+$); HRMS (ESI) calcd for $C_{25}H_{35}Br_2N_4O_7$ $[M^{79}Br^{81}Br+H]^+$ 663.0847; observed 663.0846.

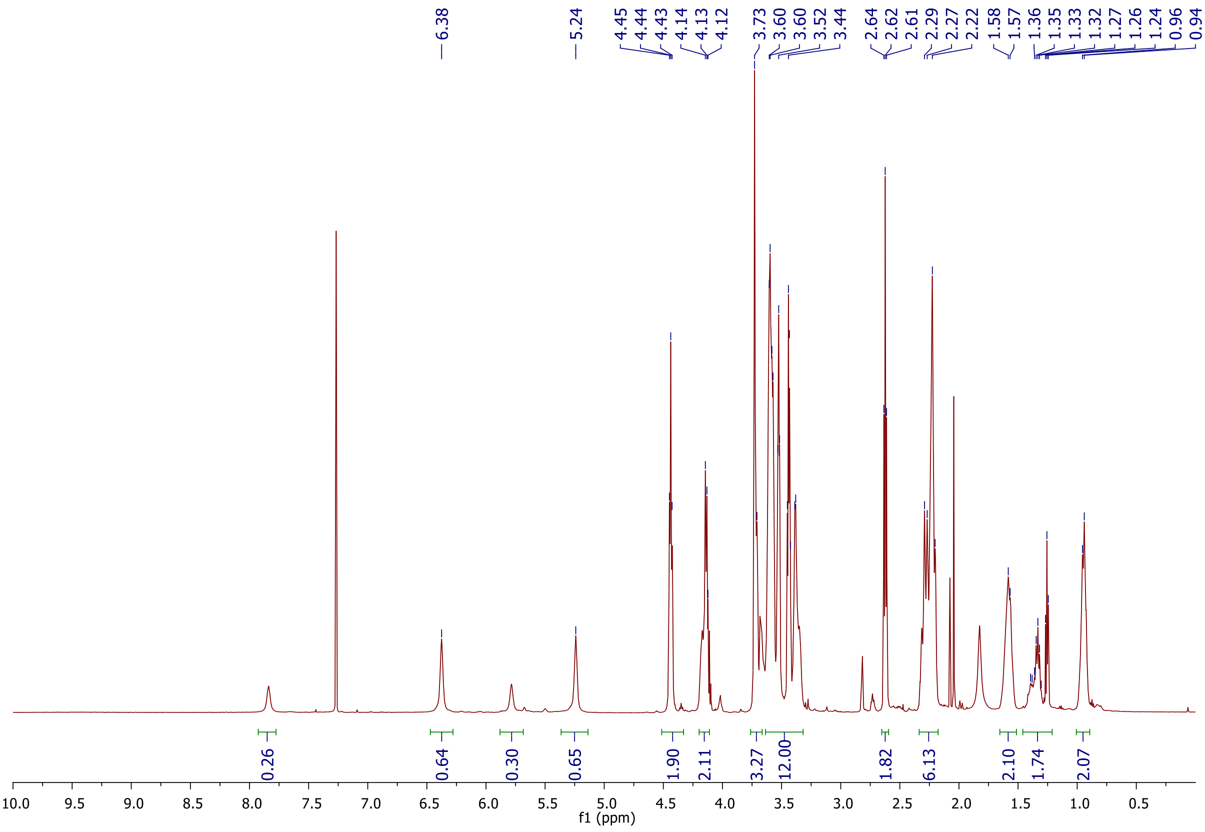

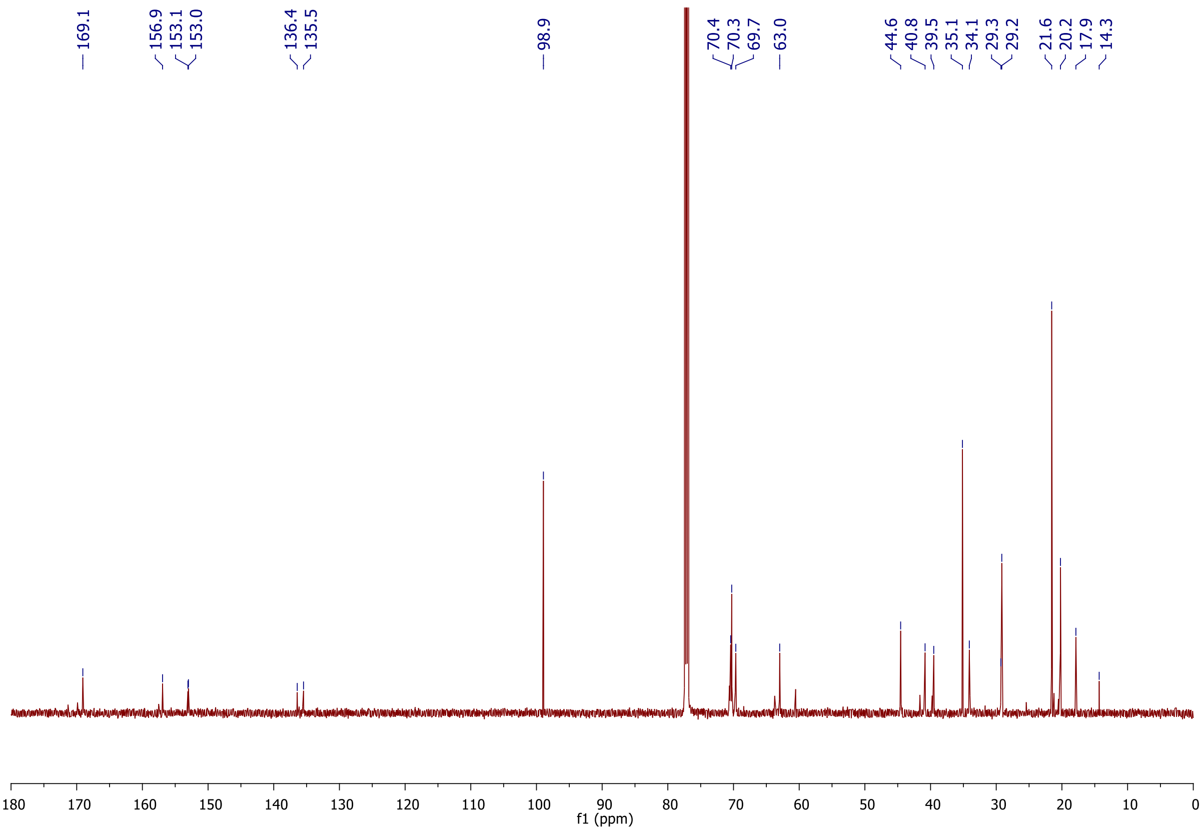

Figure S18 ^{1}H and ^{13}C NMR data for ((1R,8S,9s)-bicyclo[6.1.0]non-4-yn-9-yl)methyl (2-(2-(2-(3-(4,5-dibromo-2-methyl-3,6-dioxo-3,6-dihydropyridazin-1(2*H*)-yl)propanamido)ethoxy)ethoxy)ethyl) carbamate **17e**.

tert-Butyl (17-(4,5-dibromo-2-methyl-3,6-dioxo-3,6-dihydropyridazin-1(2*H*)-yl)-15-oxo-4,7,10-trioxa-14-azaheptadecyl)carbamate (17f)

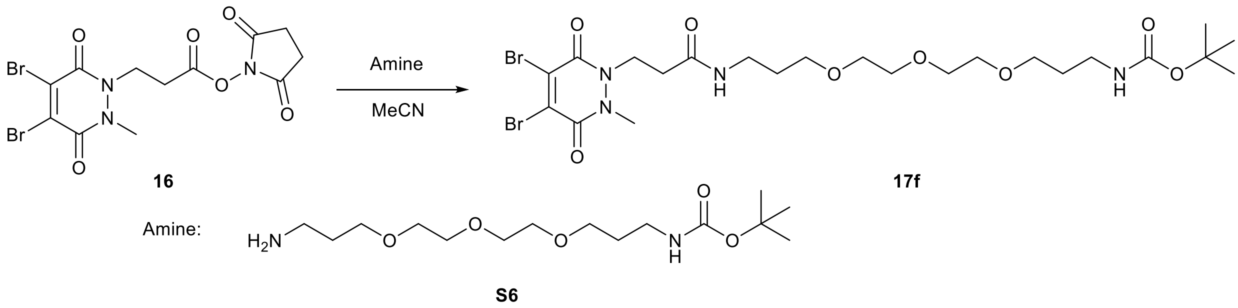

General amide coupling protocol (solvent used: MeCN) afforded *tert*-butyl (17-(4,5-dibromo-2-methyl-3,6-dioxo-3,6-dihydropyridazin-1(2*H*)-yl)-15-oxo-4,7,10-trioxa-14-azaheptadecyl)carbamate **17f** (112 mg, 0.170 mmol, 78%) as a yellow oil: ^{1}H NMR (600 MHz, $CDCl_3$) δ 6.76 (s, 1H), 4.97 (s, 1H), 4.44 (t, *J* = 6.9 Hz, 2H), 3.73 (s, 3H), 3.63–3.51 (m, 12H), 3.39–3.34 (m, 2H), 3.21–3.20 (s, 2H), 2.56 (t, *J* = 6.9 Hz, 2H), 1.75–1.71 (m, 4H), 1.43 (s, 9H); ^{13}C NMR 150 MHz, $CDCl_3$) δ 169.0 (C), 156.2 (C), 153.1 (C), 152.9 (C), 136.4 (C), 135.5 (C), 79.1 (C), 70.5 (CH_2), 70.5 (CH_2), 70.3 (CH_2), 70.2 (CH_2), 70.1 (CH_2), 69.5 (CH_2), 44.5 (CH_2), 38.7 (CH_2), 38.5 (CH_2), 38.3 (CH_2), 35.2 (CH_3), 29.8 (CH_2), 28.6 (CH_3), 25.6 (CH_2); IR (thin film) 3336, 2929, 2869, 1778, 1736, 1702, 1634 cm^{-1}; LRMS (ESI) 678 (50, [M^{81}Br^{81}Br+NH$_4$]$^+$), 676 (100, [M^{79}Br^{81}Br+NH$_4$]$^+$), 674 (50, [M^{79}Br^{79}Br+NH$_4$]$^+$), 659 (50, [M^{79}Br^{81}Br+H]$^+$); HRMS (ESI) calcd for $C_{23}H_{39}Br_2N_4O_8$ [M^{79}Br^{81}Br+H]$^+$ 659.1111; observed 659.1106.

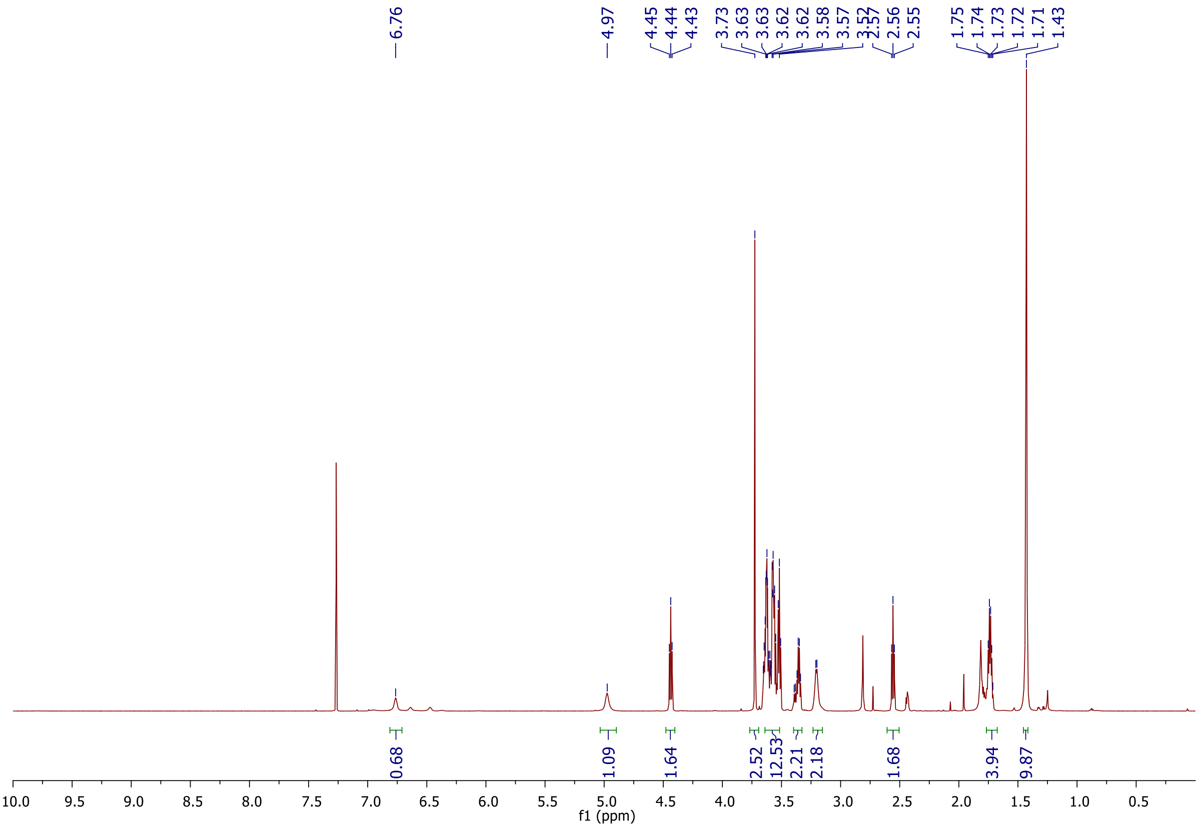

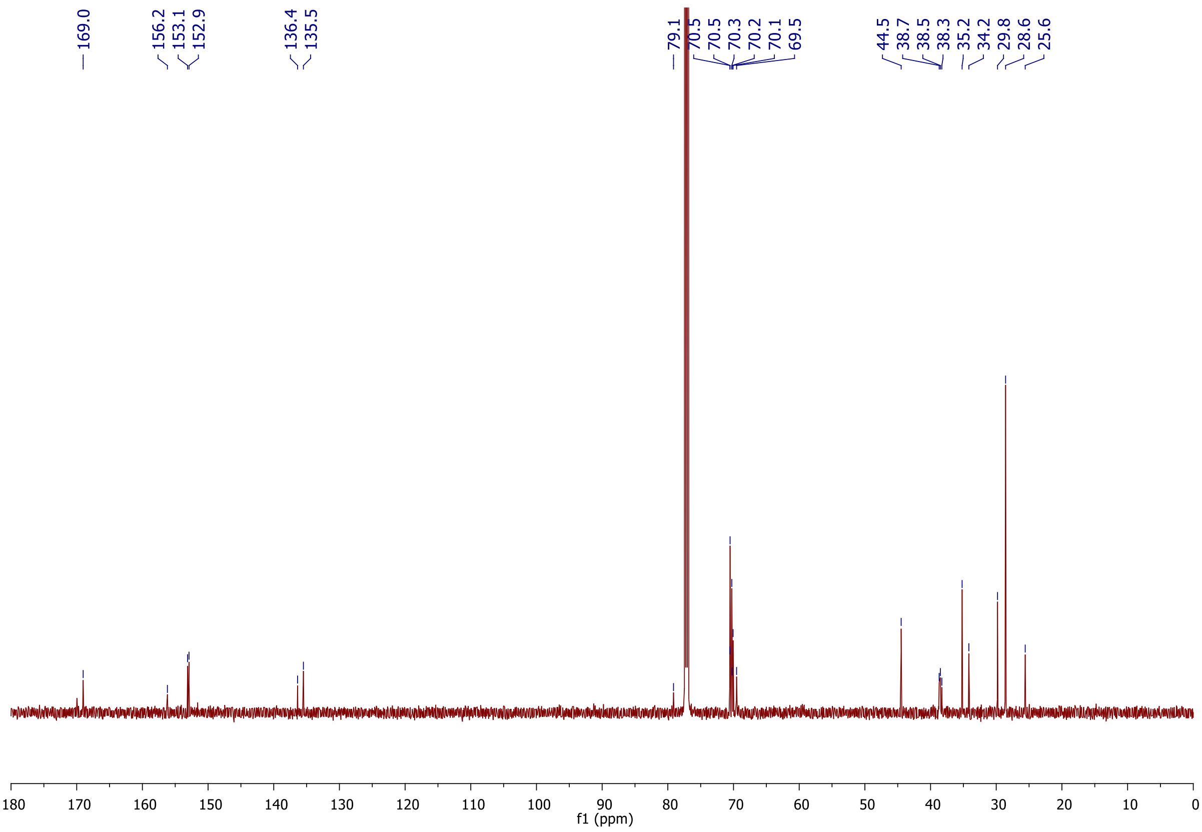

Figure S19 ^{1}H and ^{13}C NMR data for *tert*-butyl (17-(4,5-dibromo-2-methyl-3,6-dioxo-3,6-dihydropyridazin-1(2*H*)-yl)-15-oxo-4,7,10-trioxa-14-azaheptadecyl)carbamate **17f**.

Chemical biology

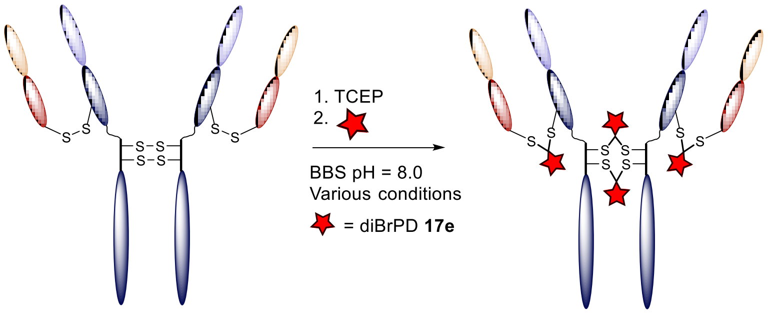

All samples were analysed by SDS-PAGE, densitometry, and UV-vis spectrometry. See manuscript Figs. 3a and 3b for SDS-PAGE and densitometry analysis.

Protocol comparison for the functional bridging of trastuzumab 18 with diBrPD 17e.

Step-wise protocol

TCEP·HCl (1.0 µL, 10 mM in deionised water, 10 eq.) was added to a solution of trastuzumab **18** (50 µL, 20 µM) in BBS (25 mM sodium borate, 25 mM NaCl, 0.5 mM EDTA, 2% DMSO, pH 8.0) and the solution incubated at 37 °C for 1.5 h. After this time the solution was buffer swapped into BBS *via* ultrafiltration (4 × 10000 MWCO, VivaSpin®¸ GE Healthcare), and the concentration adjusted to 20 µM. DiBrPD **17e** (1.6, µL, 10 mM in DMSO, 20 eq.) was added to the solution of reduced trastuzumab **18** (40 µL, 20 µM in BBS) at 21 °C and the solution incubated for 16 h. Excess reagents were removed by ultrafiltration (6 × 10000 MWCO, VivaSpin®¸ GE Healthcare) into PBS (pH = 7.4).

Sequential protocol

TCEP·HCl (1.0 µL, 10 mM in deionised water, 10 eq.) was added to a solution of trastuzumab **18** (50 µL, 20 µM) in BBS (25 mM sodium borate, 25 mM NaCl, 0.5 mM EDTA, 2% DMSO, pH 8.0) and the solution incubated at 37 °C for 1.5 h. The solution was cooled to 21 °C, diBrPD **17e** (2.0, µL, 10 mM in DMSO, 20 eq.) was added and the solution incubated for 16 h. Excess reagents were removed by ultrafiltration (6 × 10000 MWCO, VivaSpin®¸ GE Healthcare) into PBS (pH = 7.4).

In-situ protocol

DiBrPD **17e** (2.0, µL, 10 mM in DMSO, 20 eq.) was added to a solution of trastuzumab **18** (50 µL, 20 µM) in BBS (25 mM sodium borate, 25 mM NaCl, 0.5 mM EDTA, 2% DMSO, pH 8.0) and the solution incubated at 21 °C for 1 h. TCEP·HCl (1.0 µL, 10 mM in d.d water at 21 °C, 10 eq.) was added and the solution incubated at 21 °C for 16 h. Excess reagents were removed by ultrafiltration (6 × 10000 MWCO, VivaSpin®¸ GE Healthcare) into PBS (pH = 7.4).

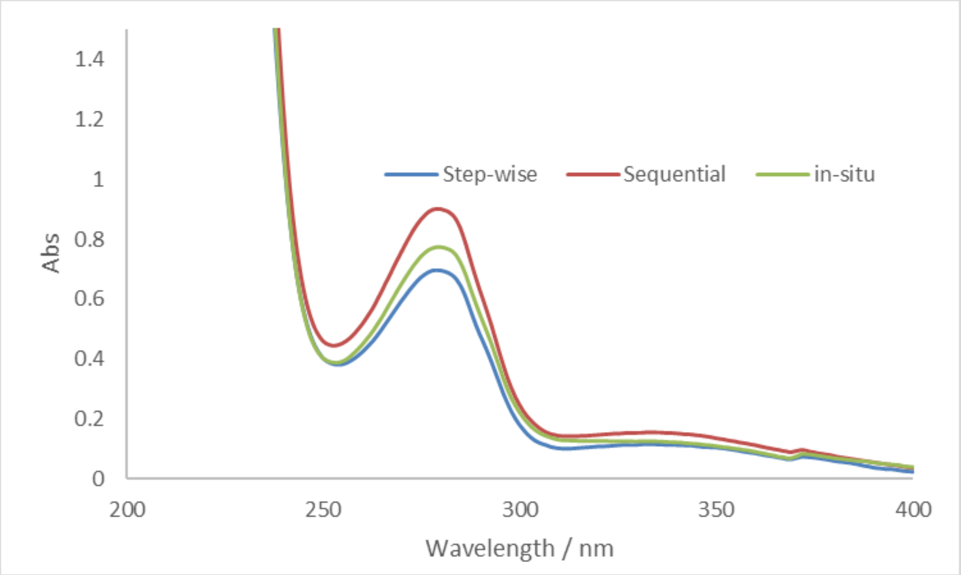

Figure S20 UV-vis spectra of step-wise (PDAR 4.1), sequential (PDAR 4.1) and *in-situ* (PDAR 3.9) protocols for the functional rebridging of trastuzumab **18** with diBrPD **17e**. PDAR was calculated as previously described.[1]

Temperature comparison for the functional bridging of trastuzumab 18 with diBrPD 17e.

DiBrPD **17e** (2.0, µL, 10 mM in DMSO, 20 eq.) was added to a solution of trastuzumab **18** (50 µL, 20 µM) in BBS (25 mM sodium borate, 25 mM NaCl, 0.5 mM EDTA, 2% DMSO, pH 8.0) and the solution incubated at 4 °C, 8 °C, 21 °C or 37 °C for 1 h. TCEP·HCl (1.0 µL, 10 mM in deionised water at 4 °C, 8 °C, 21 °C, or 37 °C, 10 eq.) was added and the solution incubated at 4 °C, 8 °C, 21 °C or 37 °C for 16 h. Excess reagents were removed by ultrafiltration (6 × 10000 MWCO, VivaSpin®¸ GE Healthcare) into PBS (pH = 7.4).

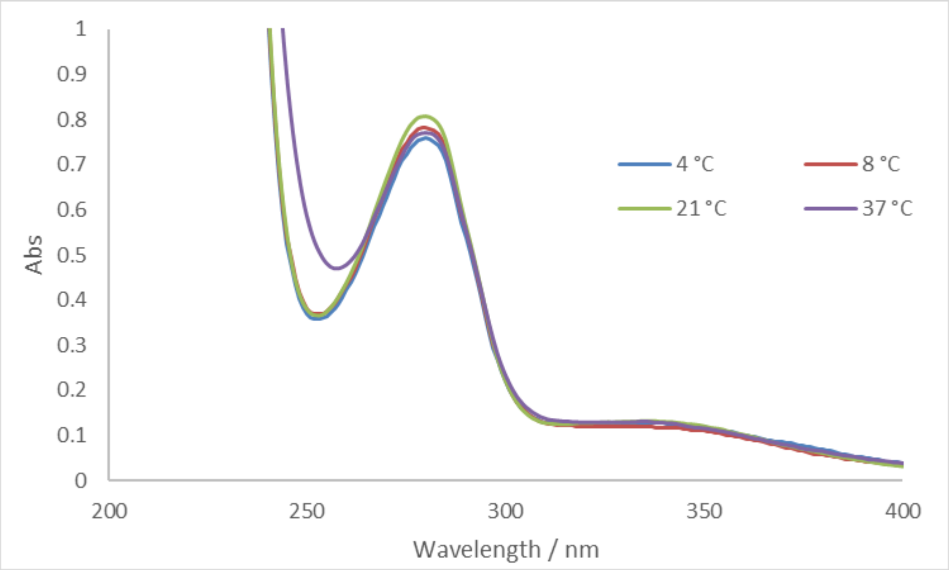

Figure S21 UV-vis spectra of 4 °C (PDAR 4.2), 8 °C (PDAR 3.8), 21 °C (PDAR 3.9), and 37 °C (PDAR 4.2) protocols for the functional rebridging of trastuzumab **18** with diBrPD **17e**. PDAR was calculated as previously described.[1]

Equivalents comparison for the functional bridging of trastuzumab 18 with diBrPD 17e.

DiBrPD **17e** (2.0 µL, 7.5 mM, 10 mM, 15 mM, 20 mM, 25 mM in DMSO, 15 eq., 20 eq., 30 eq., 40 eq., 50 eq.) was added to solutions of trastuzumab **18** (50 µL, 20 µM) in BBS (25 mM sodium borate, 25 mM NaCl, 0.5 mM EDTA, 2% DMSO, pH 8.0) and the solutions incubated at 4 °C for 1 h. TCEP·HCl (1.0 µL, 10 mM in d.d water at 4 °C, 10 eq.) was added and the solution incubated at 4 °C for 16 h. Excess reagents were removed by ultrafiltration (6 × 10000 MWCO, VivaSpin®¸ GE Healthcare) into PBS (pH = 7.4).

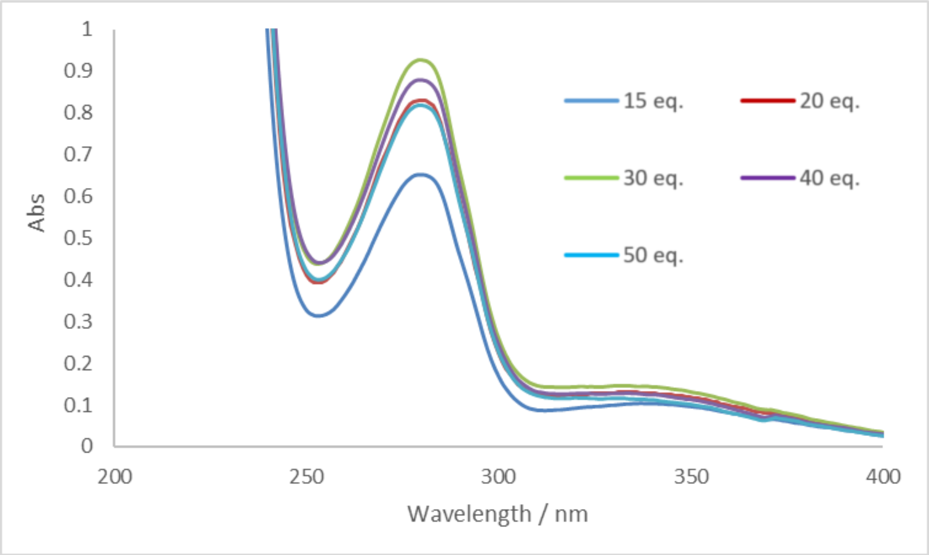

Figure S22 UV-vis spectra of the functional rebridging of trastuzumab **18** with diBrPD **17e** at different molar equivalents: 15 eq (PDAR 3.9); 20 eq (PDAR 3.9); 30 eq (PDAR 3.9); 40 eq PDAR (3.6); 50 (PDAR 3.3). PDAR was calculated as previously described.[1]

Optimised protocol for the functional bridging of trastuzumab 18 with diBrPDs 17a-e.

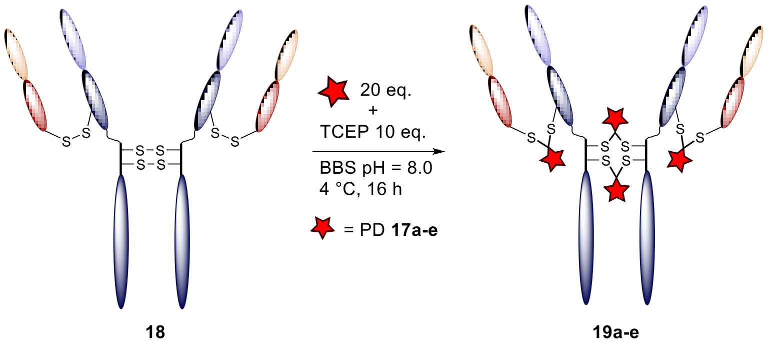

Below is a general protocol which was repeated for diBrPDs **17a-e**.

DiBrPD **17a-e** (2.0, µL, 20 mM in DMSO, 20 eq.) was added to a solution of trastuzumab **18** (50 µL, 40 µM) in BBS (25 mM sodium borate, 25 mM NaCl, 0.5 mM EDTA, 2% DMSO, pH 8.0) and the solution incubated at 4 °C for 1 h. TCEP·HCl (1.0 µL, 20 mM in deionised water at 4 °C, 10 eq.) was added and the solution incubated at 4 °C for 16 h. Excess reagents were removed by ultrafiltration (6 × 10000 MWCO, VivaSpin®¸ GE Healthcare) into PBS (pH = 7.4). All samples were analysed by SDS-PAGE, densitometry, and UV-vis spectrometry. See manuscript Fig. 4a and 4b for SDS-PAGE and densitometry analysis.

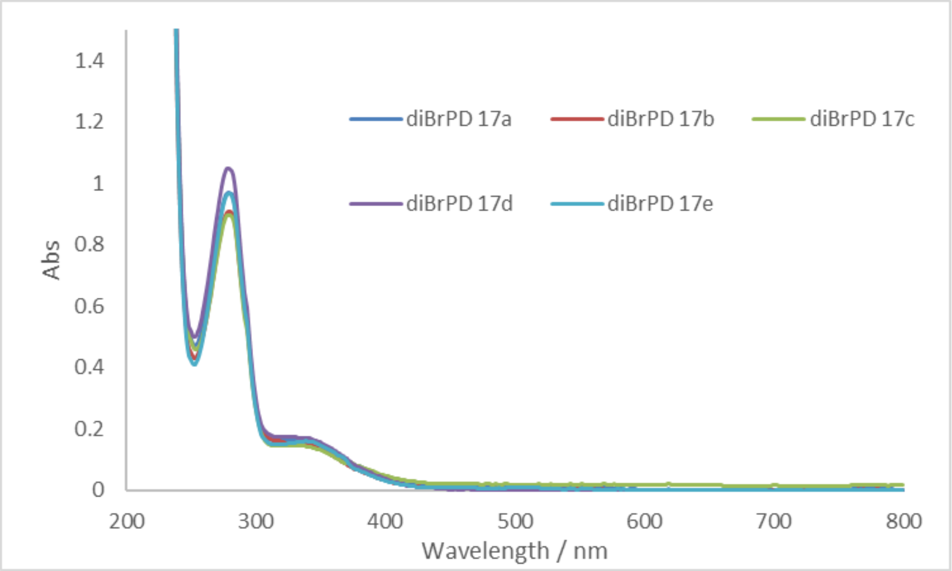

Figure S23 UV-vis spectra of the functional rebridging of trastuzumab **18** with diBrPDs **17a-e** under optimal conditions: diBrPD **17a** (PDAR 4.2); diBrPD **17b** (PDAR 4.1); diBrPD **17c** (PDAR 4.0); diBrPD **17d** (PDAR 4.0); diBrPD **17e** (PDAR 4.0). PDAR was calculated as previously described.[1]

HER2 ELISA for Trastuzumab 18 and Trastuzumab-PD conjugates 19a-e

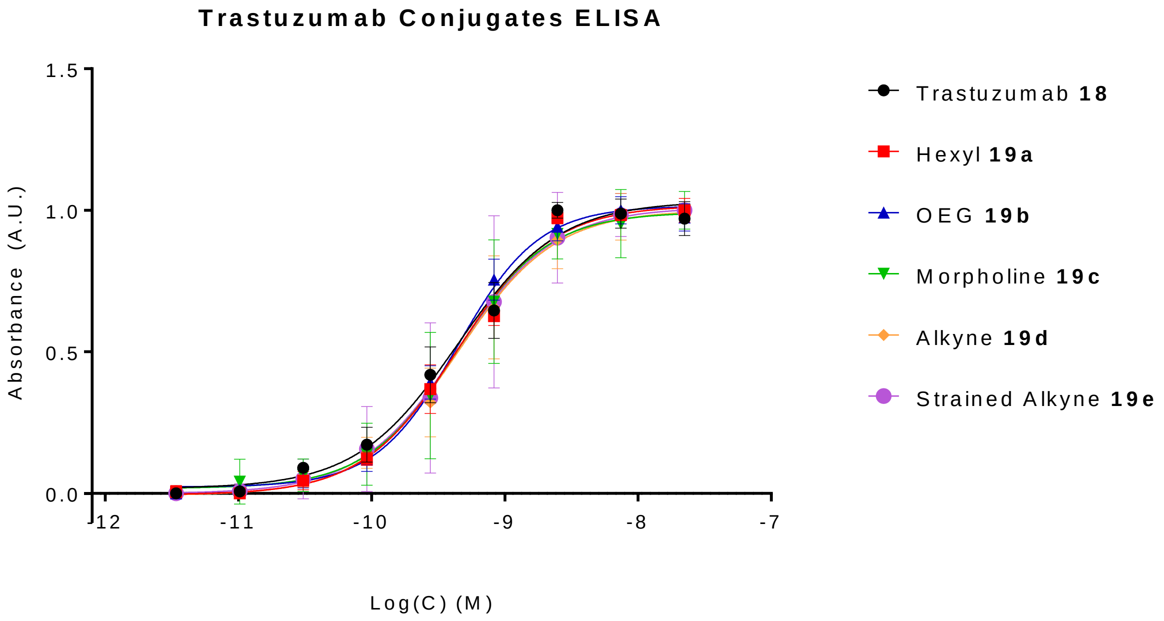

Figure S24 Binding activity to HER 2 of trastuzumab **18** and trastuzumab-PD conjugates: **18** (IC_{50} = 0.41 nM), **19a** (IC_{50} = 0.49 nM), **19b** (IC_{50} = 0.38 nM), **19c** (IC_{50} = 0.47 nM), **19d** (IC_{50} = 0.50 nM), **19e** (IC_{50} = 0.47 nM), over concentration ranges from 23.3–0.003 nM, Abs measured at 650 nm.

References

1 V. Chudasama, M. E. B. Smith, F. F. Schumacher, D. Papaioannou, G. Waksman, J. R. Baker and S. Caddick, *Chem. Commun*., 2011, **47**, 8781–8783.

2 M. T. W. Lee, A. Maruani, D. A. Richards, J. R. Baker, S. Caddick and V. Chudasama, *Chem. Sci.*, 2017, **8**, 2056–2060.

3 J. C. Slootweg, S. Van Der Wal, H. C. Quarles Van Ufford, E. Breukink, R. M. J. Liskamp and D. T. S. Rijkers, Bioconjug. *Chem.*, 2013, **24**, 2058–2066.
